# Identification of *MATE* Family and Characterization of *GmMATE13* and *GmMATE75* in Soybean’s Response to Aluminum Stress

**DOI:** 10.3390/ijms25073711

**Published:** 2024-03-26

**Authors:** Pengxiang Gao, Rongrong Han, Hui Xu, Yunmin Wei, Yongxiong Yu

**Affiliations:** 1 Center for Plant Environmental Sensing, College of Life Sciences and Oceanography, Shenzhen University, Shenzhen 518060, China; gaopengxiang518@163.com; 2 Key Laboratory of Optoelectronic Devices and Systems of Ministry of Education and Guangdong Province, College of Optoelectronic Engineering, Shenzhen University, Shenzhen 518060, China; 3 College of Animal Science and Technology, Southwest University, Chongqing 400715, China; 19891214rong@163.com (R.H.); xhswcvc@163.com (H.X.); yuyongxiong8@126.com (Y.Y.)

**Keywords:** aluminum tolerance, citrate transporter, expression analysis, gene family, Tamba black soybean

## Abstract

The multidrug and toxic compound extrusion (MATE) proteins are coding by a secondary transporter gene family, and have been identified to participate in the modulation of organic acid exudation for aluminum (Al) resistance. The soybean variety *Glycine max* “Tamba” (TBS) exhibits high Al tolerance. The expression patterns of *MATE* genes in response to Al stress in TBS and their specific functions in the context of Al stress remain elusive. In this study, 124 *MATE* genes were identified from the soybean genome. The RNA-Seq results revealed significant upregulation of *GmMATE13* and *GmMATE75* in TBS upon exposure to high-dose Al^3+^ treatment and both genes demonstrated sequence homology to citrate transporters of other plants. Subcellular localization showed that both proteins were located in the cell membrane. Transgenic complementation experiments of *Arabidopsis* mutants, *atmate,* with *GmMATE13* or *GmMATE75* genes enhanced the Al tolerance of the plant due to citrate secretion. Taken together, this study identified *GmMATE13* and *GmMATE75* as citrate transporter genes in TBS, which could improve citrate secretion and enhance Al tolerance. Our findings provide genetic resources for the development of plant varieties that are resistant to Al toxicity.

## 1. Introduction

More than 50% of arable land consists of acidic soil worldwide. The severity of acidity is increasing due to multiple factors, including over agriculture, fertilizer utilization, and acid rain caused by air pollution [1]. In acidic soils, the presence of soluble aluminum (Al) leads to the formation of toxic Al^3+^ from aluminosilicate clays. This toxicity inhibits plant root elongation and growth, impairing water and nutrient uptake, ultimately resulting in low yield and poor growth of the plant [2]. Different plant species employ various mechanisms to reduce Al^3+^ toxicity and enhance survival under high Al^3+^ conditions. Two major mechanisms, namely internal tolerance and exclusion have been extensively studied [3]. The internal tolerance mechanism involves detoxifying Al^3+^ in the cytosol by forming nontoxic organic acid (OA)-Al complexes and sequestering Al^3+^ in vacuoles [4]. The exclusion mechanism involves chelating Al^3+^ in the rhizosphere using organic acid anions to form nontoxic OA-Al chelates, which limit Al^3+^ uptake by roots, preventing Al^3+^ interaction with sensitive root sites [5]. Under Al stress, plants enhance resistance by upregulating root secretion of OAs such as citrate, malate, and oxalate, which aid in removing Al^3+^ from roots. Consequently, a better understanding of the genes involved in plant Al resistance will contribute to the discovery of novel genotypes suitable for cultivation in acidic soils.

In the past decades, numerous genes and signaling pathways have been identified in relation to the exudation of OAs induced by Al stress. For example, the first gene identified was *TaALMT1* (aluminum-activated malate transporter) in wheat [6], followed by *SbMATE* (multidrug and toxic compound extrusion) in sorghum [7] and *HvAACT1* (aluminum-activated citrate transported 1) in barley, all of which are responsible for the secretion of OAs in response to aluminum [8]. These transporters belong to the *MATE* family. Since then, multiple *MATE* genes have been found to participate in the modulation of OAs’ exudation for Al resistance, making the *MATE* family the most widely categorized multidrug efflux transporter family [3]. MATE transporters are widely present in bacteria, fungi, plants, and mammals, and they possess a unique structure with 12 transmembrane (TM) helices [9]. Homologous genes encoding MATE proteins are required for Al resistance and detoxification and are localized in the root epidermis cells [10]. Furthermore, MATE transporters also play a key role in a wide range of biological functions, including the accumulation of alkaloids and flavonoids, iron homeostasis and translocation, plant diseases and virus resistance, and plant hormone signaling [11].

Based on genome-wide analysis, numerous putative MATE transporters have been identified in multiple plant species, including 49 in maize [12], 67 in tomato [13], 56 in *Arabidopsis thaliana* [14,15], 45 in *Oryza sativa* [14], and 117 in *Glycine max* [16]. Among these species, *Glycine max* has the highest number of MATE transporters. As an important oil-bearing crop, soybean is extensively cultivated in acidic soils with a long history. This has led to the development of diverse Al tolerance genes and mechanisms in soybeans. When exposed to Al stress, the roots of soybeans secrete organic acids (OAs) as a means of Al detoxification [17]. Tolerant soybean genotypes exhibit higher OA secretion compared to sensitive cultivars. Previous studies have indicated that several *MATE* genes encode proteins that participate in the Al-induced secretion of OAs in soybeans as a response to Al toxicity. *GsMATE* from *Glycine soja* exhibits the highest expression level in roots and enhances resistance to Al stress [3]. *GmFRD3b* has higher expression levels in the iron-efficient cultivar than in the iron-inefficient line, indicating a similar function to *AtFRD3* in facilitating the efflux of citrate into the xylem [18]. Additionally, another study showed upregulation of *GmMATE75* in an Al-tolerant genotype called Jiyu 70, suggesting its involvement in Al resistance [19]. Therefore, conducting further studies on *GmMATEs* from different soybean phenotypes would be valuable in enhancing our understanding of how soybeans cope with Al stress.

The Tamba black soybean (TBS) genotype is renowned for its remarkable Al tolerance attributed to its ability to secrete citrate in response to Al stress [20]. In the present study, we aimed to investigate the functions of the *MATE* gene family in TBS in response to Al stress. We identified the *MATE* gene family and evaluated the gene expression patterns of these *MATE* family genes through RNA-seq analysis following Al treatment. Furthermore, we characterized the function and subcellular localization of the sensitive *GmMATE genes* through citrate transport activity assays and subcellular location detection methods. We also analyzed the phenotypes of *Arabidopsis* plants overexpressing these genes to gain insights into the mechanisms. Overall, our findings demonstrate which *MATE* genes are involved in Al tolerance and how they enhance Al tolerance in *Glycine max* “Tamba”.

## 2. Results

### 2.1. Identification of MATE Genes in the Soybean Genome

Through Blast searches and domain (Pfam: PF01554) prediction, 124 soybean *MATE* genes were ultimately identified. The genes were named *GmMATE1-124* according to Liu et al. [16] and their physical location on the chromosome. The proteins encoded by these genes exhibit lengths ranging from 80 to 593 amino acids, molecular weights spanning from 8.71 to 64.28 kD, and predicted isoelectric points varying between 5.13 and 9.70 (Table S1).

**Figure 1:**
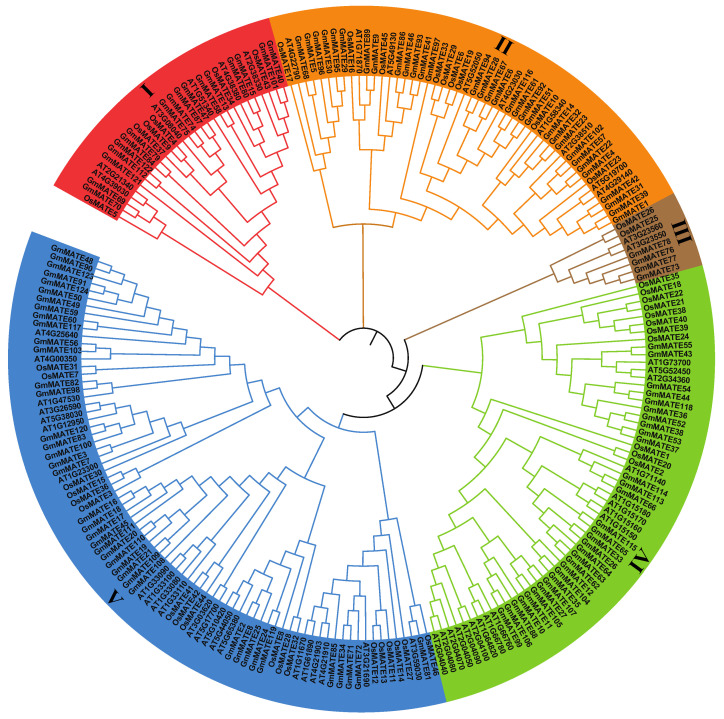
The phylogenetic tree of the *MATE* (multidrug and toxic compound extrusion) family from *Arabidopsis thaliana*, *Oryza sativa*, and *Glycine max*. The tree was constructed with MEGA 6.0 using the maximum likelihood (ML) method. Bootstrap values in percentages are 1000 replicates. Different subfamilies are highlighted using different colors: group I in red, group II in orange, group III in deep yellow, group IV in green, and group V in blue.

A comparison was conducted among soybean MATE protein sequences, along with 45 *Arabidopsis* and 56 rice MATE protein sequences. The resulting phylogenetic tree categorized all MATE proteins into five subfamilies (Figure 1), with soybean MATE protein family members distributed among all these subfamilies. The fifth subfamily comprised the highest count of soybean MATE proteins, with 40 numbers, while the second and fourth group contains 32 and 31 soybean MATE proteins respectively.

### 2.2. Gene Structure of the GmMATE Genes

**Figure 2:**
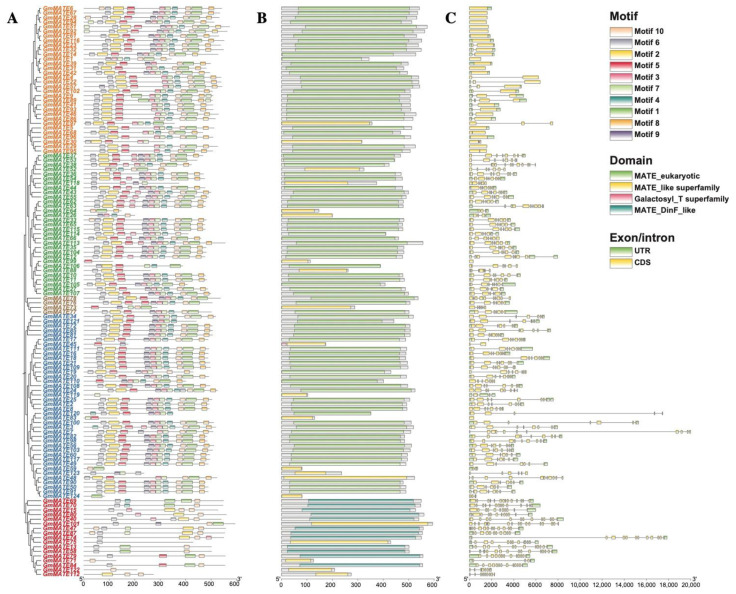
Gene structure, domain, and conserved motif analysis of *GmMATE* genes. (**A**) Distributions of conserved motifs in *GmMATE* genes. The sequence information of the motifs was listed in Table S2. (**B**) MATE and MATE-like domains of *GmMATE* genes. (**C**) Introns and exons on *GmMATE* genes.

The evolutionary progression of a gene family is chiefly evident in the diversity of gene structures and alterations in conserved motifs. Utilizing the MEME online prediction tool, the conserved motifs in soybean MATE proteins were identified, as illustrated in Figure 2A. Ten conserved sequences were detected and denoted as motifs 1–10. The motif sequence was listed in Table S2. The first group of GmMATE proteins had fewer motifs than other groups in general. The majority of the members in groups II–V shared 7–10 motifs. All members of group II were equipped with motif 3. The existence of a protein domain in the *MATE* genes was searched in the NCBI Conserved Domain Search. As shown in Figure 2B, all of the identified GmMATE proteins contain MATE-related domains. The exon–intron structure of *GmMATE* genes was analyzed using the annotation information of the soybean genome (Figure 2C). Members of group II had a smaller number of exons, with approximately 1–3 exons. However, members of groups III, IV, and V typically possessed 6–8 exons, with a few exceptions among those with shorter sequences, which may have a lower number of exons such as *GmMATE59* and *GmMATE83*. Group I members generally included a higher number of exons of over seven. Whereas, an exception was observed in *GmMATE71*, which contained only four exons.

### 2.3. Gene Location of the GmMATE Genes on the Chromosome

**Figure 3:**
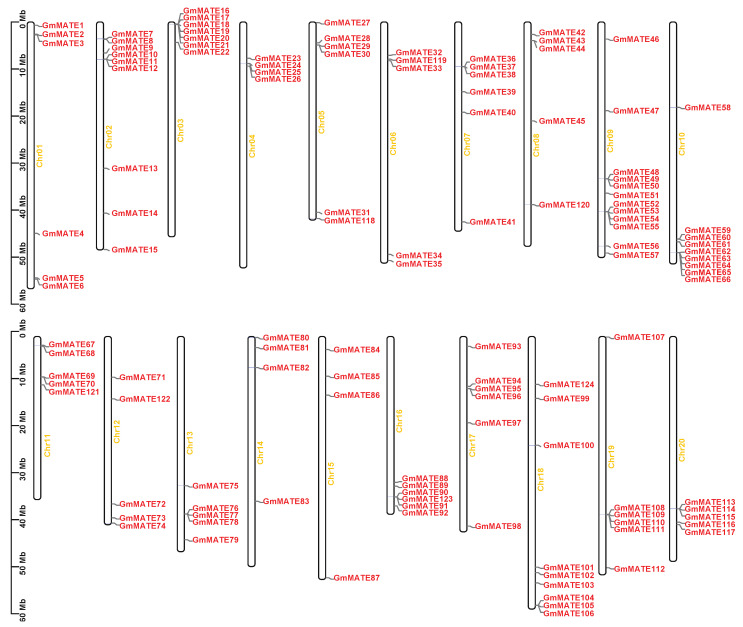
Chromosomal location of *GmMATE* genes. Chr01–20 represent the 20 chromosomes of soybean.

The 124 *GmMATE* genes have been mapped to all 20 soybean chromosomes (Figure 3). However, their distribution across individual chromosomes appears to be uneven. Chromosome 9 exhibits the highest density of *GmMATE* genes, with a total of 12 members spanning from *GmMATE 46* to *GmMATE 57*. Meanwhile chromosomes 2, 10, and 18 also have a high density of *MATE genes*, each harboring nine *GmMATE* genes. In contrast, chromosomes 4, 14, and 15 contain a lower number of *GmMATE* genes, with only four identified on each chromosome.

### 2.4. The Expression of GmMATE Genes on TBS under Al^3+^ Stress

**Figure 4:**
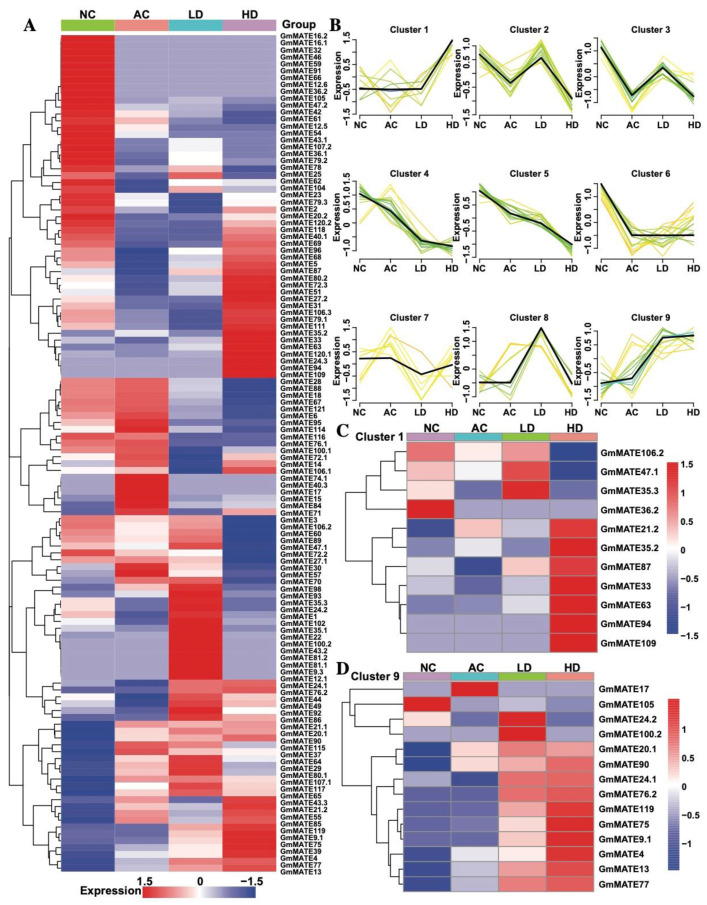
The expression of *GmMATE* genes in Tamba black soybean under Al stress. (**A**) The heatmap of *MATE* gene expression in neutral control (NC), acidic control (AC), low dose (LD, 10 μmol/L), and high dose (HD, 50 μmol/L) AlCl_3_ groups. (**B**) Time series analysis of *GmMATE* genes under Al stress. (**C**,**D**) Heatmap of the expression of genes belonging to cluster 1 and cluster 9.

We identified the *GmMATE* genes in the transcriptome data to assess their expression patterns under Al stress. The expression heatmap of these genes is presented in Figure 4A. According to the expression profiles of the *GmMATE* genes across different treatments, the *GmMATE* genes were classified into nine clusters with time series analysis (Figure 4B). We focused on clusters 1 and 9, which showed a tendency of low expression in the NC, AC, and LD treatments, and high expression in the HD treatment. The expression patterns of genes within clusters 1 and 9 are depicted in Figure 4C. Noteworthily, within the two clusters, *GmMATE13* and *GmMATE75* demonstrated significant upregulation, with fold changes of 6.5 and 245, respectively, in the HD treatment compared to the NC treatment.

### 2.5. Character of GmMATE13 and GmMATE75 Genes in TBS

The full-length coding sequences (CDS) of *GmMATE13* and *GmMATE75* were amplified using cDNA from TBS. Sequencing results indicated that the full-length CDS of *GmMATE13* and *GmMATE75* are 1503 bp and 1674 bp, respectively. The sequence analysis revealed that the molecular formula of GmMATE13 protein is C_2457_H_3941_N_607_O_682_S_20_, with a total of 7707 atoms. The molecular weight is 53.5 kD, and the isoelectric point (PI) is 7.72. The amino acid composition is characterized by a high proportion of leucine (13.6%) and proline (10.4%), and a low proportion of cysteine (0.4%). The instability index (31.05 < 40) indicates that GmMATE13 is a stable protein. The SOPMA software (https://npsa-prabi.ibcp.fr/cgi-bin/npsa_automat.pl?page=/NPSA/npsa_sopma.html, accessed on 6 December 2022) predicted the secondary structure of this protein, where α-helices account for 57.00%, extended chains account for 12.00%, β-turns account for 5.40%, and irregular coils account for 25.6%.

**Figure 5:**
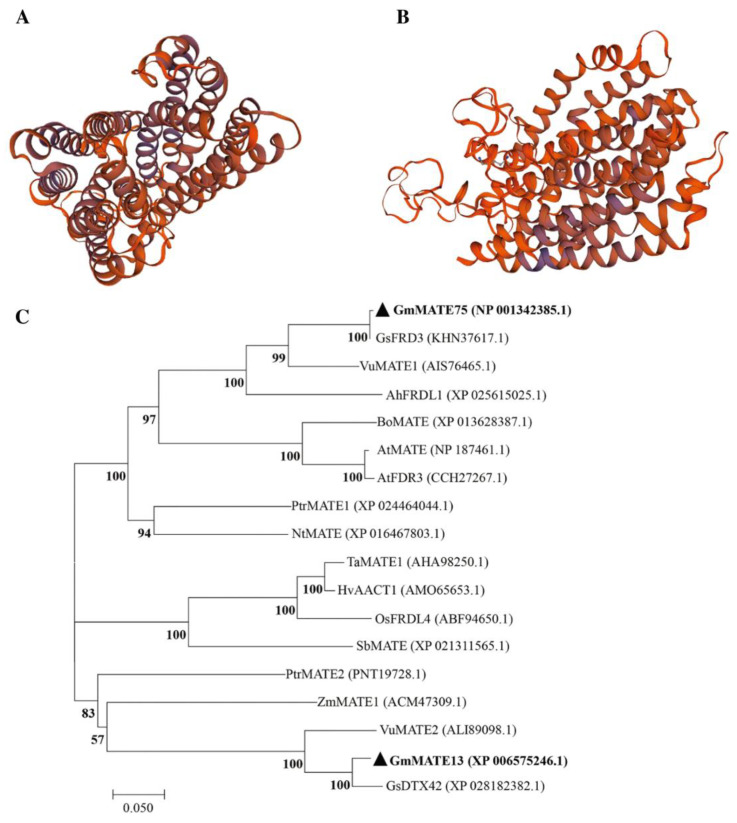
The structure and phylogenetic analysis of the GmMATE13 and GmMATE75 proteins in TBS. (**A**) Predicted 3D structure of GmMATE13. (**B**) Predicted 3D structure of GmMATE75. (**C**) Phylogenetic tree of GmMATE13 and GmMATE75 proteins.

The molecular formula of the GmMATE75 protein is C_2763_H_4426_N_702_O_760_S_23_, with a total of 8674 atoms. The molecular weight is 60.4 kD and the PI is 9.54. The amino acid composition of GmMATE75 includes a higher proportion of leucine (12.6%) and proline (11.1%), and a lower proportion of cysteine (0.7%). The instability index is 28.14 < 40, indicating that GmMATE75 is a stable protein. The SOPMA software (https://npsa-prabi.ibcp.fr/cgi-bin/npsa_automat.pl?page=/NPSA/npsa_sopma.html, accessed on 6 December 2022) predicted the secondary structure of this protein, where α-helices account for 53.14%, extended chains account for 11.85%, β-turns account for 4.67%, and irregular coils account for 30.34%. In addition, the SWISS-MODEL performed an online prediction of the tertiary structure of the GmMATE13 and GmMATE75 proteins (Figure 5). The amino acid sequences of GmMATE13 and GmMATE75 were compared using BlastP (https://blast.ncbi.nlm.nih.gov/Blast.cgi?PAGE=Proteins, accessed on 10 December 2022), and the sequences with higher homology were selected for constructing an evolutionary tree. The results show that GmMATE13 clusters with GsDTX42 and VuMATE2, while GmMATE75 clusters with GsFRD3, VuMATE1, and AhFDRL1 (Figure 5C).

### 2.6. The Expression of GmMATE13 and GmMATE75 upon Al^3+^ Stress

**Figure 6:**
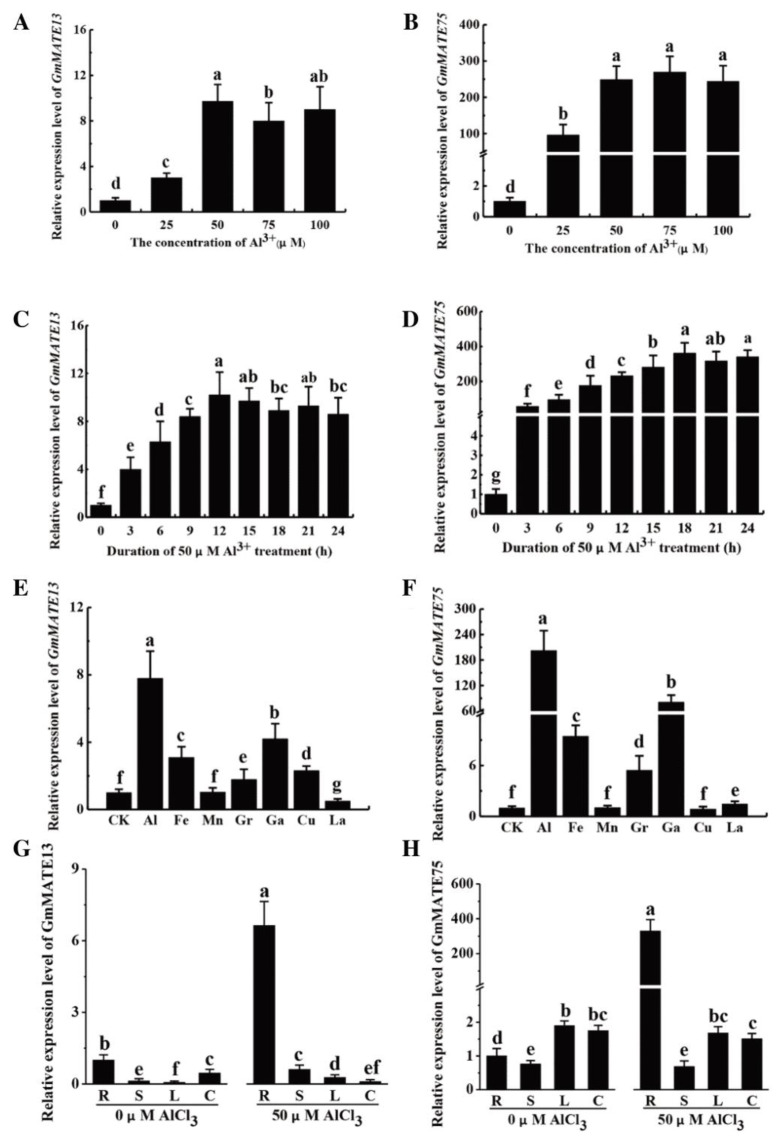
*GmMATE13* and *GmMATE75* expression pattern upon Al stress. (**A**,**B**) Relative expression of *GmMATE13* and *GmMATE75* treated with gradient AlCl_3_ concentration for 24 h. (**C**,**D**) Relative expression of *GmMATE13* and GmMATE75 treated with 50 µM AlCl_3_ at various times of exposure. (**E**,**F**) Expression level of *GmMATE13* and *GmMATE75* treated with different metal ions for 24 h. CK was the untreated group. (**G**,**H**) Expression pattern of *GmMATE13* and *GmMATE75* in different plant organs, including roots (R), stems (S), leaves (L), and cotyledons (C). Data shown as mean ± SEM of *n* = 3 independent experiments and was analyzed using one-way ANOVA with Dunnett’s multiple comparisons test. Different letters above columns indicate significance at *p* < 0.05.

The qRT-PCR results showed significant upregulation of both *GmMATE13* and *GmMATE75* under Al stress (Figure 6A,B). The two genes demonstrated significantly higher expression levels in response to high Al^3+^ concentration treatment (above 50 μM Al^3+^), as compared to their expression levels in low Al^3+^ concentration treatment (25 μM Al^3+^). Specifically, *GmMATE13* showed the highest expression level at 50 μM Al^3+^ treatment (Figure 6A), while *GmMATE75* showed the highest expression level at 75 μM Al^3+^ treatment (Figure 6B). In terms of treated time gradient of 0–24 h, both *GmMATE13* and *GmMATE75* expression increased with Al^3+^ treatment time. *GmMATE13* reached its highest expression level at 12 h (Figure 6C), while *GmMATE75* reached its highest expression level at 18 h (Figure 6D). Both *GmMATE13* and *GmMATE75* showed the highest expression level under Al treatment compared to other metal ion treatments. Additionally, *GmMATE13* was upregulated in response to Ga, Fe, Cu, and Gr treatments (Figure 6E), while *GmMATE75* was upregulated in response to Ga, Fe, and Gr treatments (Figure 6F). Furthermore, under Al stress, *GmMATE13* was upregulated in the roots, stems, and leaves of the TBS, while it was downregulated in the cotyledons (Figure 6G). On the other hand, *GmMATE75* was only upregulated in the roots under Al stress, with no change in expression in other tissues (Figure 6H).

### 2.7. Subcellular Localization of MATE Proteins

**Figure 7:**
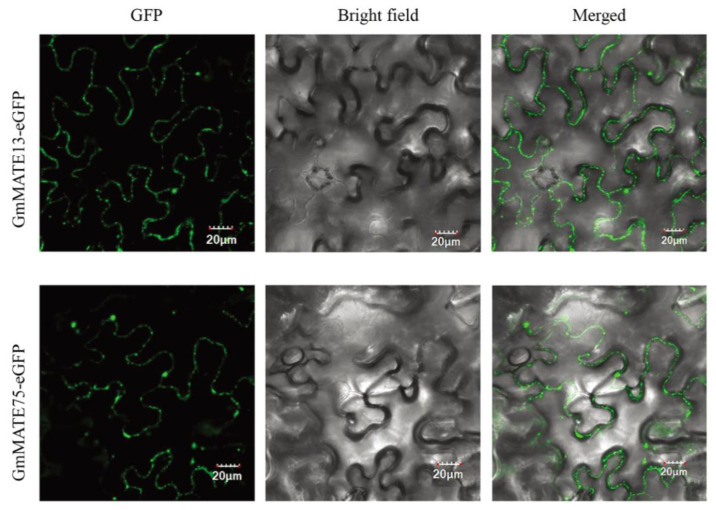
Subcellular localization of the GmMATE13 and GmMATE75 proteins; the images were captured with Zeiss LSM 900 confocal microscopy and the scale bar was 20 µm.

It has been reported that most MATE proteins in plants are localized on cell membranes [21]. After the transient expression of *GmMATE13*-eGFP and *GmMATE75*-eGFP plasmids in *Nicotiana benthamiana*, green fluorescence with confocal microscopy was found to be concentrated on the cytoplasmic membrane, indicating that both GmMATE13 and GmMATE75 proteins are localized on the plasma membrane of plant cells (Figure 7).

### 2.8. Screening and Al Tolerance Identification of Arabidopsis Mutant Complementation Plants

**Figure 8:**
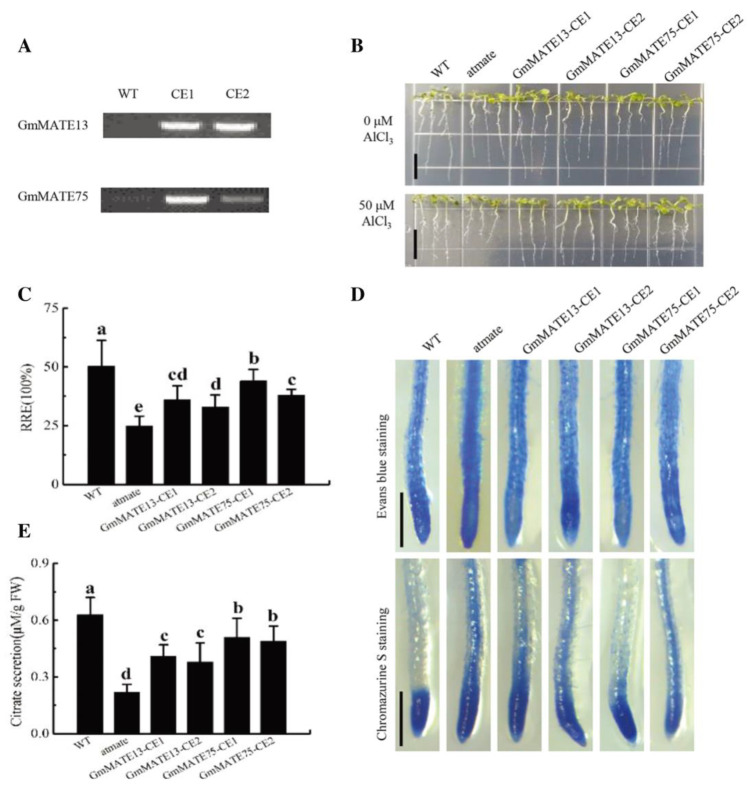
The function of *GmMATE13* and *GmMATE75* on Al resistance. (**A**) Expression of *GmMATE13* and *GmMATE75* in WT and two complementary lines (CE1 and CE2) were detected by RT-PCR. (**B**) Phenotypic analysis of WT, *atmate*, *GmMATE13*-CE, and *GmMATE75*-CE, scale bar was 1 cm. (**C**) Relative root elongation of the plants treated with 50 μM AlCl_3_ for 7 days. (**D**) Evans blue and Chrome Azurol S staining of the root tips treated with 50 μM AlCl_3_ for 24 h, scale bar was 0.5 mm. (**E**) Citrate secretion of the *Arabidopsis* roots treated with 50 AlCl_3_ Al for 24 h. Data shown as mean ± SEM of *n* = 4 independent experiments and was analyzed using one-way ANOVA with Dunnett’s multiple comparisons test. Different letters above columns indicate significance at *p* < 0.05.

RT-PCR analysis confirmed the complementary expression of *GmMATE13* and *GmMATE75* in the *Arabidopsis* mutant *atmate* (Figure 8A). The relative root elongation, root tip tissue staining, and citrate secretion were examined under Al stress. *Arabidopsis* complementation plants overexpressing *GmMATE13* or *GmMATE75* exhibited significantly higher relative root elongation compared to the *atmate* mutant but lower than the WT (Figure 8B,C). Staining with Evans blue and Chrome azurol S indicated reduced root tip damage and decreased absorption of Al^3+^ in the *Arabidopsis* complementation plants compared to the *atmate* mutant (Figure 8D). Citrate secretion under Al stress showed a similar pattern to root elongation, with significantly higher levels in *Arabidopsis* complementation plants overexpressing *GmMATE13* or *GmMATE75* compared to the *atmate* mutant (Figure 8E).

## 3. Discussion

Aluminum toxicity is one of the primary limiting factors for crop growth and yield in acidic soils. The secretion of organic acids, such as citrate, in the root system is a well-known mechanism for Al tolerance in plants [22]. Many studies have shown that the secretion of OAs is mediated by anion channels and transport proteins located on the plasma membrane. Multidrug and toxic compound extrusion transporters (MATE) are commonly found in plant cells, predominantly in cell membranes, where they serve a crucial function in expelling plant secondary metabolites and toxic compounds [23]. Over the past few decades, many members of the *MATE* gene family associated with aluminum tolerance have been identified, including the sorghum *SbMATE* gene [24], barley *HvAACT* gene [8], *Arabidopsis AtMATE* gene [25], maize *ZmMATE* gene [26], rice bean *VuMATE* gene [27], rice *OsFRDL4* gene [28], and peanut *AhFRDL1* gene [29]. These genes have been shown to play important roles in citrate secretion and genetic transformation of these genes can enhance the secretion of citrate in plant root tips, thereby alleviating aluminum toxicity.

MATE transporters have been found in both prokaryotic and eukaryotic organisms, exhibiting a distinctive topology [30]. However, the *MATE* family were conserved between dicots and monocots [19]. Referring to Duan et al. [21], Blast searches were conducted using the *MATE* genes from two extensively studied plants, *Arabidopsis thaliana* and *Oryza sativa*, as query sequences to identify *GmMATE* genes in the present study. In comparison to a previous study on *MATE* genes in soybeans that identified 117 *GmMATE* genes [16], our research has identified an expanded set of 124 *GmMATE* genes. The seven additional *GmMATE* genes, namely *GmMATE118-124*, are situated on chromosomes 5, 6, 8, 11, 12, 16, and 18, respectively, and they share MATE or MATE-like domains. The functional characterization of these genes awaits further investigation and could provide valuable insights in future studies.

The *GmMATE* genes were identified in the transcriptome profile of Tamba black soybeans to assess the relation of the expression of *GmMATE* genes and the Al tolerance of TBS. Among the *GmMATE* genes in TBS, *GmMATE13* and *GmMATE75* exhibited significant upregulation in response to high-dose Al^3+^ treatment, with fold changes of 6.5 and 245, respectively, compared to the NC treatment. They were proposed to function as important plasma-membrane-localized citrate transporters in TBS. Both genes were cloned and characterized, revealing a high degree of homology with GsFRD3, VuMATE1, VuMATE2, and GsDTX42. VuMATE1 and VuMATE2 are Al-activated citrate transporters that conferred Al-induced citrate efflux in *Arabidopsis* [31,32]. Additionally, subcellular localization showed that GmMATE13 and GmMATE75 were localized on the plasma membrane, consistent with the localization of EcMATE1 in *Eucalyptus camaldulensis* [33], OsFRDL4 in rice [28], and ZmMATE1 in maize [26], which verified their potential functioning as Al-activated citrate transporters. They were mainly expressed in plant roots upon Al^3+^ treatment. *GmMATE13* was upregulated in roots, stems, and leaves, while *GmMATE75* was only upregulated in roots, indicating the expression of *GmMATE13* participates in more pathways in addition to responses to Al stress and *GmMATE75* is closely related with the modulation of Al tolerance in roots. These findings are consistent with the *MATE* expression patterns in rice bean and buckwheat [32,34]. Furthermore, *GmMATE13* and *GmMATE75* showed the highest relative expression levels at 12 h and 18 h, respectively. Similarly, *PtrMATE1* was induced after 12 h of Al stress, while *PtrMATE2* was induced after 24 h in poplar. The different response times of the two *MATE* genes may suggest the synergistic secretion of citrate by these citrate channel proteins to adapt to Al stress [17]. These findings also imply the coordinated action of *GmMATE13* and *GmMATE75* in TBS to cope with Al stress.

Numerous studies have shown that overexpression of *MATE* genes can enhance citrate secretion in plants under Al stress and alleviate Al toxicity. For example, overexpression of the *BoMATE* and *GmMATE2* genes can increase citrate secretion in *Arabidopsis thaliana* and tobacco, respectively [35]. Additionally, in *Arabidopsis* mutants, overexpression of the *AhFRDL1* gene can restore citrate secretion and iron transport [29]. In the present study, transgenic complementation experiments in the *Arabidopsis* mutant *atmate* demonstrated that both the *GmMATE13* and *GmMATE75* genes mediated the secretion of citrate and improved Al tolerance. Furthermore, research by Liu et al. found that *GmMATE13* and *GmMATE75* are involved in response to Al stress [16]. Subsequently, Zhou et al. discovered that overexpressing *GmMATE75* in *Arabidopsis* resulted in increased citrate secretion under Al stress, thus alleviating the inhibition of root elongation caused by Al [19]. Wang et al. found that the overexpression of *GmMATE13* significantly increased citrate secretion in soybean hairy roots [36]. These results further demonstrate the involvement of *GmMATE13* and *GmMATE75* in citrate secretion. In summary, our results indicated both *GmMATE13* and *GmMATE75* are citrate transporter proteins located on the cytoplasmic membrane. Overexpression of both genes in the mutant *Arabidopsis* can alleviate Al-stress-induced root tip impairment. This discovery provides valuable gene resources for breeding plant varieties suitable for growth in acidic soils.

In summary, GmMATE13 or GmMATE75 are proposed as key participants in conferring aluminum tolerance in TBS. They are citrate transporters at the plasma membrane. The expression levels of *GmMATE13* and *GmMATE75* are significantly upregulated under Al stress, which contribute to inducing the production of citrate efflux. The tolerance of plants to aluminum toxicity could therefore increase. The two *GmMATE* genes show differential expression responses under different durations of aluminum stress, indicating potential synergistic actions of their encoded proteins in adapting to aluminum stress.

## 4. Materials and Methods

### 4.1. Identification of MATE Genes in Soybean and Their Molecular Characteristics

The genome sequences and the annotation file of soybean (*Glycine max* Wm82.a2.v1) were downloaded from Phytozome (https://phytozome-next.jgi.doe.gov/, accessed on 20 December 2023). The 56 and 45 *MATE* gene family members in *Arabidopsis thaliana* and *Oryza sativa* were used as seed sequences to search the genome of soybeans for MATE-related protein sequences with the threshold E-value setting to ≤1 × 10^−5^ [21]. The obtained sequences were checked in the NCBI Conserved Domain Search (https://www.ncbi.nlm.nih.gov/Structure/cdd/wrpsb.cgi, accessed on 12 January 2024) and Pfam (http://pfam.xfam.org/, accessed on 12 January 2024) to detect the existence of conserved MATE protein domains. The protein length (number of amino acids), molecular weight, and theoretical isoelectric point were computed by ExPASy (https://web.expasy.org/protparam/, accessed on 20 January 2024) [37].

### 4.2. Phylogenetic Analysis of MATE Gene Family

The phylogenetic tree of protein sequences encoded by *MATE* family genes in *A. thaliana*, *O. sativa*, and *G. max* was constructed in the MEGA 6.0 software (Koichiro Tamura, Japan) with the maximum likelihood (ML) algorithm under 1000 bootstrap tests. The protein sequence used in constructing the phylogenetic tree is listed in Table S3.

### 4.3. Gene Structure, Motif Analysis, and Chromosomal Location

Conserved motifs within the MATE proteins were identified using the Multiple Em for Motif Elicitation (MEME, https://meme-suite.org/, accessed on 12 January 2023). The maximum number of motifs were set to ten. The gene structure and chromosome location of each *GmMATE* gene was analyzed and illustrated in TBtools (Guangzhou, China) with the annotation file of *G. max* Wm82.a2.v1.

### 4.4. Plant Culture and Al^3+^ Treatments

TBS seeds were sterilized, rinsed, and then incubated on moistened filter paper in the dark at 25 °C for germination. The seedlings were cultured in 8 L aquariums with 1/2 Hoagland’s nutrient solution (pH 6.0), with a light cycle of 14 h light/10 h dark (200 µmol photons m^−2^·s^−1^) at a temperature of 27/22 °C (day/night). The nutrient solution was refreshed every two days. After the true leaf was fully expanded, the seedlings were transferred into 0.5 mM CaCl_2_ solution (pH 4.3) pretreated for 24 h. Then, the seedlings were transferred and cultured in the solution with 10 and 50 μM AlCl_3_ (pH 4.3, 0.5 mmol/L CaCl_2_) for 3 d. After treatment, root apices (0–2 cm) were excised and immediately frozen in liquid nitrogen before isolating the total RNA. Each treatment was replicated three times. The seedlings without CaCl_2_ and AlCl_3_ treatments and those only treated with CaCl_2_ were used as the neutral and acidic controls.

*Arabidopsis* wild type and Al sensitivity mutant *atmate* were utilized for transfection *GmMATE* genes and evaluating the response of *GmMATE* genes to Al stress. *Arabidopsis* seeds were placed on 1/2 Murashige and Skoog (MS) agar medium and kept in darkness at 4 °C for 2 days. Subsequently, the *Arabidopsis* seedlings were transferred to fresh 1/2 MS medium containing a specific concentration of Al and cultured for several days at 22 °C under long-day conditions until all the samples were collected. The collected plant materials were instantly put into liquid nitrogen and stored at −80 °C for RNA isolation.

### 4.5. RNA Extraction and Transcriptome Sequencing

Total RNA was extracted using the RNAi^so^ Plus kit (Takara, Dalian, China) according to the manufacturer’s description and the concentration and integrity of RNA was measured using a NanoDrop 2000 spectrophotometer (Thermo, Waltham, MA, USA) and the 2100 bioanalyzer (Agilent Technologies, Santa Clara, CA, USA), respectively. Samples with high RNA integrity and OD260/280 (above eight) were selected for library construction [38]. The whole library was finally sequenced with the Illumina HiSeq platform (Novogene, Beijing, China).

### 4.6. Detecting MATE Family Gene Expression via RNA-Seq and Cluster Analysis

The transcription sequence of *GmMATE* genes were used in discovering *MATE* gene expression in TBS. Gene expression levels were estimated by fragments per kilobase of transcript per million fragments (FPKM) mapped. The expression level of *MATE* family genes was evaluated among different treatment groups, namely neutral control (NC), acidic control (AC), low dose (LD), and high dose (HD) of AlCl_3_ (10 and 50 μmol/L).

The Mfuzz R package (version 2.62) was employed to cluster the expression levels of all *MATE* genes. This package is based on Fuzzy C-Means Clustering, which is initially used to analyze the time trend of gene expression in data with time series characteristics [39]. This cluster analysis was performed on the transcriptome data to comprehend the dynamic expression patterns of the biological molecules with the associated functions. C-means clustering was calculated using the reads per million (FPKM) fragments of the neutral and acidic controls and two concentrations of Al^3+^ treatment.

### 4.7. Character of the GmMATE13 and GmMATE75 Genes

The physicochemical properties of the GmMATE13 and GmMATE75 proteins were predicted using ExPASY ProtParam (https://web.expasy.org/protparam/, accessed on 10 December 2022). Protein function domain, secondary structure, and transmembrane domain prediction were carried out using the CDD (https://www.ncbi.nlmnih.gov/structure/cdd/wrpsb.cgi, accessed on 10 December 2022), SOPMA (https://npsa-prabi.ibcp.fr/cgi-bin/npsa_automat.pl?page=npsa_sopma.html, accessed on 10 December 2022), and TMHMM (http://www.cbs.dtu.dk/services/TMHMM/, accessed on 10 December 2022) online software, respectively. The tertiary structures of GmMATE13 and GmMATE75 were predicted using SWISS-MODEL (https://swissmodel.expasy.org/interactive, accessed on 15 December 2022). Additionally, homologous sequences of GmMATE13 and GmMATE75 were downloaded after aligning the amino acid sequences with BLASTP, and a phylogenetic tree was constructed using the MEGA6.0 software.

### 4.8. GmMATE13 and GmMATE75 Expression

After 2 weeks of cultivation, TBS seedlings were pre-treated with 0.5 mmol/L CaCl_2_ (pH 4.5) for 24 h. To test the sensitivity of *MATE* genes to Al^3+^ concentration, the seedlings were transferred and cultured in the solution with gradient concentrations of 0, 25, 50, 75, and 100 μM AlCl_3_ (pH 4.3, 0.5 mmol/L CaCl_2_) for 24 h. Root tips were collected to determine the expression level of *MATE* genes. Moreover, to analyze the temporal expression pattern of MATE genes in response to Al stress, the seedlings were transferred to a solution of 50 μmol/L AlCl_3_ (pH 4.3, 0.5 mmol/L CaCl_2_) for 24 h, and the root tips were obtained from different time points at 3, 6, 9, 12, 15, 18, 21, and 24 h. To determine the impact of other metal ions on *GmMATE* gene expression, the seedlings were transferred to solutions containing various metal ions, including 50 μmol/L AlCl_3_, 5 μmol/L CuCl_2_, 50 μmol/L FeCl_3_, 50 μmol/L Ga(NO_3_)_3_, 50 μmol/L GrCl_3_, 50 μM La(NO_3_)_3_, and 50 μmol/L MnCl_2_ cultured for 24 h, respectively. Root tips were collected for measuring the expression of *GmMATE 13* and *GmMATE 75*. Three biological replicates were performed for each treatment. Furthermore, to evaluate the organ-specific expression patterns of *MATE* genes, different parts of TBS seedlings, including root tips (R), stems (S), leaves (L), and cotyledons (C) were collected after treatment with 50 μM AlCl_3_ solution for 24 h. Three biological replicates were performed for each treatment.

Total RNA extraction was the same as described above. Complementary DNA (cDNA) was synthesized using the PrimeScript™ II 1st Strand cDNA Synthesis Kit (Takara, Dalian, Japan) following the manufacturer’s instructions. Real-time quantitative PCR was performed following the manufacturer’s instructions. The primers designed for *GmMATE13* and *GmMATE75* were based on their coding regions. Real-time PCR was performed to amplify the PCR products of the *GmMATE13* and *GmMATE75* PCR products. The primers used are listed in Table S4. The mRNA abundance was calculated according to the 2^−ΔΔCt^ method, with the expression levels normalized to the internal reference gene 40S rRNA gene (XM_0035498336.4).

### 4.9. Subcellular Localization of GmMATE Genes

To determine the subcellular localization of GmMATE13 and GmMATE75 proteins, their coding sequences (CDS) were cloned into the pBI121-eGFP vector with a CaMV 35S promoter. The GFP control and *GmMATE* expression vectors were transiently transformed into *Nicotiana benthamiana* with the *Agrobacterium*-mediated transient expression system. Confocal microscopy (Zeiss, LSM 900, Jena, Germany) was used to capture GFP fluorescence and bright-field images, confirming the subcellular location of GmMATE proteins.

### 4.10. Heterologous Expression of GmMATEs in the Arabidopsis Al Sensitivity Mutant Atmate

The *Arabidopsis* Al sensitivity mutant *atmate* was used to create GmMATE transgenic lines expressing *GmMATE13* and *GmMATE75*. The CDS of the genes were cloned into a pCXSN vector with a CaMV 35S promoter. Positive colonies were identified using colony PCR and Sanger sequencing. *Agrobacterium* strain K599 was used to transfer the expression vectors into *atmate*. Two independent transgenic lines for each gene were obtained and named GmMATE13-CE1, GmMATE13-CE2, GmMATE75-CE1, and GmMATE75-CE2.

### 4.11. Al Resistance Analysis in Transgenic Atmate

The transgenic *atmate* lines, along with wild type (WT) and *atmate* plants, were tested for aluminum (Al) resistance. Two-week-old seedlings were pre-treated with 0.5 mM CaCl_2_ and then exposed to 0 (control) and 50 μM AlCl_3_ for 7 days. Root tips were collected for analysis of relative root elongation (RRE), Evans blue staining, and Chrome Azurol S staining. RRE% = (Al-treated root length − Al untreated root length)/(Control untreated root length − Control treated root length). Evans blue staining was used to assess plant cell injury and activity under acidic conditions. Root tips were stained with a 0.25% Evans blue solution. Chrome azurol S staining was performed to measure the accumulation of Al. Root tips were stained with a 0.1% Chrome azurol S solution. Stained roots were observed and captured using a dissecting microscope.

### 4.12. Statistical Analysis

Statistical analyses were performed using SPSS 22.0. One-way ANOVA and Student’s *t*-test were used to compare significant differences among different groups, with *p* < 0.05 considered statistically significant. Results were presented as mean ± SEM (standard error of the mean).

## 5. Conclusions

The expression patterns of *MATE* genes in Tamba black soybean exhibit varied responses to aluminum stress. Among them, two *MATE* genes, *GsMATE13* and *GsMATE75*, were found to be significantly upregulated under aluminum stress. Based on the results of protein structure, phylogenetic analysis, gene expression, protein localization, and overexpression validation in *Arabidopsis*, these two proteins were identified as citrate transporters located on the plasma membrane, participating in the efflux of citrate ions and alleviating aluminum toxicity in plants. This may be one of the reasons for aluminum tolerance in Tamba black soybean plants. Subsequent studies will involve knockout and overexpression of the *MATE* genes in Tamba black soybean to determine their roles in aluminum tolerance.

## Data Availability

Data is contained within the article or Supplementary Materials.

## Supplementary Materials

The following supporting information can be downloaded at: https://www.mdpi.com/article/10.3390/ijms25073711/s1.
