# Supplementary material for: Identification of MATE Family and Characterization of GmMATE13 and GmMATE75 in Soybean’s Response to Aluminum Stress
Source: Int J Mol Sci. 2024 Mar 26;25(7):3711. doi: 10.3390/ijms25073711 (PMC11011378; doi:10.3390/ijms25073711)
Supplement: Supplementary file 1 [file ijms-25-03711-s001.zip › ijms-2918126-supplementary.pdf]

Table S1. Characters of the GmMATE proteins.

Gene name	JGI ID	Protein length (aa)	Molecular weight(Da)	Isoelectric point
GmMATE1	Glyma.01G007100	343	36805.41	7.48
GmMATE2	Glyma.01G025200	491	54297.15	5.93
GmMATE3	Glyma.01G026200	518	56511.29	5.77
GmMATE4	Glyma.01G132200	538	58666.11	6.96
GmMATE5	Glyma.01G213000	511	55508.20	8.23
GmMATE6	Glyma.01G216600	541	58998.70	8.06
GmMATE7	Glyma.02G038600	507	55501.60	6.73
GmMATE8	Glyma.02G039500	492	54733.67	7.57
GmMATE9	Glyma.02G075300	507	54832.32	8.50
GmMATE10	Glyma.02G089900	476	51784.54	7.01
GmMATE11	Glyma.02G090000	482	52143.68	5.59
GmMATE12	Glyma.02G090100	475	51329.79	8.98
GmMATE13	Glyma.02G181800	500	53538.18	7.72
GmMATE14	Glyma.02G218400	527	56999.57	8.62
GmMATE15	Glyma.02G310800	526	56643.60	9.70
GmMATE16	Glyma.03G005200	488	53896.75	8.03
GmMATE17	Glyma.03G005300	487	53908.84	6.45
GmMATE18	Glyma.03G005400	487	53628.32	5.40
GmMATE19	Glyma.03G005500	429	47141.11	8.87
GmMATE20	Glyma.03G005600	490	54084.85	6.90
GmMATE21	Glyma.03G005800	494	54597.63	7.50
GmMATE22	Glyma.03G036200	539	58602.06	7.43
GmMATE23	Glyma.04G089000	537	58756.28	8.80
GmMATE24	Glyma.04G097700	524	57266.61	8.01
GmMATE25	Glyma.04G097900	503	55390.87	5.29
GmMATE26	Glyma.04G102100	199	22101.34	8.42
GmMATE27	Glyma.05G001700	486	52789.16	6.55
GmMATE28	Glyma.05G049700	531	57947.35	7.44
GmMATE29	Glyma.05G054000	525	57134.66	8.93
GmMATE30	Glyma.05G054100	315	34689.82	9.43
GmMATE31	Glyma.05G226400	480	51863.58	8.14
GmMATE32	Glyma.06G091100	548	59505.15	7.51
GmMATE33	Glyma.06G103400	480	52566.46	7.86
GmMATE34	Glyma.06G305200	517	56027.78	6.19
GmMATE35	Glyma.06G318700	480	51993.42	9.03
GmMATE36	Glyma.07G099900	469	51260.00	9.27
GmMATE37	Glyma.07G100000	467	50905.44	9.15
GmMATE38	Glyma.07G100100	422	45942.72	8.46
GmMATE39	Glyma.07G124600	497	53882.29	6.59
GmMATE40	Glyma.07G156100	539	57276.57	6.24

GmMATE41	Glyma.07G246100	506	55485.71	8.64
GmMATE42	Glyma.08G033200	494	53145.83	6.59
GmMATE43	Glyma.08G050500	498	54695.73	8.59
GmMATE44	Glyma.08G050700	475	52207.37	9.32
GmMATE45	Glyma.08G244400	173	19261.97	7.58
GmMATE46	Glyma.09G042700	528	57747.13	8.76
GmMATE47	Glyma.09G102800	556	59867.47	9.43
GmMATE48	Glyma.09G134000	522	57574.19	6.98
GmMATE49	Glyma.09G134100	488	54083.16	8.22
GmMATE50	Glyma.09G134200	488	54192.10	7.91
GmMATE51	Glyma.09G146800	572	62436.51	8.64
GmMATE52	Glyma.09G178700	323	35443.88	9.31
GmMATE53	Glyma.09G178800	444	48774.14	9.16
GmMATE54	Glyma.09G178900	474	51503.57	9.23
GmMATE55	Glyma.09G179000	489	53142.64	8.41
GmMATE56	Glyma.09G257400	510	55533.58	5.36
GmMATE57	Glyma.09G276100	542	59162.51	7.85
GmMATE58	Glyma.10G099900	501	53844.53	6.23
GmMATE59	Glyma.10G231800	80	8712.35	6.50
GmMATE60	Glyma.10G232200	494	54060.18	5.65
GmMATE61	Glyma.10G239000	530	57916.80	8.21
GmMATE62	Glyma.10G267300	478	52266.96	6.32
GmMATE63	Glyma.10G267400	477	52176.96	6.65
GmMATE64	Glyma.10G267600	146	16023.96	8.57
GmMATE65	Glyma.10G267700	475	51702.26	7.49
GmMATE66	Glyma.10G267800	459	49978.14	7.07
GmMATE67	Glyma.11G026300	534	58040.87	8.68
GmMATE68	Glyma.11G028900	467	50963.91	8.15
GmMATE69	Glyma.11G112100	548	59820.58	8.90
GmMATE70	Glyma.11G112200	546	59513.21	9.18
GmMATE71	Glyma.12G099300	441	48129.84	6.65
GmMATE72	Glyma.12G194000	504	54372.93	5.90
GmMATE73	Glyma.12G225600	287	31508.89	9.07
GmMATE74	Glyma.12G237400	549	69698.46	9.40
GmMATE75	Glyma.13G203000	428	46528.63	9.36
GmMATE76	Glyma.13G275400	504	55564.59	8.28
GmMATE77	Glyma.13G275500	500	55105.07	7.16
GmMATE78	Glyma.13G275600	536	59475.90	6.17
GmMATE79	Glyma.13G339800	555	59390.55	6.76
GmMATE80	Glyma.14G002000	559	60438.80	9.60
GmMATE81	Glyma.14G032400	505	55204.03	8.10
GmMATE82	Glyma.14G078000	483	52446.27	8.10
GmMATE83	Glyma.14G158100	129	14551.38	8.18
GmMATE84	Glyma.15G034600	554	59382.86	7.66

GmMATE85	Glyma.15G107100	505	54762.52	7.09
GmMATE86	Glyma.15G150300	516	56369.59	8.62
GmMATE87	Glyma.15G274600	553	59165.46	9.46
GmMATE88	Glyma.16G149100	261	29192.42	7.91
GmMATE89	Glyma.16G157300	505	54515.81	8.39
GmMATE90	Glyma.16G180000	477	52924.83	5.98
GmMATE91	Glyma.16G180200	488	54132.13	6.64
GmMATE92	Glyma.16G198700	562	60875.93	8.63
GmMATE93	Glyma.17G027900	507	55484.66	8.39
GmMATE94	Glyma.17G131700	511	55550.12	8.48
GmMATE95	Glyma.17G136400	505	55034.06	8.91
GmMATE96	Glyma.17G136500	507	55266.87	9.26
GmMATE97	Glyma.17G175100	355	38042.08	9.12
GmMATE98	Glyma.17G247400	483	52470.19	6.62
GmMATE99	Glyma.18G110800	112	12473.05	5.38
GmMATE100	Glyma.18G143700	509	55625.41	6.30
GmMATE101	Glyma.18G206500	593	64280.52	5.83
GmMATE102	Glyma.18G214800	505	55348.60	8.52
GmMATE103	Glyma.18G235200	505	55266.15	5.60
GmMATE104	Glyma.18G293100	480	51993.52	9.07
GmMATE105	Glyma.18G293200	406	43894.12	6.04
GmMATE106	Glyma.18G293300	288	41861.79	8.04
GmMATE107	Glyma.19G001600	498	53804.16	6.32
GmMATE108	Glyma.19G120200	495	54624.36	7.60
GmMATE109	Glyma.19G120300	496	54692.57	8.04
GmMATE110	Glyma.19G120700	400	44084.40	6.72
GmMATE111	Glyma.19G120900	491	54183.95	6.16
GmMATE112	Glyma.19G244000	273	29530.09	9.27
GmMATE113	Glyma.20G123300	555	60571.17	7.40
GmMATE114	Glyma.20G123400	409	44326.97	5.13
GmMATE115	Glyma.20G123500	476	52024.51	6.65
GmMATE116	Glyma.20G155500	550	60448.62	8.30
GmMATE117	Glyma.20G162000	494	54051.22	5.78
GmMATE118	Glyma.05G243100	373	41788.58	9.42
GmMATE119	Glyma.06G099600	102	11263.01	7.74
GmMATE120	Glyma.08G282600	351	38073.32	5.96
GmMATE121	Glyma.11G133900	125	13457.96	6.00
GmMATE122	Glyma.12G122000	208	22412.43	9.15
GmMATE123	Glyma.16G180100	235	26168.82	8.62
GmMATE124	Glyma.18G097600	494	54051.22	5.78

Table S2. Conserved sequence detected in GmMATE.

**Motif number**	**Amino acid sequence**
1	TLCGQAYGAKZYHMLGVYLQR

2	AJPSAVMVCLEWWYFEILVLLAGLLPNPK
3	JWJFMEPILLLLGQDPEIAEVAGLYAJWLIPALFAYAV
4	GVARGSGWQKJGAYVNLGCYY
5	SAAASTRVSNELGAGNPKAAK
6	YLLSVISTAFVGHLGELELAA
7	FPLQRFLQTQSITLP
8	KKTWTGFSFEAFKGJWEFFKL
9	AISFANVTGFSVLSGLAGGLE
10	LRHVWGYIFTNDKEVVD

Table S3. MATE protein sequence used in constructing the phylogenetic tree.

ID	Sequence
AT1G11670	MGSEATTAVNNLQQPLLESTKSEADFRMESVLTDTHLSYFRRIYLASLIEMKYLF<br>HLAAPAIFVYVINNGMSMLTRIFAGRLGSMQLAAASLGNSGFNMFTLGLMLG<br>MGSAVETLCGQAHGAHRYDMLGVYLQRSTIVLVITGLPMTLLFIFSKPLLISLGE<br>PADVASVASVFVYGMIPMIFAYAVNFPIQKFLQSQSIVTPSAYISAATLVIHLILS<br>WLSVFKFGWGLLGLSVVHSLSWWIIVLAQIIYIKISPRCRRTWDGFSWKAFDGL<br>WDFFQLSAASAVMLCLESWYSQILVLLAGLLKDPELALDSLAICMSISAMSFMV<br>SVGFNAAASVRVSNELGAGNPRSAAFSTAVTTGVSFLLSLFEAIVILSWRHVISYI<br>FTDSPAVAEAVAELSPFLAITIVLNGVQPVLSGVAVGCGWQAYVAYVNIGCYYI<br>VGIPIGYVLGFTYDMGARGIWTGMIGGTLMQTIILVIVTFRTDWDKEVEKASRRL<br>DQWEDTSPLLKQ
AT1G12950	MEKDNDFKDPFLASTEEEELDPATQKALMEYLGVGSRASSLVSFSSTAVDIPPIS<br>GVGDFVREFRIESRKLWKLAGPAIFTTMSQYSLGAVTQVFAGHISTLALAAVSIE<br>NSVIAGFSFGIMLGMGSALETLCGQAFGAGKVSMLGVYLQRSWVILSVTALFLS<br>LIYIFAAPILTFIGQTAAISAMAGIFSIYMIPQIFAYAINFPTAKFLQSQSKIMVMA<br>GISGVVLVIHSFFTWLVMSRLHWGLPGLALVLNTSWWVIVVAQLVYIFNCTCG<br>EAWSGFTWEAFHNLWGFVKLSLASAAMLCLEIWYFMALVLFAGYLKNAEVSV<br>AALSICMNILGWAAMVAFGTNAAVSVRVSNELGASHPRTAKFSLVVAVILSTAI<br>GMFIAAGLLFFRNEYPVLFVEDEEVRNVVRELTPMLAFCIVINNVQPVLSGVAV<br>GAGWQAVVAYVNIACYYLFGVPFGLLLGFKLEYGVMGIWWGMVTGTFVQSIV<br>LTWMICKTNWEKEASMAEERIKEWGGVPAEKETLLN
AT1G15150	MQDAERTTNDPVDRIEKVTWRDLQDGSFTAELKRLICFAAPMAAVVIIQFMIQI<br>ISMVMVGHLGRLSLASASFAVSFCNVTGFSFIIGLSCALDTLSGQAYGAKLYRKL<br>GVQAYTAMFCLTLVCLPLSLLWFNMGKLIVILGQDPAIAHEAGRYAAWLIPGL<br>FAYAVLQPLIRYFKNQSLITPLLVTSSVVFCIHVPLCWLLVYKSGLGHIGGALALS<br>LSYWLYAIFLGSFMYYSSACSETRAPLTMEIFEGVREFIKYALPSAAMLCLEWWS<br>YELIILLSGLLPNPQLETSVLSICFETLSITYSIPLAIAAAASTRISNELGAGNSRAA<br>HIVVYAAMSLAVMDALMVSMSLLAGRHVFGHVFSSDKKTIEYVAKMAPLVSIS<br>IILDSLQGVLSGVASGCGWQHIGAYINFGAFYLWGIPIAASLAFWVHLKGVGL<br>WIGILAGAVLQTLLLALVTGCTNWKTQAREARERMAVAHESELTESELPI
AT1G15160	MEDAESTTKDPVDRVEKVTWRDLQDGSFTAELKKLICFAAPMAAVVITQSMLQ<br>IITMVIVGHLGRLSLASASFAISFCNVTGFSFIMGLSCALDTLSGQAYGAKLYRKL<br>GVQAYTAMFCLTLVCLPLSLLWFNMGKLLVILGQDPSIAHEAGRFAAWLIPGL

	FAYAVLQPLTRYFKNQSLITPLLITSCVVFCLHVPLCWLLVYKSGLDHIGGALALSLSYWLYAIFLGSFMYFSSACSETRAPLTMEIFEGVREFIKYALPSAAMLCLEWWSYELIILLSGLLPNPQLETSVLSVCLQTLSMTYSIPLAIAAAASTRISNELGAGNSRAAHIVVYAAMSLAVVDALMVGTSLLAGKNLLGQVFSSDKNTIDYVAKMAPLVSISLILDSLQGVLSGVASGCGWQHIGAYINFGAFYLWGIPIAASLAFWVHLKGVGLWIGIIAGAVLQTLLLALVTGCINWENQAREARKRMAVAHESELTESELPF
AT1G15170	MGDAESTKDRLLLPVERVENVTWSDLRDGSFTVELKRLIFFAAPMAAVVIAQFMLQIVSMMMVGHLGNLSLASASLASSFCNVTGFSFIIGLSCALDTLSGQAYGAKLYRKLGVQTYTAMFCLALVCLPLSLIWFNMEKLLLILGQDPSIAHEAGKYATWLIPGLFAYAVLQPLTRYFQNQSLITPLLITSYVVFCIHVPLCWFLVYNSGLGNLGGALAISLSNWLYAIFLGSFMYYSSACSETRAPLSMEIFDGIGEFFKYALPSAAMICLEWWSYELIILLSGLLPNPQLETSVLSVCLQTISTMYSIPLAIAAAASTRISNELGAGNSRAAHIVVYAAMSLAVIDALIVSMSLLIGRNLFGHIFSSDKETIDYVAKMAPLVSISLMLDALQGVLSGIARGCGWQHIGAYINLGAFYLWGIPIAASLAFWIHLKGVGLWIGIQAGAVLQTLLLALVTGCTNWESQADKARNRMALAYGT
AT1G15180	MGDAESTSKTSLLLPVERVENVTWRDLRDGLFTAELKRLICFAAPMAAVVIAQFMLQIISMVMVGHLGNLSLASASLASSFCNVTGFSFIVGLSCALDTLSGQAYGAKLYRKVGVQTYTAMFCLALVCLPLTLIWLNMETLLVFLGQDPSIAHEAGRYAACLIPGLFAYAVLQPLTRYFQNQSMITPLLITSCFVFCLHVPLCWLLVYKSGLGNLGGALALSFSNCLYTIILGSLMCFSSACSETRAPLSMEIFDGIGEFFRYALPSAAMICLEWWSYELIILLSGLLPNPQLETSVLSVCLQTTATVYSIHLAIAAAASTRISNELGAGNSRAANIVVYAAMSLAVVEILILSTSLLVGRNVFGHVFSSDKETIDYVAKMAPLVSISLILDGLQGVLSGIARGCGWQHIGAYINLGAFYLWGIPIAASLAFWIHLKGVGLWIGIQAGAVLQTLLLTLVTGCTNWESQADKARNRMALAYGT
AT1G23300	METLNVDHEDTISSEQEHRAHTKSDTDMPPISGGRDFIRQFAAESKKLWWLAGPAIFTSFCQYSLGAVTQILAGHVNTLALAAVSIQNSVISGFSVGIMLGMGSALATLCGQAYGAGQLEMMGIYLQRSWIILNSCALLLCLFYVFATPLLSLLGQSPEISKAAGKFSLWMIPQLFAYAVNFATAKFLQAQSKVIAMAVIAATVLLQHTLLSWLLMLKLRWGMAGGAVVLNMSWWLIDVTQIVYICGGSSGRAWSGLSWMAFKNLRGFARLSLASAVMVCLEVWYFMALILFAGYLKNPQVSVAALSICMNILGWPIMVAFGFNAAVSVRESNELGAEHPRRAKFLLIVAMITSVSIGIVISVTLIVLRDKYPAMFSDDEEVRVLVKQLTPLLALTIVINNIQPVLSGVAVGAGWQGIVAYVNIGCYYLCGIPIGLVLGYKMELGVKGIWTGMLTGTVVQTSVLLFIIYRTNWKKEASLAEARIKKWGDQSNKREEIDLCEEDENNSNGENNHRK
AT1G33080	MARREGEVTETLLKKSTENRGEDRDGLGMKEKVWRESKKLWVVAGPAIFTRFSTSGLSLISQAFIGHLGSTELAAYSITLTVLLRFSNGILLGMASALETLCGQAYGAKQYHMLGIYLQRSWIVLTGCTICLMPIYIFAGPILLALGQEERLVRVARIIALWVIGINISFVPSFTCQMFLQAQSKNKIIAYVAAVSLGVHVFLSWLLVVHFDFGIAGAMTSSLVAHWLPNIAQVLFVTCGGCTETWRGFSWLAFKDLWPVFKLSVSSGGMICLELWYNSILILLTGNLKNAEVALNALAICININALEMMVAFGFMAAASVRVSNEIGSGNSNGAKFATMVVVSTSLSIGIIFFFIFLFLRERVSYIFTTSEAVATQVADLSPLLAFSILLNSIQPVLSGVAVGAGWQKYVTVVNLACYYLVGIPSGLFLGYVVGLQVKGVWLGMIFGIFVQTCVLTVMTMRTDWDQQVSSSLKRLNRWVEPESPSRNQTLQNE

AT1G33090	MAGEGGELTAALLKKTTENGGEENDELGLKEKVWIESKKLWVVAAPSIFTKFS<br>TYGVSLVTQGFVGHIGPTELAAYSITFTVLLRFSNGILLGMASALGTLCGQAYGA<br>KQYHMLGIHLQRSWIVLTGCTICIMPIFIFSGPILLALGQEDHIVRVARVIALWLI<br>AINFTFVPAFTCQIFLQSQSKNKIIAYVSAVTLGLHVFFSWLLVVHFNFGITGAM<br>TSTLVAFWMPNIVQLLYVTSGGCKDTWRGFTMLAFKDLWPVFKLSLSSGGMV<br>CLELWYNSILVLLTGNLKNAEVAIDALAICINVNALQMMIALGFLAAVSVRVS<br>NELGRGNPEGAKFATIVAVFTSLSIGLVLFFVFLFLRGRISYIFTTSEAVAAEVADL<br>SPLLAFSILLNSVQPVLSGVAVGAGWQGYVAYINLACYYLLGIPVGLVLGYVVG<br>LQVKGVWIGMLFGIFVQTCVLTIMTLRTDWDQQVSTSLKNINRWVVPESRDAN<br>QISSEE
AT1G33100	MAGRGGELTEALVKKTGREEEDELGMKEKVWIESKKLWVVAAPAIFTRYSTFG<br>VSMVTQAFIGHLGPTELAAYSITFTILLRFSNGILLGMAGALGTLCGQAYGAKQ<br>YQMLGIYLQRSWIVLTGGTICLMPVFIFAGPILLALGQEERIVRVARVLALWVIGI<br>NFSFVPSFTCQMFLQAQSKNKIISYVTAVSLGLHVFFSWLLVAHFNFGITGAMTS<br>MLIAFWLPIIVQLLYVTCGGCKDTWRGFSMLAFKDLWPVLKLSLSSGGMLCLEL<br>WYNSVLVLLTGNLKNAEVALDALAICISINALEMMIALGFLAAVSVRVSNELGS<br>GNPKGAKFATLIAVFTSLSIGIVLFFVFLFLRGRISYIFTTSEAVAAEVADLSPLLAF<br>SILLNSVQPVLSGVAIGAGWQGYVAYVNLACYYLVGIPIGVILGYVVGLQVKGV<br>WIGMLFGIFVQTCVLTVMTLRTDWDQQVSTSLRNINRWVVPESRDANQISSEE
AT1G33110	MAGGGGELTAALLKKTAENGGEEKDELGLKQKVWIESKKLWIVAAPAIFTRFS<br>TFGVSIISQSFIGHLGPIELAAYSITFTVLLRFSNGILLGMASALETLCGQAYGAKQ<br>NHMLGIYLQRSWIVLTGCTICLTPVYIFSGPILLALGQEERIVRVARIIALWVIGIN<br>FSFVPSFTCQMFLQAQSKNKIIAYVAAVSLGVHVFLSWLLMVHFNFGITGAMTS<br>TLVAFWLPNIAQLLFVTCGGCKDTWRGFSMMAFKDLWPVFKLSMSSGGMLCL<br>ELWYNSILVLLTGNLKNAEVALDALAICLNINGLEMMIALGFLAAASVRVSNE<br>LGSGNPKGAKFATLTAVFTSLSLGIVLFFVFLFLRGRVSYIFTTSEAVAAEVADLS<br>PLLAFSILMNSVQPVLSGVAVGAGWQGYVTYVNLACYYLVGIPIGIILGYVVGL<br>QVKGVWIGMLFGIFVQTCVLTVMTLRTDWDQQVSTSLRRLNRWVVPESRDVN<br>QVSSEE
AT1G47530	MGKDKTLPLLDPREPPELTGTKSASKVWAKEFGEESKRLWELAGPAIFTAISQYS<br>LGALTQTFSGRLGELELAAVSVENSVISGLAFGVMLGMGSALETLCGQAYGAG<br>QIRMMGIYMQRSWVILFTTALFLLPVYIWAPPILSFFGEAPHISKAAGKFALWMI<br>PQLFAYAANFPIQKFLQSQRKVLVMAWISGVVLVIHAVFSWLFILYFKWGLVG<br>AAITLNTSWWLIVIGQLLYILITKSDGAWTGFSMLAFRDLYGFVKLSLASALMLC<br>LEFWYLMVLVVVTGLLPNPLIPVDAISICMNIEGWTAMISIGFNAAISVRVSNEL<br>GAGNAALAKFSVIVVSITSTLIGIVCMIVVLATKDSFPYLFTSSEAVAAETTRIAVL<br>LGFTVLLNSLQPVLSGVAVGAGWQALVAYVNIACYYIIGLPAGLVLGFTLDLGV<br>QGIWGGMVAGICLQTLILIGIIYFTNWNKEAEQAESRVQRWGGTAQE
AT1G51340	MMSEDGYNTDFPRNPLYIFFSDFRSVLKFDELGLEIARIALPAALALTADPIASLV<br>DTAFIGQIGPVELAAVGVSIALFNQVSRIAIFPLVSITTSFVAEEDACSSQQDTVR<br>DHKECIEIGINNPTEETIELIPEKHKDSLSDEFKTSSSIFSISKPPAKKRNIPSASSALI<br>IGGVLGLFQAVFLISAAKPLLSFMGVKHDSPMMRPSQRYLSLRSLGAPAVLLSL<br>AAQGVFRGFKDTTTPLFATVIGDVTNIILDPIFIFVFRLGVTGAATAHVISQYLMC<br>GILLWKLMGQVDIFNMSTKHLQFCRFMKNGFLLLMRVIAVTFCVTLSASLAAR

	EGSTSMAAFQVCLQVWLATSLLADGYAVAGQAILASAFAKKDYKRAAATASR<br>VLQLGLVLGFVLAVILGAGLHFGARVFTKDDKVLHLISIGLPFVAGTQPINALAF<br>VFDGVNFGASDFGYAAASLVMVAIVSILCLLFLSSTHGFIGLWFGLTIYMSLRAA<br>VGFWRIGTGTGPWSFLRS
AT1G58340	MCNSKPSSASSSLLSCKDKTHISKLETCDTDNPHYSEFRDTDSLDLKRWPSFLEG<br>LEEVKAIGKISGPTAMTGLLMYSRAMISMLFLGYLGELELAGGSLSIGFANITGYS<br>VISGLSMGMEPICGQAYGAKQMKLLGLTLQRTVLLLLSCSVPISFSWLNMRRILL<br>WCGQDEEISSVAQQFLLFAIPDLFLLSLLHPLRIYLRTQNITLPVTYSTAVSVLLH<br>VPLNYLLVVKLEMGVAGVAIAMVLTNLNLVVLLSSFVYFTSVHSDTWVPITIDS<br>LKGWSALLSLAIPTCVSVCLEWWWYEFMIILCGLLANPRATVASMGILIQTTAL<br>VYVFPSSLSLGVSTRISNELGAKRPAKARVSMIISLFCAIALGLMAMVFAVLVRH<br>HWGRLFTTDAEILQLTSIALPIVGLCELGNCPQTTGCGVLRGCARPTLGANINL<br>GSFYFVGMPVAILFGFVFKQGFPGLWFGLLAAQATCASLMLCALLRTDWKVQ<br>AERAEELTSQTPGKSPPLLPIASSKSRSTSGTEDMMRTMLV
AT1G61890	MNSESLENLHRPLIESSKSFVDYRLETVLTDRELPYFRRIYLAMMIEMKFLFHLA<br>APAIFVYVINNGMSILTRIFAGHVGSFELAAASLGNSGFNMFTYGLLLGMGSAV<br>ETLCGQAHGAHRYEMLGVYLQRSTVVLILTCLPMSFLFLFSNPILTALGEPEQVA<br>TLASVFVYGMIPVIFAYAVNFPIQKFLQSQSIVTPSAYISAATLVIHLILSWIAVYR<br>LGYGLLALSLIHSFSWWIIVVAQIVYIKMSPRCRRTWEGFSWKAFEGLWDFFRLS<br>AASAVMLCLESWYSQILVLLAGLLKNPELALDSLAICMSISAISFMVSVGFNAA<br>ASVRVSNELGAGNPRAAAFSTVVTTGVSFLLSVFEAIVVLSWRHVISYAFTDSPA<br>VAEAVADLSPFLAITIVLNGIQPVLSGVAVGCGWQAFVAYVNIGCYYVVGIPVG<br>FVLGFTYDMGAKGIWTGMIGGTLMQTIILVIVTLRTDWDKEVEKASSRLDQWE<br>ESREPLLKQ
AT1G64820	METDFSLVRKEEEEEEDNRNGMSYLSMEMMKKVSSMAAPMVAVSVSQFLLQV<br>ISMVMAGHLDELSLSAVAIATSLTNVTGFSLIVGFAGALDTLCGQAFGAEQFGK<br>IGAYTYSSMLCLLVFCFSISIVWFFMDKLLEIFHQDPLISQLACRYSIWLIPALFGFT<br>LLQPMTRYFQSQGITLPLFVSSLGALCFHIPFCWLLVYKLKFGIVGAALSIGFSYW<br>LNVFLLWIFMRYSALHREMKNLGLQELISSMKQFIALAIPSAMMICLEWWSFEIL<br>LLMSGLLPNSKLETSVISICLTTSAVHFVLVNAIGASASTHVSNELGAGNHRAAR<br>AAVNSAIFLGGVGALITTITLYSYRKSWGYVFSNEREVVRYATQITPILCLSIFVNS<br>FLAVLSGVARGSGWQRIGGYASLGSYYLVGIPLGWFLCFVMKLRGKGLWIGILI<br>ASTIQLIVFALVTFFTNWEQEATKARDRVFEMTPQVKGNQKTQIIVEEDTQVLL<br>NHIAETV
AT1G66760	MKKSIETPLLLNTKQSQDEDKEKIRWEKMKKVASMAAPMVAVNMSQYLLQAT<br>STMIVGHRSELALAGIALGSSFANVTGFGVLFGLSGSLETLCGQAYGAKQYHKL<br>GSYTFTSIVFLLIISVPISILWMFMNQILLLLHQDPQIAELAGVYCLWLVPALFGYS<br>VLESLVRYFQSQSLIYPMVLSSLAALSFHVPLCWLMVHKFDFGAKGAAASIGISY<br>WLNAVFLWVYMKRSSRCVETRIYMSKDVFVHTNIFFQFAIPSAMMCCLEWLAF<br>EVITLLSGLLPNSKLETSVISICLTTSSLHYNLVNGIGDAASTNVANELGAGNPRG<br>ARDSAAAAIIIAAVESVIVSSSLFLSRSVWPYAYSNVEEVISYVTDITPILCISILMDS<br>FLTVLSGIVRGTGWQKIGAYVNITSYYVIGIPVGLLLCFHLHFNGKGLWAGLVT<br>GSTLQTLILFLVIGFTNWSKEAIKARERIGDEKVWRHDSLLN
AT1G6	MENGFSLVPKEEEEEEDYSNEKSEDQTSYYLSTEMMKKVSFMAAPMVAVAASQ

6780	YLLQVISIVMAGHLDELSLSAVAIATSLTNVTGFSLIFGLAGALETLCGQAFGAG<br>QFRNISAYTYGSMLCLLLVCFPISLLWVFMDKLLELFHQDPLISQLACRYSIWLIP<br>ALFGYSVLQSMTRFFQSQGLVLPLFLSSLGALFFHVPFSWLLVYKLRFGIVGAAL<br>SIGFSYWLNVGLLWAFMRDSALYRKNWNLRAQEIFLSMKQFITLAIPTAMMTC<br>LEWWSFELLILMSGLLPNSKLETSVLSICLTMSSLHYVIVNAIGAAASTHVSNKL<br>GAGNPKAARSAANSAIFLGMIDAAIVSISLYSYRRNWAYIFSNESEVADYVTQIT<br>PFLCLSIGVDSFLAVLSGVARGTGWQHIGAYANIGSYYLVGIPVGSILCFVVKLR<br>GKGLWIGILVGSTLQTIVLALVTFFTNWEQEVAKARDRVIEMIPQEII
AT1G71140	MDSAEKGLLVVSDREEVNKKDGFLRETKKLSYIAGPMIAVNSSMYVLQVISIMM<br>VGHLGELFLSSTAIAVSFCSVTGFSVVFGLASALETLCGQANGAKQYEKLGVHT<br>YTGIVSLFLVCIPLSLLWTYIGDILSLIGQDAMVAQEAGKFATWLIPALFGYATLQ<br>PLVRFFQAQSLILPLVMSSVSSLCIHIVLCWSLVFKFGLGSLGAAIAIGVSYWLNV<br>TVLGLYMTFSSSCSKSRATISMSLFEGMGEFFRFGIPSASMICLEWWSFEFLVLLSG<br>ILPNPKLEASVLSVCLSTQSSLYQIPESLGAAASTRVANELGAGNPKQARMAVY<br>TAMVITGVESIMVGAIVFGARNVFGYLFSSETEVVDYVKSMAPLLSLSVIFDALH<br>AALSGVARGSGRQDIGAYVNLAAYYLFGIPTAILLAFGFKMRGRGLWIGITVGS<br>CVQAVLLGLIVILTNWKKQARKARERVMGDEYEEKESEEEHEYIS
AT1G71870	MEDKIQSDDFTSHKNPTLPQVIEELKELWAMVLPITAMNCLVYVRAVVSVLFL<br>GRLGSLELAGGALSIGFTNITGYSVMVGLASGLEPVCSQAYGSKNWDLLTLSLH<br>RMVVILLMASLPISLLWINLGPIMLFMGQNPEITATAAEYCLYALPDLLTNTLLQ<br>PLRVYLRSQRVTKPMMWCTLAAVAFHVPLNYWLVMVKHWGVPGVAIASVVT<br>NLIMVVLLVGYVWVSGMLQKRVSGDGDGGSTTMVAVVAQSSSVMELVGGLG<br>PLMRVAVPSCLGICLEWWWYEIVIVMGGYLENPKLAVAATGILIQTTSLMYTVP<br>MALAGCVSARVGNELGAGRPYKARLAANVALACAFVVGALNVAWTVILKER<br>WAGLFTGYEPLKVLVASVMPIVGLCELGNCPQTTGCGILRGTGRPAVGAHVNL<br>GSFYFVGTPVAVGLAFWLKIGFSGLWFGLLSAQAACVVSILYAVLARTDWEGE<br>AVKAMRLTSLEMRKVGQDEESSLLLLDDEKLGDVL
AT1G73700	MEDGVTPPLLITEKDTTMIRVKEEVKKQLWLSAPLIGVSLLQYSLQVISVMFVGH<br>LGSLPLSAASIATSFASVTGFTFLLGTASALETLCGQAYGAKLYGKLGIQMQRA<br>MFVLLILSVPLSIIWANTEQILVLVHQDKSIASVAGSYAKYMIPSLFAYGLLQCIN<br>RFLQAQNNVFPVFVCSGITTCLHLLLCWLFVLKTGLGYRGAALAISVSYWFNVI<br>LLSCYVKFSPSCSHSWTGFSKEAFQELYDFSKIAFPSAVMVCLELWSFELLVLASG<br>LLPNPVLETSVLSICLNTSLTIWQISVGLGGAASIRVSNELGAGNPQVAKLAVYV<br>IVGIAVAEGIVVVTVLLSIRKILGHAFSSDPKIIAYAASMIPIVACGNFLDGLQCVL<br>SGVARGCGWQKIGACVNLGSYYLVGVPLGLLLGFHFHIGGRGLWLGIVTALSV<br>QVLCLSLVTIFTNWDKEAKKATNRVGSSDDKDGDVQ
AT2G04040	MEEPFLLRDELLVPSQVTWHTNPLTVELKRVSRLAAPMATVTIAQYLLPVISVM<br>VAGHNGELQLSGVALANSFTNVTGFSIMCGLVGALETLCGQAYGAKQYEKIGT<br>YAYSAIASNIPICFLISILWLYIEKILISLGQDPEISRIAGSYAFWLIPALFGQAIVIPL<br>SRFLLTQGLVIPLLFTAVTTLLFHVLVCWTLVFLFGLGCNGPAMATSVSFWFYA<br>VILSCYVRFSSSCEKTRGFVSRDFVSSIKQFFQYGIPSAAMICLEWWLFEILILCSGL<br>LPNPKLETSVLSICLTIETLHYVISAGVAAAVSTRVSNNLGAGNPQVARVSVLAG<br>LCLWIVESAFFSILLFTCRNIIGYAFSNSKEVLDYVADLTPLLCLSFILDGFTAVLN<br>GVARGSGWQHIGAWNNTVSYYLVGAPVGIYLAFSRELNGKGLWCGVVVGSTV

	QATILAIVTASINWKEQAEKARKRIVSTENRLA
AT2G04050	MEEPFLLQDEHLVPCKDTWKSGQVTVELKKVSSLAAPMAAVTIAQYLLPVISVMVAGHNGELQLSGVALATSFTNVSGFSILFGLAGALETLCGQAYGAKQYEKIGTYTYSATASNIPICVLISVLWIYIEKLLISLGQDPDISRVAGSYALWLIPALFAHAFFIPLTRFLLAQGLVLPLLYCTLTTLLFHIPVCWAFVYAFGLGSNGAAMAISVSFWFYVVILSCYVRYSSSCDKTRVFVSSDFVSCIKQFFHFGVPSAAMVCLEWWLFELLILCSGLLPNPKLETSVLSICLTTASLHYVIPGGVAAAVSTRVSNKLGAGIPQVARVSVLAGLCLWLVESAFFSTLLFTCRNIIGYAFSNSKEVVDYVANLTPLLCLSFILDGFTAVLNGVARGSGWQHIGALNNVVAYYLVGAPVGVYLAFNRELNGKGLWCGVVVGSAVQAIILAFVTASINWKEQAEKARKRMVSSENRLA
AT2G04070	MEEPFLPQDEQIVPCKATWKSGQLNVELKKVSRLAVPMATVTIAQYLLPVISVMVAGHNGELQLSGVALATSFTNVSGFSIMFGLVGSLETLSGQAYGAKQYEKMGTYTYSAISSNIPICVLISILWIYMEKLLISLGQDPDISRVAGSYALRLIPTLFAHAIVLPLTRFLLAQGLVLPLLYFALTTLLFHIAVCWTLVSALGLGSNGAALAISVSFWFFAMTLSCYVRFSSSCEKTRRFVSQDFLSSVKQFFRYGVPSAAMLCLEWWLFELLILCSGLLQNPKLETSVLSICLTTATLHYVIPVGVAAAVSTRVSNKLGAGIPQVARVSVLAGLCLWLVESSFFSILLFAFRNIIGYAFSNSKEVVDYVADLSPLLCLSFVLDGFTAVLNGVARGCGWQHIGALNNVVAYYLVGAPVGIYLAFSCELNGKGLWCGVVVGSAVQAIILAIVTASMNWKEQAKKARKRLISSENGLA
AT2G04080	MEEPFLPRDEQLVSCKSTWQSGQVTVELKKVSRLAAPMATVTIAQYLLPVISVMVAGHIGELELAGVALATSFTNVSGFSIMFGLVGALETLCGQAYGAEQYEKIGTYTYSAMASNIPICFIISILWIYIEKLLITLGQEPDISRVAGSYSLWLVPALFAHAIFLPLTRFLLAQGLVISLLYSAMTTLLFHIAVCWTLVFALGLGSNGAAIAISLSFWFYAVILSCHVRFFSSCEKTRGFVSNDFMSSIKQYFQYGVPSAGLICLEWWLFELLILCSGLLPNPKLETSVLSICLTIGTLHYVIPSGVAAAVSTRVSNKLGAGNPQVARVSVLAGLCLWLVESAFFSTLLFTCRNIIGYTFSNSKEVVDYVADISPLLCLSFILDGLTAVLNGVARGCGWQHIGALINVVAYYLVGAPVGVYLAFSREWNGKGLWCGVMVGSAVQATLLAIVTASMNWKEQAEKARKRIISTENGLV
AT2G04090	MEDPLLLGDDQLITRNLKSTPTWWMNFTAELKNVSSMAAPMATVTVSQYLLPVISVMVAGHCGELQLSGVTLATAFANVSGFGIMYGLVGALETLCGQAYGAKQYTKIGTYTFSAIVSNVPIVVLISILWFYMDKLFVSLGQDPDISKVAGSYAVCLIPALLAQAVQQPLTRFLQTQGLVLPLLYCAITTLLFHIPVCLILVYAFGLGSNGAALAIGLSYWFNVLILALYVRFSSACEKTRGFVSDDFVLSVKQFFQYGIPSAAMTTIEWSLFELLILSSGLLPNPKLETSVLSICLTTSSLHCVIPMGIGAAGSTRISNELGAGNPEVARLAVFAGIFLWFLEATICSTLLFTCKNIFGYAFSNSKEVVDYVTELSSLLCLSFMVDGFSSVLDGVARGSGWQNIGAWANVVAYYLLGAPVGFFLGFWGHMNGKGLWIGVIVGSTAQGIILAIVTACLSWEEQAAKARERIVGRTLE
AT2G04100	MEDPLLLGDNQIITGSLKPTPTWRMNFTAELKNLSRMALPMATVTVAQYLLPVISVMVAGHRSELQLSGVALATSFTNVSGFSVMFGLAGALETLCGQAYGAKQYAKIGTYTFSAIVSNVPIVVLISILWFYMDKLFVSLGQDPDISKVAGSYAVCLIPALLAQAVQQPLTRFLQTQGLVLPLLYCAITTLLFHIPVCLILVYAFGLGSNGAALAIGLSYWFNVLILALYVRFSSSCEKTRGFVSDDFVLSVKQFFQYGIPSAAMTTIEWSLFEFLILSSGLLPNPKLETSVLSICLTTSSLHYVIPMGIGAAGSIRVSNELGAGNPEVARLAVFAGIFLWFLEATICSTLLFICRDIFGYAFSNSKEVVDYVTELSPLLCISFLVDGF

	SAVLGGVARGSGWQHIGAWANVVAYYLLGAPVGLFLGFWCHMNGKGLWIG<br>VVVGSTAQGIILAIVTACMSWNEQAAKARQRIVVRTSSFGNGLA
AT2G21340	MQIQCKTLTFTVSSIPCNPKLPFPSSLTLRSWNPSFPSFRSSAVSGPKSSLKLNRFL<br>RNCASTNQELVVDGETGNGSISELQGDAANGSISPVEVEAEVEEVKVDDLATQ<br>SIWGQMKEIVMFTGPAAGLWLCGPLMSLIDTAVIGQGSSLELAALGPATVICDY<br>LCYTFMFLSVATSNLVATSLARQDKDEVQHQISILLFIGLACGVTMMVLTRLFGS<br>WALTAFTGVKNADIVPAANKYVQIRGLAWPAVLIGWVAQSASLGMKDSWGP<br>LKALAVASAINGVGDVVLCTFLGYGIAGAAWATMVSQVVAAYMMMDALNK<br>KGYSAFSFCVPSPSELLTIFGLAAPVFITMMSKVLFYTLLVYFATSMGTNIIAAHQ<br>VMLQIYTMSTVWGEPLSQTAQSFMPELLFGINRNLPKARVLLKSLVIIGATLGIV<br>VGTIGTAVPWLFPGIFTRDKVVTSEMHKVIIPYFLALSITPSTHSLEGTLLAGRDL<br>RYISLSMTGCLAVAGLLLMLLSNGGFGLRGCWYALVGFQWARFSLSLFRLLSRD<br>GVLYSEDTSRYAEKVKAA
AT2G34360	MREEREDMLSWPLIGEKEKRSRFVKEEVEKQLLLSGPLIAVSLLQFCLQIISVMFV<br>GHLGSLPLSAASIATSFASVTGFTFLMGTASAMDTVCGQSYGAKMYGMLGIQM<br>QRAMLVLTLLSVPLSIVWANTEHFLVFFGQDKSIAHLSGSYARFMIPSIFAYGLL<br>QCLNRFLQAQNNVIPVVICSGVTTSLHVIICWVLVLKSGLGFRGAAVANAISYW<br>LNVILLSCYVKFSPSCSLTWTGFSKEARRDIIPFMKLVIPSAFMVCSLEMWSFELL<br>VLSSGLLPNPVLETSCPRTVWMIPFGLSGAASTRVSNELGSGNPKGAKLAVRVV<br>LSFSIVESILVGTVLILIRKIWGFAYSSDPEVVSHVASMLPILALGHSLDSFQTVLSG<br>VARGCGWQKIGAFVNLGSYYLVGVPFGLLLGFHFHVGGRGLWLGIICALIVQG<br>VCLSLITFFTNWDEEVKKATSRAKSSSEVKEFAVDNGSILV
AT2G38330	MAAVATSFCFSPHRSPSRFGNPNSSIRRTIVCKSSPRDESPAVSTSSQRPEKQQNP<br>LTSQNKPDHDHKPDPGIGKIGMEIMSIALPAALALAADPITSLVDTAFVGHIGS<br>AELAAVGVSVSVFNLVSKLFNVPLLNVTTSFVAEEQAIAAKDDNDSIETSKKVL<br>PSVSTSLVLAAGVGIAEAIALSLGSDFLMDVMAIPFDSPMRIPAEQFLRLRAYGA<br>PPIVVALAAQGAFRGFKDTTTPLYAVVAGNVLNAVLDPILIFVLGFGISGAAAA<br>TVISEYLIAFILLWKLNENVVLLSPQIKVGRANQYLKSGGLLIGRTVALLVPFTLA<br>TSLAAQNGPTQMAGHQIVLEIWLAVSLLTDALAIAAQSLLATTYSQGEYKQAR<br>EVLFGVLQVGLATGTGLAAVLFITFEPFSSLFTTDSEVLKIALSGTLFVAGSQPVN<br>ALAFVLDGLYYGVSDFGFAAYSMVIVGFISSLFMLVAAPTFGLAGIWTGLFLFM<br>ALRLVAGAWRLGTRTGPWKMLWSAPEKPE
AT2G38510	MQVGEEMASLTKIACPIVMTSLLIFSRSIISMWFLSHLGKVELAGGALAMGFGNI<br>TGVSVLKGLSVGMDPICGQAFGAKRWTVLSHTFQKMFCLLIVVSVPIAVTWLNI<br>EPIFLRLGQDPDITKVAKTYMLFFVPELLAQAMLHPLRTFLRTQGLTSPLTISAIV<br>SILLHPLFNYVFVVRMRLGVKGVAIAMAFNTMNIDVGLLVYTCFSDSLIKPWEG<br>LALRSLFRGWWPLLSLAAPSAISVCLEYWWYEIMLFLCGLLGNPKASVAAMGIL<br>IQTTGILYVVPFAISSAIATRVGHALGGGQPTRAQCTTVIGLILAVAYGLAAAVF<br>VTALRSVWGKMFTDEPEILGLISAALPILGLCEIGNSPQTAACGVLTGTARPKDG<br>ARVNLCAFYIVGLPVAVTTTFGFKVGFRGLWFGLLSAQMTCLVMMLYTLIRTD<br>WSHQVKRAEELTSAAADKSHSEDETVHAEVQDDDDVSSNDLEIGLLQNTN
AT3G03620	MSTQEEMEERLLREGSDAEGQSNNRESIYLRTKVWSEVNKMWRIALPSSLFRMT<br>SFGSIIVAQAFIGHSSELGLAAYALLQSTFIRFLYGLMGGMSSATETLCGQAYGA<br>EQYHTMGIYLQRSWIVDMAVTTLFLPFIVLAGPILRLLGQNVEITKTVDEIYPWM

	IPYVYSLIFTMTIQMYLQAQMRNAIVGVLSTLSLALDLVVTWWCVSVMGMGIGGALLGLNVGSWAMVLAEFVYIFGGWCPFTWTGFSIAAFVDLIPMLKLSISSGFMICLEYWYMSILVLMAGYTKDAKIAISAFSICQYIYTWELNICLGFLGAACVRVANELGKGDAHAVRFSIKVILTISTLMGVIFSALCLAFCGRISYLFSNSDEVSDAVNDLSVILAVSILLNSIQPILSGVAVGAGMQSIVAVVNLASYYAIGIPLGLILTYVFHLGVKGLWSGMLAGIAIQTIILCYIIYKTDWELEVKRTCERMKVWSLKPSNEESNPIIREESRSK
AT3G08040	MTETGDDLATVKKPIPFLVIFKDLRHVFSRDTTGREILGIAFPAALALAADPIASLIDTAFVGRLGAVQLAAVGVSIAIFNQASRITIFPLVSLTTSFVAEEDTMEKMKEEANKANLVHAETILVQDSLEKGISSPTSNDTNQPQQPPAPDTKSNSGNKSNKKEKRTIRTASTAMILGLILGLVQAIFLIFSSKLLLGVMGVKPNSPMLSPAHKYLSIRALGAPALLLSLAMQGIFRGFKDTKTPLFATVVADVINIVLDPIFIFVLRLGIIGAAIAHVISQYFMTLILFVFLAKKVNLIPPNFGDLQFGRFLKNGLLLLARTIAVTFCQTLAAAMAARLGTTPMAAFQICLQVWLTSSLLNDGLAVAGQAILACSFAEKDYNKVTAVASRVLQMGFVLGLGLSVFVGLGLYFGAGVFSKDPAVIHLMAIGIPFIAATQPINSLAFVLDGVNFGASDFAYTAYSMVGVAAISIAAVIYMAKTNGFIGIWIALTIYMALRAITGIARMATGTGPWRFLRGRSSSSSS
AT3G21690	MDSSPNDGVHQPLLHPQPSPSPPESTNGELETVLSDVETPLFLRLRKATIIESKLLFNLAAPAVIVYMINYLMSMSTQIFSGHLGNLELAAASLGNTGIQVFAYGLMLGMGSAVETLCGQAYGGRKYEMLGVYLQRSTVLLTLTGLLLTLIYVFSEPILLFLGESPAIASAASLFVYGLIPQIFAYAANFPIQKFLQSQSIVAPSAYISTATLFVHLLLSWLAVYKLGMGLLGASLVLSLSWWIIVVAQFVYIVTSERCRETWRGFSVQAFSGLWSFFKLSAASAVMLCLETWYFQILVLLAGLLENPELALDSLSICMTISGWVFMISVGFNAAISVRVSNELGAGNPKSAAFSVIIVNIYSLITCVILAIVILACRDVLSYAFTEGKEVSDAVSDLCPLLAVTLVLNGIQPVLSGVAVGCGWQTFVAKVNVGCYYIIGIPLGALFGFYFNFGAKGIWTGMIGGTVIQTFILAWVTFRTDWTKEVEEASKRLDKWSNKKQEVVPE
AT3G23550	MADPTSKDDHDGEGGRDKSSTFVQKLIDVEEAKTQIIYSLPMIFTNLFYYCIPLTSVMFASQLGQLELAGATLANSWATVTGFAFMTGLSGALETLCGQGFGAKSYRMLGIHLQSSCIVSLVFTILITILWFFTESVFLLLRQDPSISKQAALYMKYLAPGLLAYGFLQNILRFCQTQCIVTPLVLFSFLPLVINIGTTYALVHLAGLGFIGAPIATSISLWIAFVSLGFYVICSDKFKETWTGFSMESFHHVVLNLTLSIPSAAMVCLEYWAFEILVFLAGLMRNPEITTSLVAICVNTESISYMLTCGLSAATSTRVSNELGAGNVKGAKKATSVSVKLSLVLALGVVIAILVGHDAWVGLFSNSHVIKEGFASLRFFLAASITLDSIQGVLSGVARGCGWQRLATVINLGTFYLIGMPISVLCGFKLKLHAKGLWIGLICGMFCQSASLLLMTIFRKWTKLTAATV
AT3G23560	MADPATSSPLLDDHVGGEDERGRRSRSSTLVQKVIDVEEAKAQMIYSLPMILTNVFYYCIPITSVMFASHLGQLELAGATLANSWATVSGFAFMVGLSGSLETLCGQGFGAKRYRMLGVHLQSSCIVSLVFSILITIFWFFTESIFGLLRQDPSISKQAALYMKYQAPGLLAYGFLQNILRFCQTQSIIAPLVIFSFVPLVINIATAYVLVYVAGLGFIGAPIATSISLWIAFLSLGTYVMCSEKFKETWTGFSLESFRYIVINLTLSLPSAAMVCLEYWAFEILVFLAGVMPNPEINTSLVAICVNTEAISYMLTYGLSAAASTRVSNELGAGNVKGAKKATSVSVKLSLVLALGVVIVLLVGHDGWVGLFSDSYVIKEEFASLRFFLAASITLDSIQGVLSGVARGCGWQRLVTVINLATFYLIGMPIAAFCGFKLKFYA

	KGLWIGLICGIFCQSSSLLLMTIFRKWTKLNVATV
AT3G26590	MAKDKDITETLLTAAEERSDLPFLSVDDIPPITTVGGFVREFNVETKKLWYLAGP<br>AIFTSVNQYSLGAITQVFAGHISTIALAAVSVENSVVAGFSFGIMLGMGSALETL<br>CGQAFGAGKLSMLGVYLQRSWVILNVTALILSLLYIFAAPILASIGQTAAISSAA<br>GIFSIYMIPQIFAYAINFPTAKFLQSQSKIMVMAVISAVALVIHVPLTWFVIVKLQ<br>WGMPGLAVVLNASWCFIDMAQLVYIFSGTCGEAWSGFSWEAFHNLWSFVRLS<br>LASAVMLCLEVWYFMAIILFAGYLKNAEISVAALSICMNILGWTAMIAIGMNT<br>AVSVRVSNELGANHPRTAKFSLLVAVITSTLIGFIVSMILLIFRDQYPSLFVKDEK<br>VIILVKELTPILALSIVINNVQPVLSGVAVGAGWQAVVAYVNIACYYVFGIPFGL<br>LLGYKLNYGVMGIWCGMLTGTVVQTIVLTWMICKTNWDTEASMAEDRIREW<br>GGEVSEIKQLIN
AT3G59030	MSSTETYEPLLTRLHSDSQITERSSPEIEEFLRRRGSTVTPRWWLKLAVWESKLLW<br>TLSGASIVVSVLNYMLSFVTVMFTGHLGSLQLAGASIATVGIQGLAYGIMLGMA<br>SAVQTVCGQAYGARQYSSMGIICQRAMVLHLAAAVFLTFLYWYSGPILKTMGQ<br>SVAIAHEGQIFARGMIPQIYAFALACPMQRFLQAQNIVNPLAYMSLGVFLLHTL<br>LTWLVTNVLDFGLLGAALILSFSWWLLVAVNGMYILMSPNCKETWTGFSTRAF<br>RGIWPYFKLTVASAVMLCLEIWYNQGLVIISGLLSNPTISLDAISICMYYLNWDM<br>QFMLGLSAAISVRVSNELGAGNPRVAMLSVVVVNITTVLISSVLCVIVLVFRVGL<br>SKAFTSDAEVIAAVSDLFPLLAVSIFLNGIQPILSGVAIGSGWQAVVAYVNLVTY<br>YVIGLPIGCVLGFKTSLGVAGIWWGMIAGVILQTLTLIVLTLKTNWTSEVENAA<br>QRVKTSATENQEMANAGV
AT4G00350	MEIPVREERRSSSSSAGPLQQTISLAADDAIDSGPSSPLVVKVSVFETEHETTKLIH<br>APSTLLGETTGDADFPPIQSFRDAKLVCVVETSKLWEIAAPIAFNILCNYGVNSF<br>TSIFVGHIGDLELSAVAIALSVVSNFSFGFLLGMASALETLCGQAFGAGQMDML<br>GVYMQRSWLILLGTSVCLLPLYIYATPLLILLGQEPEIAEISGKFTTQIIPQMFALAI<br>NFPTQKFLQSQSKVGIMAWIGFFALTLHIFILYLFINVFKWGLNGAAAAFDVSA<br>WGIAIAQVVYVVGWCKDGWKGLSWLAFQDVWPFLKLSFASAVMLCLEIWYF<br>MTIIVLTGHLEDPVIAVGSLSICMNINGWEGMLFIGINAAISVRVSNELGSGHPR<br>AAKYSVIVTVIESLVIGVVCAIVILITRDDFAVIFTESEEMRKAVADLAYLLGITMI<br>LNSLQPVISGVAVGGGWQAPVAYINLFCYYAFGLPLGFLLGYKTSLGVQGIWIG<br>MICGTSLQTLILLYMIYITNWNKEVEQASERMKQWGAGYEKLEKIAT
AT4G21903	MNGSNETVERRIELRRPLVDTEKKLPLEVGLESVLTESSLPYRRRVYLGMCIELKL<br>LLRLALPAILVYLINGGMGISARIFAGHLGSTQLAAASIGNSSFSLVYALMLGMG<br>SAVETLCGQAYGAHRYEMLGIYLQRATIVLALVGFPMTILYTFSYPILLLLLGEPKT<br>VSYMGSLYIAGLIPQIFAYAVYFTAQKFLQAQSVVAPSAYISAAALVLQISLTWIT<br>VYAMGQGLMGIAYVLTISWWFIVGAQTFYVITSVRFKDTWTGFSWKSLHGLWS<br>FFKLSAGSAVMICLELWYTQILVLLAGLLKDPALSLDSLSICMSISALSFMVSVGF<br>NAAVSVRTSNELGAGNPKSALFSTWTATFVSFVISVVEALVVIASRDNVSYIFTS<br>DADVAKAVSDLCPFLAVTIILNGIQPVLSGVAVGCGWQTYVAYVNIGCYYIVGI<br>PIGCILGFTFNFQAKGIWTGMIGGTLMQTLILLYVTYQADWDKEVMLHEIKLKK<br>RESDWICGTTKSLSRISNFVMK
AT4G21910	MEVPSETTNLADLRRPLVVPVVSERKPPADVGLGLESVLTERSLPYRRRVYLGA<br>CIEMKLLFRLALPAILVYLVNSGMGISARIFAGHLGKNELAAASIGNSCFSLVYG<br>LMLGMGSAVETLCGQAYGAHRYEMLGIYLQRATIVLALVGLPMTLLYTFSYPIL

	ILLGEPKTVSYMGSKYIAGLIPQIFAYAVNFTAQKFLQAQSVVAPSAFISAAALILQILLTWITVYVMDMGFMGIAYVLTISWWVIVGSQCFYIAVSPKFRHTWTGLSWRSLQGLWSFFKLSAGSAVMICLEMWYSQILVLLAGLLENPARSLDSLSICMSISALSFMVSVGFNAAVSVRTSNELGAGNPKSAWFSTWTATFVSFVISVTEALAVIWFRDYVSYIFTEDADVAKAVSDLCPFLAITIILNGIQPVLSGVAVGCGWQTYVAYVNVGCYYVVGIPVGCILGFTFDFQAKGIWTGMIGGTLMQTLILLYVTYRTDWDKEVMLHEIKWKKRGNVWICGTTRRSLSKTSYNKFGGVIDNKEKEISVVCAGDFNVRSFSWLLCLFYGNRTIKIYFCLMDYSLFTKSCS
AT4G22790	MSETSKSESLDPEVSEGLCSKTLMQSIVHELKLQMRIGLPLVVMNLLWFGKMTTTSVFLGRQGELNLAGGSLGFSFANVTGFSVLYGISAAMEPICGQAFGAKNFKLLHKTLFMAVLLLLLISVPISFLWLNVHKILTGFGQREDISFIAKKYLLYLLPELPILSFLCPLKAYLSSQGVTLPIMFTTAAATSLHIPINIVLSKARGIEGVAMAVWITDFIVVILLTGYVIVVERMKENKWKQGGWLNQSAQDWLTLIKLSGPCCLTVCLEWWCYEILVLLTGRLPNPVQAVSILIIVFNFDYLLYAVMLSLGTCVATRVSNELGANNPKGAYRAAYTTLIVGIISGCIGALVMIAFRGFWGSLYTHHDQLILNGVKKMMLIMAVIEVVNFPLMVCGEIVRGTAKPSLGMYANLSGFYLLALPLGATLAFKAKQGLQGFLIGLFVGISLCLSILLIFIARIDWEKEAGKAQILTCNTEDEQTSQGSGQDSHS
AT4G23030	MAAPLLMIIKNQTDHRQDPNPNPTHLSSSIQEAKSIAKISLPLILTGLLLYSRSMISMLFLGRLNDLSALSGGSLALGFANITGYSLLSGLSIGMEPICVQAFGAKRFKLLGLALQRTTLLLLLCSLPISILWLNIKKILLFFGQDEEISNQAEIFILFSLPDLILQSFLHPIRIYLRSQSITLPLTYSAFFAVLLHIPINYLLVSSLGLGLKGVALGAIWTNVNLLGFLIIYIVFSGVYQKTWGGFSMDCFKGWRSLMKLAIPSCVSVCLEWWWYEIMILLCGLLLNPQATVASMGILIQTTALIYIFPSSLSISVSTRVGNELGANQPDKARIAARTGLSLSLGLGLLAMFFALMVRNCWARLFTDEEEIVKLTSMVLPIIGLCELGNCPQTTLCGVLRGSARPKLGANINLCCFYFVGMPVAVWLSFFSGFDFKGLWLGLFAAQGSCLISMLVVLARTDWEVEVHRAKELMTRSCDGDEDDGNTPFLLDSLDIEENLVF
AT4G25640	MDPTAPLLTHGGEVEEDYAPARSWTDVKRVLSTESAKLWMIAAPVGFNIICQYGVSSVTNIFVGHIGEVELSAVSISLSVIGTFSFGFLLGMGSALETLCGQAYGAGQVNMLGVYMQRSWIILFVSCFFLLPIYIFATPVLRLLGQAEEIAVPAGQFTLLTIPQLFSLAFNFPTSKFLQAQSKVVAIAWIGFVALSLHVIMLWLFIIEFGWGTNGAALAFNITNWGTAIAQIVYVIGWCNEGWTGLSWLAFKEIWAFVRLSIASAVMLCLEIWYMMSIIVLTGRLDNAVIAVDSLSICMNINGLEAMLFIGINAAISVRVSNELGLGRPRAAKYSVYVTVFQSLLIGLVFMVAIIIARDHFAIIFTSSKVLQRAVSKLAYLLGITMVLNSVQPVVSGVAVGGGWQGLVAYINLGCYYIFGLPFGYLLGYIANFGVMGLWSGMIAGTALQTLLLLIVLYKTNWNKEVEETMERMKKWGGSETTSKDILASGWPSGLRRQTQVLVFVRGRGFKPHF
AT4G29140	MCNPSTTTTTTGSENQESRTGLFLDLFSINSFEPTKRNLRHCENRGSPLMAEAVTEAKSLFTLAFPIAVTALVLYLRSAVSMFFLGQLGDLELAAGSLAIAFANITGYSVLSGLALGMEPLCSQAFGAHRFKLLSLTLHRTVVFLLVCCVPISVLWFNVGKISVYLHQDPDIAKLAQTYLIFSLPDLLTNTLLHPIRIYLRAQGIIHPVTLASLSGAVFHLPANLFLVSYLRLGLTGVAVASSITNIFVVAFLVCYVWASGLHAPTWTDPTRDCFRGWAPLLRLAGPSCVSVCLEWWWYEIMIVLCGLLVNPRSTVAAMGVLIQTTSFLYVFPSSLSFAVSTRVGNELGANRPKTAKLTATVAIVFAAVTGIIAAAFAYSVRNA

	WGRIFTGDKEILQLTAAALPILGLCEIGNCPQTVGCGVVRGTARPSTAANVNLG<br>AFYLVGMPVAVGLGFWAGIGFNGLWVGLLAAQISCAGLMMYVVGTTDWESE<br>AKKAQTLTCAETVENDIIKAVVASTIDGECDEAEPLIRITVLY
AT4G3<br>8380	MESSRVVVGGGLPLANRRNSSFAKPKIQQGTFLPLSRINNVSAPQKCSLHTNPN<br>PMFPFVTRRKSQTNPDCGVVKLGEEDDSCSSLDKLPEVNGVHTGVARPVDIKR<br>ELVMLSLPAIAGQAIDPLTLLMETAYIGRLGSVELGSAGVSMAIFNTISKLFNIPL<br>LSVATSFVAEDIAKIAAQDLASEDSQSDIPSQGLPERKQLSSVSTALVLAIGIGIFE<br>ALALSLASGPFLRLMGIQSMSEMFIPARQFLVLRALGAPAYVVSLALQGIFRGFK<br>DTKTPVYCLGIGNFLAVFLFPLFIYKFRMGVAGAAISSVISQYTVAILMLILLNKR<br>VILLPPKIGSLKFGDYLKSGGFVLGRTLSVLVTMTVATSMAARQGVFAMAAHQI<br>CMQVWLAVSLLTDALASSGQALIASSASKRDFEGVKEVTTFVLKIGVVTGIALAI<br>VLGMSFSSIAGLFSKDPEVLRIVRKGVLFVAATQPITALAFIFDGLHYGMSDFPY<br>AACSMMVVGGISSAFMLYAPAGLGLSGVWVGLSMFMGLRMVAGFSRLMWRK<br>GPWWFMHTSDKRLA
AT4G3<br>9030	MLIKSQRLTLFSPLLSKTRRIPVNSHQTLVAESVITRRTLGAITATPSFHKNPVVIR<br>RRIKLERVTRNCVRIDREIDEEEEEEEKERGDLVKQSIWEQMKEIVKFTGPAMG<br>MWICGPLMSLIDTVVIGQGSSIELAALGPGTVLCDHMSYVFMFLSVATSNMVAT<br>SLAKQDKKEAQHQISVLLFIGLVCGLMMLLLTRLFGPWAVTAFTRGKNIEIVPA<br>ANKYIQIRGLAWPFILVGLVAQSASLGMKNSWGPLKALAAATIINGLGDTILCL<br>FLGQGIAGAAWATTASQIVSAYMMMDSLNKEGYNAYSFAIPSPQELWKISALA<br>APVFISIFSKIAFYSFIIYCATSMGTHVLAAHQVMAQTYRMCNVWGEPLSQTAQ<br>SFMPEMLYGANRNLPKARTLLKSLMIIGATLGLVLGVIGTAVPGLFPGVYTHDK<br>VIISEMHRLLIPFFMALSALPMTVSLEGTLLAGRDLKFVSSVMSSSFIIGCLTLMFV<br>TRSGYGLLGCWFVLVGFQWGRFGLYLRRLLSPGGILNSDGPSPYTVEKIKSI
AT5G1<br>0420	MDKKSGGTKAIEEATVPLLECHNAAEEGGGMKREIWIETKKIWYIVGPSIFTGL<br>ATYSILIITQAFAGHLGDLELAAISIINNFTLGFNYGLLLGMASALETLCGQAFGA<br>REYYMLGVYMQRYWIILFLCCILLLPMYLFATPILKFIGQSDDIAELTGTIALWVI<br>PVHFAFAFFFPLNRFLQCQLKNKVIAISAGVSLAVHILVCWFFVYGYKLGIIGTM<br>ASVNVPWWLNIFILFLYSTRGGCTLTWTGFSSEAFTGLLELTKLSASSGIMLCLE<br>NWYYKILMLMTGNLVNAKIAVDSLSICMSVNGWEMMIPLAFFAGTGVRVANE<br>LGAGNGKGARFATIVSITLSLMIGLFFTVIIVIFHDQIGSIFSSSEAVLNAVDNLSV<br>LLAFTVLLNSVQPVLSGVAVGSGWQSYVAYINLGCYYLIGLPFGLTMGWIFKFG<br>VKGIWAGMIFGGTAIQTLILIIITTRCDWDNEAHKSSVRIKKWLVSDAGN
AT5G1<br>7700	MSGGGGEMEERLLNGSETEQRRESLYLRKKIWSEVRKMWRIALPSTLFRVMSFG<br>CVVVAQAFIGHSSETGLAAYALLQSTFIRFIYGIMAGMSSATETLCGQAYGAEQ<br>YHMMGIYLQRSWIVDTFIATLFVPFIVLAGPILRLLGQNVVISETVDEIYPWVIPY<br>LYSIVFTMTMQMYLQAQMKNAIIGILSTLALVLDIAATWWCVSVMGMGIHGA<br>LLGLNISSWSVAIAEFVYVFGGWCPHTWTGFSTAAFLDLIPMLKLSISSGFMLCL<br>EYWYMSIIVLMSGYAKDANIAISAFSICQYIYSWEMNICFGLMGAACVRVANEL<br>GKGDADAVRFSIKVVLVVSAVIGVICSALCLAFGGQISYLFSDSQAVSDAVADLS<br>IVLSISILFNIIQPILSGVAIGAGMQSMVALVNLASYYAIGVPLGVLLVYVFNFGIK<br>GLWSGMLAGVGIQTLILCYVIYKTDWELEVKKTNERMKTWTLNLPAVQSTTIST<br>RDEERK
AT5G1	METPNIISHTNLLSKIDLEKQNPAPIFPTITELKSEARSLFSLAFPTILAALILYARS

9700	AISMLFLGHIGELELAGGSLAIAFANITGYSVLAGLALGMDPLCSQAFGAGRPK<br>LLSLTLQRTVLFLLTSSVVIVALWLNLGKIMIYLHQDPSISSLAQTYILCSIPDLLT<br>NSFLHPLRIYLRAQGITSPLTLATLAGTIFHIPMNFFLVSYLGWGFMGVSMAAA<br>ASNLLVVIFLVAHVWIAGLHQPTWTRPSSECFKDWGPVVTLAIPSCIGVCLEW<br>WWYEIMTVLCGLLIDPSTPVASMGILIQTTSLLYIFPSSLGLAVSTRVGNELGSNR<br>PNKARLSAIVAVSFAGVMGLTASAFAWGVSDVWGWIFTNDVAIIKLTAAALPI<br>LGLCELGNCPQTVGCGVVRGTARPSMAANINLGAFYLVGTPVAVGLTFWAAY<br>GFCGLWVGLLAAQICCAAMMLYVVATTDWEKEAIRARKLTCTEGVDVVITTT<br>QTNGDLSEPLIYVVTVATD
AT5G38030	MEEDKILTETLLSAAEEPPALPFSSVEDIPPITTVGGFVKEFNVEVKKLWYLAGPA<br>IFMSITQYSLGAATQVFAGHISTIALAAVSVENSVIAGFSFGVMLGMGSALETLC<br>GQAFGAGKLSMLGVYLQRSWVILNVTAVILSLLYIFAAPILAFIGQTPAISSATGI<br>FSIYMIPQIFAYAVNYPTAKFLQSQSKIMVMAAISAVALVLHVLLTWFVIEGLQ<br>WGTAGLAVVLNASWWFIVVAQLVYIFSGTCGEAWSGFSWEAFHNLWSFVRLS<br>LASAVMLCLEVWYLMAVILFAGYLKNAEISVAALSICMNILGWTAMIAIGMNA<br>AVSVRVSNELGAKHPRTAKFSLLVAVITSTVIGLAISIALLIFRDKYPSLFVGDEEV<br>IIVVKDLTPILAVSIVINNVQPVLSGVAVGAGWQAVVAYVNIVCYYVFGIPFGLL<br>LGYKLNFGVMGIWCGMLTGTVVQTIVLTWMICRTNWDTEAAMAEGRIREWG<br>GEVSDQLLN
AT5G44050	MGERDDEAEGILEKAKIPLLKDQNVAEEENGEIKKEIWLETKKLWRIVGPAIFTR<br>VTTNLIFVITQAFAGHLGELELAAISIVNNVIIGFNYSLFIGMATALETLCGQAFG<br>AKKYDMFGVYLQRSWIVLFLFSILLLPMYIFATPILKFMGQPDDIAELSGIISVWA<br>IPTHFSFAFFFPINRFLQCQLKNSVIAISSGVSLVVHIFVCWLFVYVLELGVIGTIAT<br>ANVSWWLNVFILFTYTTCGGCPLTWTGFSMESFTRLWEFTKLSASSGIMVCLEN<br>WYYRMLIVMTGNLEDARIDVDSMSICMSINGLEMMVPLAFFAGTSVRVANELG<br>AGNGKRARFAMIISVTQSLIIGIIISVLIYFLLDQIGWMFSSSETVLKAVNNLSILLS<br>FAILLNSVQPVLSGVAVGSGWQSLVAFINLGCYYFIGLPLGIVMGWMFKFGVKG<br>IWAGMIFGGTMVQTLILIFITMRCDWEKEAQNAKVRVNKWSVSDARK
AT5G49130	MVVEEDSRLINLQHKYNPTMPEVVEELKRIWDISFPVAAMSILNYLKNMTSVVC<br>MGRLGSLELAGGALAIGFTNITGYSVLSGLATGMEPLCGQAIGSKNPSLASLTLK<br>RTIFLLLLLASLPISLLWLNLAPLMLMLRQQHDITRVASLYCSFSLPDLLANSFLHP<br>LRIYLRCKGTTWPLMWCTLVSVLLHLPITAFFTFYISLGVPGVAVSSFLTNFISLSL<br>LLCYIYLENNNNDKTTSKSLCLDTPLMLYGSRDSGENDVWSTLVKFAVPSCIAV<br>CLEWWWYEFMTVLAGYLPEPKVALAAAAIVIQTTSLMYTIPTALSAAVSTRVS<br>NELGAGRPEKAKTAATVAVGAAVAVSVFGLVGTTVGREAWGKVFTADKVVL<br>ELTAAVIPVIGACELANCPQTISCGILRGSARPGIGAKINFYAFYVVGAPVAVVL<br>AFVWGLGFMGLCYGLLGAQLACAISILTVVYNTDWNKESLKAHDLVGKNVIS<br>PNVDQIIVKCEEGLH
AT5G52050	MSQSNRVRDEVTLPLLQKTSHLKNHSSVLSVFLNEAISICKISYPLVLTGLFLYVR<br>SFVSLSFLGGLGDATLAGGSLAAAFANITGYSLFSGLTMGVESICSQAFGARRYN<br>YVCASVKRGIILLLVTSLPVTLLWMNMEKILLILKQDKKLASEAHIFLLYSVPDLV<br>AQSFLHPLRVYLRTQSKTLPLSICTVIASFLHLPITFFLVSYLGLGIKGIALSGVVSN<br>FNLVAFLFLYICFFEDKLSVNEDEKITEETCEDSVREWKKLLCLAIPSCISVCLEW<br>WCYEIMILLCGFLLDPKASVASMGILIQITSLVYIFPHSLSLGVSTRVGNELGSNQ

	PKRARRAAIVGLGLSIALGFTAFAFTVSVRNTWAMFFTDDKEIMKLTAMALPIV<br>GLCELGNCPQTTGCGVLRGSARPKIGANINGVAFYAVGIPVGAVLAFWFGFGF<br>KGLWLGMLAAQITCVIGMMAATCRTDWELEAERAKVLTTAVDCGSSDDDAK<br>EDMEAGMVDK
AT5G52450	MRDDRERGEGDLSWPLIGEKSSVKEEVKKQLWLSGPLIAVSLLQFCLQVISVMF<br>VGHLGSLPLSAASIATSFASVTGFSFLMGTASALDTLCGQAYGAKKYGMLGIQ<br>MQRAMFVLTLASIPLSIIWANTEHLLVFFGQNKSIATLAGSYAKFMIPSIFAYGLL<br>QCFNRFLQAQNNVFPVVFCSGVTTSLHVLLCWVLVFKSGLGFQGAALANSISY<br>WLNVVLLFCYVKFSPSCSLTWTGFSKEALRDILPFLRLAVPSALMVCLEMWSFE<br>LLVLLSGLLPNPVLETSVLSICLNTSGTMWMIPFGLSGAASTRISNELGAGNPKV<br>AKLAVRVVICIAVAESIVIGSVLILIRNIWGLAYSSELEVVSYVASMMPILALGNFL<br>DSLQCVLSGVARGCGWQKIGAIINLGSYYLVGVPSGLLLAFHFHVGGRGLWLG<br>IICALVVQVFGLGLVTIFTNWDEEAKKATNRIESSSSVKDFAVDDRSVVVF
AT5G65380	MRGGDGEEGSESRVALLKSPHTAEEDGEGLKDRILVETKKLWQIVGPAIFSRVT<br>TYSMLVITQAFAGHLGDLELAAISIVNNVTVGFNFGLLLGMASALETLCGQAFG<br>AKKYHMLGVYMQRSWIVLFFCCVLLLPTYIFTTPVLKFLGQPDDIAELSGVVAI<br>WVIPLHFAFTLSFPLQRFLQCQLKNRVTAYAAAVALVVHILVCWLFVDGLKLG<br>VVGTVATISISWWVNVLILLVYSTCGGCPLTWTGLSSEALTGLWEFLKLSASSGV<br>MLCLENWYYRILIIMTGNLQNARIAVDSLSICMAINGWEMMIPLAFFAGTGVR<br>VANELGAGNGKGARFATIVSVTQSLIIGLFFWVLIMLLHNQIAWIFSSSVAVLDA<br>VNKLSLLLAFTVLLNSVQPVLSGVAVGSGWQSYVAYINLGCYYCIGVPLGFLMG<br>WGFKLGVMGIWGGMIFGGTAVQTMILSFITMRCDWEKEAQKASARINKWSNT<br>IK
Glyma.01G007100.1.Wm82.a2.v1	MAQTYLLFLLPDLLINSFLHPIRVYLRAQNVTHPVTLASLAGTLLHVAFNLALV<br>ERGLGGVAAAAAASSFSILCLLVLYLWISGVHLATWTAPSRECLTCWEPLIRLA<br>APSCVSVCLEWWWYEIMILLCGLLVDPTASVAAMGILIQTTSLIYVFPSSLGLAV<br>STRVGNELGANRGRRARMSAVVAVFFAAVMGFSAVVFATAMRRRWGRMFTG<br>DEGILRLTGAALPILGLCELGNCPQTVGCGVVRGTARPNVAANVNLGAFYLVG<br>MPVAVGLAFWLEVGFCGLWLGLLSAQVCCAGLMLYMIGTTDWEYQACRAQL<br>LTALDEGSDGHKQPLIATLDNNNYS*
Glyma.01G025200.1.Wm82.a2.v1	MPSKEDSPLVEAKLPLLEAHPLTDEREQDQSFPRRFWLESKRLWHIVGPSIFSRIA<br>SYSMLVITQAFAGHLGDLELAAISIANNVVVGFDFGLLLGMASALETLCGQAFG<br>AKKYYMLGVYMQRSWIVLFICCIFLLPLYLFASPVLKLLGQPEELAELSGAVSIW<br>MIPVHFAFAFQFPLQRFLQCQLKTAPIAWVSLVALVVHVFVSWLFVFKLQFGV<br>VGAAATINFSWWVLTLGLFGYVVWGGCPHTWSGFSVEAFSGLWEFLKLSAAA<br>GVMLCLENWYYKILIVMTGNLENAEIAVDALSICMTINSLELMIPLAFFAATGV<br>RVANELGAGNGKGAKFATMVSVVTSVIIGLFFWMLILILHDKFGYIFSNSKAVL<br>DEVNNLSLLLAFTILLNSVQPVLSGVAVGSGWQSYVAYINLGCYYIIGVPLGILM<br>GWVFNQGVMGIWAGMIFGGTATQTLILSLITIRCDWDKEAERAKLHLTKWTD<br>PKQELN*
Glyma.01G026200.1.Wm82.	MKHSDGNSSTQPLLTARADEPHQIHPHPAETTAVFSAGTPDIAPITGAGDFYRE<br>FMVESKKLWYLAGPAIFSFVSKYSLGAFTQIFAGHVGTIDLAAVSVENSLIAGFS<br>YGIMLGMGSALETLCGQAVGAGKLDMLGVYMQRSWVLLLSTACVLCPLYIFA<br>GQVLKLIGQDTEISEAAGTFAIWMIPQLFAYALNFPVAKFLQAQSKVMVIAAIA

a2.v1	GMAMVLHPVLSWLLMVKLEWGLVGAAVVLNGSWWFVVVAQLVYVFGGWCWPAWNGFSWEAFRSLWGFFRLSLASAVMLCLETWYFMALILFAGYLKNAQVSVDAFSICMNILGWTIMVSFGMNAATSVRISNELGARHPRTALFSLVVAVITSVLIGVLLAIVLMISRNEYPSLFSNDTEVQDLVKDLTPFLCFCIVINNVQPVLSGVAIGAGWQALVAYVNIACYYLFGIPVGLVLGYKLDWGVKGIWLGMISGTILQTCVLLVLIYKTNWNEEASLAEDRIRTWGGHKKATVNDTENTQET*
Glyma.01G132200.1.Wm82.a2.v1	MCTGAESRGDINGDPVISKVGGDYEDRMRTIELEGGEETACNWGTFVRGLLQGIQHHLTALNLPSLSASELKEELQSLAKVACPIIMTSLMMYSRSAVSMLFLGRQGKVELAGGSLALGFANITANSVLKGLTMGMDPICCQAYGAKRWSVLNQTFLRTLCLLLLVAIPISILWLNMEPILQMLGQDPEVTKVAQVYMVFSIPELLAQAHLNPLRSFLRTQGLTTPVTIAASCAALLHLPINYFLATYLNLGVKGIALATGLNSINMTLGLLLYILFSKKPLKPWQGATLLSAFHGWKPLLSLALPSCISVCLEWWWYEIMLFLCGLLSNPQATVATMGILIQTLGFLYVFPFSLSIALTTRIGHSLGAGQASKAQSTAIIGFLTAFTLGLTAFILLFLVRKSWGKLFTNETQIVELVTTILPILGLCEVSNWPQTVSCGILSGTARPYLGARINLCAFYLVGLPVSVFATFIYKYELVGLWSGMVAAQASCLCMMVYTLIQTDWEQQCKRAVELAQKTTERENKNDDEESGLLGSDQ*
Glyma.01G213000.1.Wm82.a2.v1	MSATLTCKLGDNNLSLNDKNDQPPSPPQPLQKCPANMVSMVVSELRIQRGIALPMVAMNLAWFAKTAITTAFLGRLGELSLAGGALGFTFANVTGFSVLNGLCGAMEPICGQAHGAKNFRLLHKTLLMAISLLLLVSLPITFLWLNVDKILILFGQQQDISTVARTYVSCLIPDLFVASLFCPLKAYLSCQSITLPTMFSSAVALAFHIPINIVLSRTMGLRGVSMAVWITDLIVVVLLAIYVLILENKKESMWKEGGWWDQSIEDWIRLLKLCGSCCLNTCLEWWCYEILVLLTGHLTNAKQAVGVLAIVLNFDYLLFSVMLSLATCVSTRVSNELGANQAGLAYRSACVSLALGFISGCIGSLVMVAARGIWGPLFSHDMAIIKGVKKTMLLMALVEVFNFPLAVCGGIVRGTARPWLGMYANLGGFYFLALPLGVVFAFKLCLGLVGLLIGLLTGIVTCLTLLLVFIARLNWVEEAAKAQTLTGQEQVKELSKYDAEERIDAHEKDVV*
Glyma.01G216600.1.Wm82.a2.v1	MCQLSSPSTLCESNEGHPNMPPTKIHEEPDMFVLLIPKPPTTTTFFEQGQNNQKPHKTHFSLALDEVKCIANIALPMVLTGLLLYSRSVISMLFLGRVGELALAGGSLAIGFANITGYSILSGLAMGMEPICGQAFGAKRFKLLGLAMQRTMVLLLLTCVFISFLWFNMKKILVLCGQQEDIATEAQSFILFSIPDLVAQSLLHPLRIYLRSQSITLPLTYTASLSILLHVPINYFLVSVLKLGIKGIALGAVWTNFNLVFSLILYIWVSGVYKKTWPGVSLKGVFSGWKSLLNLAIPSCISVCLEWWWYEIMILLCGLLINPQATVASMGVLIQTTALIYIFPSSLSFAVSTRVGNELGAENPKKAKLAALVGLCFSYGLGFSALFFAVSVRHVWASMFTSDAEIIALTSMVLPIIGLCELGNCPQTTVCGVLRGTARPKLGANINLGCFYLVGMPVAVRLSFFAGFDFKGLWLGLLAAQASCMFTMLIVLARTNWEGQVQRAKELTSSSDSSEEEEEQDQKCLFSSGATKECSHSDSLV*
Glyma.02G038600.1.Wm82.a2.v1	MPRSRRSNPRLAERVFIAGTPDIAPITSTGDFYREFMVESKKLGYLAGPAIFSFVSKYSLGAFTQIFAGHVGTIDLAAVSVENSLIAGFSYGIMLGMGSALETLCGQAVGAGKLDMLGVYMQRSWVLLLSMAFVLWPMYIFAGQVLKFIGQDTQISEAAGTFAIWMIPQLFAYALNFPVAKFLQAQVLSWLLMVKLELGLVGAAVLVYVFGGWCWPAWNGFSGEAFRNLCGFFRLSLASAVMLCVNNEVFKIDNFSPLVTSLTLLLDYIVECTALDGFDALKKKQVQGILVGLIPLGNMYFMALILFAGYLKNAQVFVDAFSICMNILGWTIMVSFGMNAATRATLFSLVVAVITSVLIGILLAIVLMILRNEYPSLFSNDTEGQDLVKNLTPFLCFCIVINNVQPVLSGVAIGAGWQALVAYVNIACYY

	LFGIPVGLVLGYKLDWGVKGIWLGMIAGTILQTCVLLVLIYKTNWNEEASLAED<br>RIRTWGGHEKATVKDTKNTKEET*
Glyma.02G039500.1.Wm82.a2.v1	MSKMSSDEALALEEVHHPLLEDYCADGEEKEYFVRRVWNESKKLWNISGPAIF<br>NRVATFSMFVITQAFAGHLGDLELAATSIAINVILGLDFGILLGMSSALDTLCGQ<br>AFGAKKYYMLGIYMQRSWVVLSITGVMFLALFLFVTPILKFFGQTSEIAELAGVI<br>SLWLIPTHLAYIFYLPMHFFLQSQLKNNVTTWVSLLGLLVHAYLCWLVVNKFH<br>LGVIALVAFGNIAWWLLVLGYFGYVICGGCTLTWTGFSIEAFSGVWEFSKLSTAS<br>GIMICLEVWYDKALMLMTGNLQSAKTTIEALTICLTINIWELMFPLSFYAATAV<br>RVANELGAGNGKGAKFASMVSVVTSIIISIFFWLLIMVFRRKLAYLFSSSEVVIKE<br>VDKLSPFLGVTILLNSVQPVLSGVAVGSGWQKYVAFINLGSYYLIGLPLGYLLGF<br>VFRLGVQGVWAGLIFGGPAIQTLILAWVTSRCNWDKQAERARLHLTKWDPNQ<br>ELIR*
Glyma.02G075300.1.Wm82.a2.v1	MGDNKDRDFFSHKFPTTSQVMEEMKELWGMALPITAMNMLVFVRAVVSVLFL<br>GRLGSLELAGGALSIGFTNITGYSVLVGLAAGLEPVCSQAYGSKNWDLLSLSLQ<br>RMVLILLMAIVPISLLWLNLERIMLFMGQDSAITGMASLYCFYSLPDLLTNTLLQ<br>PLRVFLRSQKVTKPMMYCSLVAVLFHVPLNYLLVVVMGLGVPGVAMASVMT<br>NLNMVVLMAGYVCVCRKREVVVKWGCWGVGGGVVCSGLGQLMGFAVPSCL<br>MICLEWWWYEIVTVLAGYLPRPTLAVAATGILIQTTSMMYTVPMALAGCVSAR<br>VGNELGAGKPYKAKLAAVVALGCAFVIGFINVTWTVILGQRWAGLFTNDEPV<br>KALVASVMPIMGLCELGNCPQTTGCGILRGMARPGIGAHINLGSFYFVGTPVA<br>VGLAFWFKVGFSGLWFGLLSAQVACAVSILYVVLVRTDWEAEALKAEKLTRIE<br>MGSCNGLRNKENERDEERKGLLVNGNGNNKDDIC*
Glyma.02G089900.1.Wm82.a2.v1	MEETLLPKENKRVTLTNSKSSSGFVQELKNVSLMAAPMVVVSVSQFLLQVVSL<br>MMAGHLGELSLAGVALATSFADVTGFSILMGMAGALETQCGQSFGAEQFHKL<br>GNYVFCAILSLILSSVPISIIWIFMDKLLILLGQDHAISLIAGNYCIWLIPALFGYAV<br>LQALVRYFQTQSLIFPMLVTSVVVLVLHIPICWVLVFGLGLGQNGAAISIGISYW<br>LSVMLLLIYTKYYPSCQKTKIALGSNALRSIKEFFFLAIPSALMICFEWWSFELVVI<br>LAGLLPNPKLETSVLSICLNICTLHYFIPYGTGAAVSTRVSNELGARRPQAAREA<br>VFAVIVLAFTDAVVFSSVLFCFRHVLGFAFSNEMEVVHYVAKIVPVLCLSFMVD<br>GFLGVLCGIVRGSGWQKIGAITNLVAYYAVGIPVSLLFGFGLNFNGKGLWIGILT<br>GSTLQTIILALLTAFTNWEKQASLAIERLSEPDETVF*
Glyma.02G090000.1.Wm82.a2.v1	MMGKEEATPLLRKSEVAPLEDDDAFCVELKRVGSMAAPMVAANMCQYLLQV<br>VSLMMVGHLGVLVSFSGVAIATSFAEVTGFCVLMGMAGALETLCGQTYGAEEF<br>SEIGNYTFCAIVTLLLVCLPISMLWIFVDKILLLFGQDPEISHVAHEYCIYSIPALY<br>GFAVLQCQIRYFQTQSMIFPMVFSSIAVLCLHVPICWALVFKLALGHVGAAYAI<br>GISYWLNVIGLGIYMNFSPACEKTKIVFSFNALLSIPEFCQFAIPSGLMFCFEMWS<br>FELLTLFAGLLPNPQLQTSVLSVCLNTTTLHYIIPYAVGASASTRISNELGAGNPK<br>AAQGIVRVIVIIGIVDGVIVSIFFVCCRHILGYAYSNDKEVVDYVSDIVPILCGSFT<br>ADSLIGALSGIARGGGFQQIGAYVNLGAYYLVGVPLAFLLGFVLHFNAKGLWM<br>GSLTGSVLQVIILTVVTVLTDWQKEATKARVRIVEKSIKAHNGSV*
Glyma.02G090100.1.Wm82.	MEESSTGKWGWMKMREELKKVGTIAAPMAVSSVLQYLLPVVSLVMVGHLNQ<br>LSLSSVAIATSLTNVSGFSVLSGMAGGLETLCGQAFGAGQYEKFGLYTYTAIISLS<br>LVCFPITILWIFNDKILTLLGQDPTISLEVRKYAIWLIPALFGSAILKPLTRFFQTQS<br>LISPMILTSAIALCFHVVTCWTLVFKLGLGHVGAAISFSLCVWFNVIMLLSFVRYS

a2.v1	SACEKTRISFSKNALVGVGEFFRFAVPAAVMVCLKWWACEILVLLAGLFPNPKLETSVLSICLTISTLHFTIPYGFGAAASTRVSNELGAGNPQAVHVAVSATMFLAVTEGFIVSATLFGCRHILGYAYSDDRMVVHYVAVMIPLLCLSIFTDSLQGVLSGVARGSGWQHLGAYVNLGAFYLVGIPVGILLGFVAHFRAKGLWIGIVTGSIVQSILLSLITALTNWKKQAIMARERVFDAKPPDVNGSYHMTSA*
Glyma.02G181800.1.Wm82.a2.v1	MMPLLMLFSGIRNAFSSDELGLEILNIALPTTLALAADPIASLIDTAFIGHIGPVELAAVGVSIAIFNQISKITIIPLVSVTTSLVAEEDAADEQNQQSEKEMLMKVSNEDVKLDVHDHIEKAGNPSSANVGRVAKLKHDKSYIPSASSGVVIGGVLGVLQALFLIFTAKPMLSYMGVDSNSPMFKPAQQYLTLRSFGAPAVIISMAIQGVFRGIKDTKTPLYATVMGDVTNIILDPLLMFVLRLGVNGAAISHIISQYLISIMLLWSLMQQVVLIPPSIQDFQFGKILKNGFLLLIKVASVTFCVTLSASLAARKGSTTMAAFQICLQIWMATSLLADGLAVAGQAIIASAFARNDYKRVIASASRVLQLGLILGLVLSVLLLSLLPFASRLFTNDNNVLQLISIGIPYVAATQPINALAFVFDGVNYGASDFTYSAYSMIMVALVSILSLYTLSSSLGFTGIWIALSIYMTLRIFAGFWRIGTGSGPWSFLKVNNVGL*
Glyma.02G218400.1.Wm82.a2.v1	MEEIKAIGRISCPTAITGLILYSRAMISMIFLGYLGEMELAGGSLSIGFANITGYSVISGLAMGMEPICGQAYGAKQWKILGLTLQRTVLLLLSTSIPISFMWLNMKRILLWSGQDQEIASVAQTFITFSIPDLFLLSLLHPLRIYLRTQSITLPLTYCSAISVLLHVPLNFLLVVHLKMGIAGVATAMVLTNLNLILFISSFVYFSGAYKASWVSPSVDCIKGWSSLLSLAIPTCVSVCLEWWWYEFMIMLCGLLVNPKATIASMGILIQTTSLVYVFPSSLSLGVSTRVGNELGAKNPRKARVSMIVSLFCALALGLAAMLFTTLMRHQWGRFFTNDHEILELTSLVLPIAGLCELGNCPQTTGCGVLRGSARPTIGANINLGSFYLVGMPVAILLSFVAKMGFPGLWLGLLAAQASCAGLMFYVLCTTDWNVQVERAKELTKSSTTTTTAMTATIATTSCSTSPNCNYKLLPTLTKKEANMNKNAHVCLEEIVITSGGGGDELTKISSVETDPLIIPTTKHTQV*
Glyma.02G310800.1.Wm82.a2.v1	MVMEATHFTTSQYQYHIDGGFTRRPFMRMRKRSHISYGYGPHFLRRSLYVPFIALGPKAKAKAEAEAEAEASSFSSVDVRRELISLTLPALASQAIDPLAQLMETAYIGRLGTVELASAGVSISIFNIISKLFNIPLLSVATSFVAEDIAKASSTADAKTKQQLSSVSTALLLALVLGFFEALALYLGSGAFLHLIGVSTQNPTYVPARHFLSLRAVGAPAVVLSLSLQGIFRGFKDTKTPVICLGIGNFSAVFLFPLLMYYFRLGVTGAAISTVISQYIGTMLMIWCLNKRAELLPPKMGDLQFGSYIKSGGFLLGRTLSVLSTMTLGTSMAARHGPVAMAAHQICMQVWLAVSLLTDALAASGQALIASSVSRHEYKVAKEVTSLVLRIGLVMGICLTAILGASFGSLATIFTQDTEVLQVIRTLALFVSASQPFNALAYIFDGLHYGVSDFRYAAFSMMFVGAVSSAFLVFAPPLFGLQGVWLGLGLFMALRAAAGAVRLLSKNGPWWFLHRFTNCRGGLISL*
Glyma.03G005200.1.Wm82.a2.v1	MEGNLKQKLLSREKISEEEENLSLVKRVWEESKVMWIVAAPAIFTRFTTFGLSVISQAFIGHIGSKELAAYALVFTVIIRFANGILLGMSSALSTLCGQAYGAKEYDMMGVYLQRSSIVLFLTALCLLPLFIFTSPILTLLGQDESIARVARNVSLWSIPILFAYIVSFNCQTFLQSQSKNVIAFLATLSIIIHVSLSWLFTIQFKYGIPGAMISTILAYWIPNVGQLIFITCGWCPETWKGFSSLAFKDLWPVVKLSLSAGAMLCLELWYNTILILLTGNMKNAEVQIDALSICININGWEMMIAFGFMAAASVRVANELGRGSSKDAKFSIVVTVLTSFSIGFILFVLFLFLREKVAYLFTSNEDVATAVGDLSPLLAVSLLLNSIQPVLSGVAVGAGWQSIVAYVNIGCYYLIGIPVGIVLGNIIHLQVKGIWIGMLFGTLIQTIVLTIITYKTNWDEQVIIARNRISKWSKVDLDRETVTSDN*

Glyma.<br>03G005<br>300.1.<br>Wm82.<br>a2.v1	MEGNLEKKLLSREQKSEEENLSLVKRVWEESKVMWIVAAPAIFTRFTTFGISVIS<br>QAFIGHIGSRELAAYALVFTVIIRFANGILLGMASALSTLCGQAYGAKEYDMMG<br>VYLQRSWIVLFLSAICLLPLFIFTSPILTLLGQDESIAQVARTISIWSIPVLFAYIVSN<br>SCQTFLQSQSKNVIISYLAALSIIIHVSLSWLFTMQFKYGIPGAMISTILAYWIPNIG<br>QLIFITCGWCPETWKGFSFLAFKDLWPVAKLSISSGAMLCLELWYSTILILLTGN<br>MKDAEVQIDALSICINISGWEMMIAFGFMAAVSVRVANELGRENSKAAKFSIV<br>VTVLTSFAIGFILFVLFLILREKVAYLFTSNEDVATAVGDLSPLLALSLLLNSIQPV<br>LSGVAVGAGWQSTVAYVNIGCYYLIGIPVGIVLGNIIHLQVKGIWIGMLFGTLIQ<br>TIILIIITYKTNWDEQVIIARDRINKWSKMVLDHETITSDN*
Glyma.<br>03G005<br>400.1.<br>Wm82.<br>a2.v1	MEGNLEKKLLSKEEVSEEDNLSLVKRVWEESKEMWIVAAPAIFTRFTTFGINVIS<br>QAFIGHIGSRELAAYALVFTVIIRFANGILLGMSSALSTLCGQAYGAKEYDMMG<br>VYLQRSSIVLFLTALCLLPVFIFTSPILMLLGQDENIAQVAGTISLWSIPILFAYIVSF<br>NCQTFLQSQSKNVVIAFLAALSIIIHVFLSWLLTIQFKFGIPGAMISTILAFWIPNIG<br>QLIFITCGWCDETWKGFSFLAFKDLGPVVKLSLSSGAMLCLELWYNTVLILLTG<br>NMKNAEVEINALSICININGWEMMIALGFMAAASVRVANELGRGSSQAAKFSI<br>VVSVLTSFVIGFILFVLFLFLREKIAYLFTSNEDVVTAVGDLSPLLALSLLLNSIQPV<br>LSGVAVGAGWQSTVAYVNIGCYYLIGIPVGIVLGNIIHLEVKGIWIGMLFGTLVQ<br>TIVLTIITYKTNWDEQVTIARNRISKWYKVELDHETSTSDN*
Glyma.<br>03G005<br>500.1.<br>Wm82.<br>a2.v1	MWVVAAPAIFTRFSTFGINVITHAFVGHIGSRELAAFALVFTVLIRFGNSILLGM<br>GTALSTLCGQAYGAKEYGMMGVYIQRSWIVLSLTALCLLPLLIFAIPILTLLDQD<br>ETIAQVAGTISLWSIPVLFSFIVSFTTQTFLQSQSKNIIIAFLAAFSIVIHVFLSWLLT<br>MKFKLGIAGAMTSTSLALWIPNIGQLIFITCGWCYDTSKWKGFSFLAFKDLWPV<br>VKLSLSSLPTNGLELWYSTILILLTGNMEDAEVQIDALSICLNINGWELMISLGFM<br>AAASVRVAKGSSKAAKFSIVVKVLTSFAIGFILFFIFLFLKEKLAYIFTSSKDVADA<br>VGDLSPLLAISILLNSVQPVLSGIPVGVVLGNVLHLQVKGIWFGMLFGTFIQTIVL<br>IIITYKTNWDEQVIIARNHINKWSKVDSHHKIIPSEN*
Glyma.<br>03G005<br>600.1.<br>Wm82.<br>a2.v1	MQGGVKEKLLIKEKKTSEQEEEELSLVKRVWNESKVMWVVAAPGIFTRFSTFGI<br>NVISQAFIGHIGSRELAAYALVFTVLIRFANGILLGMASALSTLCGQAYGAKEYV<br>MMGVHLQRSWIVMSITSLFLLPVFIFTRPILMLLGQDENIAEVAGNISLWSIPMIF<br>AFIASFTCQNFLQSQSKNTIISFLAAFSIVIHLFLSWLLTIQFKLEIPGAMTSTNLAF<br>WIPNIGQLIFITCGWCSDTWKGFSFLAFKDLWPVVKLSLSSGIMLCLELWYNTIL<br>VLLTGNMENAEVQIDALSICLNINGWEMMISLGFMAAASVRVANELGKGSSK<br>AAKFSIVVTVLTSLAIGFVLFLFFLFLRGKLAYIFTSNKDVADAVGDLSPLLAISIL<br>LNSVQPVLSGVAIGAGWQSIVAYVNIGCYYIIGIPVGVVLGNVLNLQVKGIWIG<br>MLFGTFIQTVVLTVITYKTDWDEQVTKARNRINKWSKVESDHETITSDD*
Glyma.<br>03G005<br>800.1.<br>Wm82.<br>a2.v1	MEGDLKQKLLRRRKEEEEEEEEELSLAKRVWNESKVMWIVAAPAIFTRFSTFGIS<br>VISQAFVGHIGSKELAAYALVFTVLIRFANGVLLGMASALSTLCGQAYGAKEYG<br>MMGVYLQRSWIVLFLTAVCLLPVFIFTSPILLLLGQDESIAQVAGNIALWSIPVM<br>FAFIVSFTCQTFLQSQSKNIIIAFLAAFSIVIHVFLSWLLTMKFKFGIPGAMISAGL<br>AYWIPNIGQLIFVTCGWCSDTWKGFTFLAFKDLWPVVKMSLSAGAMLCLELW<br>YNTILVLLTGNMKNAEVEIDALSICLNINGWEMMISLGFMAAASVRVANELGR<br>GSAKAAKFSIIVSVLTSLAIGFLLFIFFLFFRERLAYIFTSNKEVAFAVGDLSPLLSVS<br>ILLNSVQPVLSGVAIGAGWQSIVAYVNMGCYYAIGIPVGIVLGNVLDLQVKGIW<br>IGMLFGTLIQTIVLIVITYKTNWDEQVTIAQKRISRWSKVDSPDHENEVERKNVS*

Glyma. 03G036 200.1. Wm82. a2.v1	MCTGAESRGDINGDPVISKVGGGDYEDRMRTIELEGGGEERACNCGTFVQGLL<br>QGIHHHLTALNLPTLSASELKEELQSLAKVACPIIMTSLMMYSRSAVSMLFLGR<br>QGKVELAGGSLALGFANITANSVLKGLTMGMDPICCQAYGAKRWSVLNQTFL<br>RTLCLLLLVAIPISVLWLNMEPILQMLGQDPEVTKVAQVYMVFSIPELLAQAHL<br>NPLRSFLRTQGLTTPVTIAASCAALLHLPINYFLATYLNLGVKGIALATGLNSIN<br>MTLGLLLYILFSKKPLKPWQGATLLSAFHGWKPLLSLALPSCISVCLEWWWYEI<br>MLFLCGLLSNPQATVATMGILIQTLGFLYVFPFSLSIALTTRIGHSLGAGQASKA<br>QSTAMIGFFTAFTLGITAFILLFFVRKSWGKLFTNETQIIELVTTILPILGLCEVSNW<br>PQTVSCGILSGTARPYLGARINLCAFYLVGLPVSVFATFIYKYELVGLWSGMVAA<br>QASCLCMMVYTLIQTDWGQQCKRALELAQKATEQENKNDEESGLLGSDQ*
Glyma. 04G089 000.1. Wm82. a2.v1	MCNPKPSSSSPFLCPTKNHIITPDPKLINHPPPVDDQLKDELHRWPTPNEVIAEM<br>KAIGKISGPTALTGLILYSRAMISMVFLGYLGEMELAGGSLSIGFANITGYSVISGL<br>AMGMEPICGQAYGAKQLKTLGLTLQRTVLLLLSTSLPISLTWLNMKNILLWCG<br>QDQQISSTAQTFIIFSIPDLFLLSLLHPLRIYLRTQSITLPLTYCSAISVLLHVPLNFL<br>LVVHFKMGVSGVAIAMVWTNLNLFIFLSSFVYFSRVYKDSWVPPSTDCLRGWSS<br>LLALAVPNCVSVCLEWWWYELMIILCGLLLNPKSTIASMGILIQTTALVYVFPSS<br>LSLAVSTRVGNELGANRPAKARISMIVSLACAVALGVAAMLFTTLMRHRLGRF<br>FTSDREILHLTSIALPIVGLCELGNCPQTTGCGVLRGSARPTVGANINLGSFYLVG<br>MPVAVLLGFVGKMGFPGLWLGLLAAQASCASLMIFVLCTTDWNAQVRRANE<br>LTNANSAPFKQTEYCNNVRLREIVATEDHDHEITNRPSHYTHYK*
Glyma. 04G097 700.1. Wm82. a2.v1	MDTSCQQKGFFTFLPQNITKAPHYCCPVIDNLRSEAKHMGNILNEGEHEHPLIK<br>SKLPPQPHGSNNHSLFQRSCSESKKLWHIAAPSIFTRLAMFSITVVTQSLAGHLG<br>DLDLAAISIACTVLISITFGFLLGMASALETLCGQAYGAGQQRILGVYLQRSWVV<br>LFLSSILLLPVFIFATPVLKLIGQPVAVAEQAGLVAVWLIPLHLSFPFQFTLQRFLQ<br>CQLKTGIIAWVSGVALAVHVLVSWVFVYRMRIGIVGTALSIGFSWWLSVLGML<br>GYTLFGGCPRSWTGFSVEAFVGLWEFFKLSLASGVMLALENFYYRLLLIVSGYM<br>HNTEIAIDALSVCVTIYGWESMIPLAFLGATGVRVANELGAGNAKGARFATVV<br>SVVTTLFVGFIFWLVIVSFNKNLALIFTSSSSVIQMVNELAMLLAFTVLLNCIQPV<br>LSGVAVGSGRQAVVAYINIGSYYLIGIPLGVLLGWLLPSGIGMWTGMMSGTVV<br>QTLILAIITMRYDWEKEVQKAQILVEEEATFNDQ*
Glyma. 04G097 900.1. Wm82. a2.v1	MGSLKGDSGHANGENLTEALLPTRDAQQQHQTDDEEQRFGDKLWLETRKLW<br>LIVGPSIFSRLASFTMNVVTQAFAGHLGDVELAAISIANNVLVGFNFGLLLGMA<br>SALETLCGQAFGAKRYHLLGIYMQRSWIVLFMCCFLLLPFYVFATPLLKFLGQP<br>DDVAEWSGVVAVWLIPLHFSFAFQFPMQRFLQCQLKTAVIAWVSLLGLVVNV<br>VTSWLFIYVWDFGLYGAAISLDISWWVLVFGMYAYIAYGGCPLTWNGFSLEAFS<br>GLWEFLTLSSASGVMLCLENWYYKILLLMTGQLENATIAVDALSVCMTINGWE<br>MMIPLAFFAGTGVRVANELGAGNGKAAKFATQVSVAQSTIIGLIFCVLIMIFHE<br>HIAYIFTTSTSVLQAVDNMSLLLAITILLNSVQPVLSGVAVGSGWQAYVAYINIG<br>CYYLIGFPLGIIMGWVFKSGVIGIWGGMIFGGTAIQTLILIIVTIRCDWEKEEEKAC<br>FRVSKWSKSNSNGNLEASN*
Glyma. 04G102 100.1. Wm82.	MLITSCVTLCVRIPLCWVLVFKTRQNNVGGALAMSISIWSNVFFHGLYMRYSPT<br>CAKTGAPIFMELFQRLWEFFRFAIPSAVMICLEWWPFELIILLSGLLLNPQLETSV<br>LSVCTRISNELGVGNPRGARVSVRAAMPFAVVETTIVSGTLFACRHVFGYIFSNE<br>KEVVDSVTLMAPLVCIWVILDNIQGVLAGVARGC*

a2.v1	
Glyma.05G001700.1.Wm82.a2.v1	MESSRELAAPLLVLRKSGEQENNNGVESTFCQEFKRVSSMAAPMVAVTVSQYL<br>LQVVSLMMVGHLGILVSFSGVAIATSFAEVTGFSVLLGMSGALETLCGQTYGAE<br>EYRKFGNYIWCAIVTLTLVCLPISLVWIFTDKILMLFSQDPEISHAAREYCIYLIPA<br>LFGHAVLQALTRYFQTQSMIFPMVFSSITALCLHVPICWGLVFKLGLRHIGAAL<br>AIGVSYWLNVVWLAIYMIFSPACQKTKIVFSSNALLSIPEFLKLAIPSGLMFCFEW<br>WSFEVLTLLAGILPNPQLETAVLSVCLNTTTLHYFIPYAVGASASTRVSNELGAG<br>NPKTAKGAVRVVVILGVAEAVIVSSVFISCRHVLGYAYSNDKEVIDYVAEMAPL<br>LCVSVTADSLIGALSGIARGGGFQEIGAYVNLGAYYLVGIPMGLLLGFHLQLRA<br>KGLWMGTLSGSLTQVIILAIVTALTDWHKEATKARERVVENSIKVHYEA*
Glyma.05G049700.1.Wm82.a2.v1	MRQLSPTSNPKVQDEPNMLATLIPKSPTCQQQQQMQNHHNNNNNTTHLSLS<br>LNEAKCIANISFSMVLTGLLLYSRSMISMLFLGHLGELALAGGSLAIGFANITGYS<br>VLSGLAMGMEPICGQAFGARRFKLLGLTMQRTVLLLLVTSCLISLFFWLNMRKI<br>LLLCGQEEDIANEAELYILYSLPDLVLQSLLHPLRIYLRSQSITLPLTCCAAVSILL<br>HVPINYLFVSILKLGIKGVALSAVVTNLNLVWLLIVYVVVSGTHKKTWPGISREC<br>FQGWNSWKTLMNLAIPSCVSVCLEWWWYEIMILLCGLLVNPHASVASMGVLI<br>QTTALIYIFPSSLSFGVSTRVGNELGAGNPRRAKLAAMVGLCFSFVFGLSALAFA<br>VSVRNVWASMFTLDGQIIALTSAVLPIIGLCELGNCPQTTVCGVLRGTARPKLG<br>ANINLGCFYLVGMPVAVWLGFFAGFDFKGLWLGMLAAQGSCMMTMMFVLA<br>RTNWEGQALRAKELTDSDSGEEKNLMEEEEQEEEGFLGSCATKEGSV*
Glyma.05G054000.1.Wm82.a2.v1	MSATSKEGENLSTNDRANRHPSTPSSHTKKCPLNANFVKMVVEELRVQRGIAL<br>PLVAMNLAWFAKLAITTAFLGHLGELNLAGGALGFSFANVSGFAVLNGLSGA<br>MEPICGQAHGAKNVRLLHKTLLMTTLLLLLVTLPLSFLWLNVDKILILFGQQQE<br>ISIVAKTYVSYLIPDLFIKALLCPLKAYLSSQCMTLPTMFSSAVALAFHIPVNILLS<br>KTMGLRGVSIAVWVTDLIVMVMLAIYVVVLERRNGSMLWKEGGWWDQNMM<br>DWSRLIKLSGSCCLNTCLEWWCYEILLFLTGHLANAKQAVGVLAIVLNFDYLL<br>YAVMLSLATSVSTRVSNELGANRAGQAYKSARVSLAVSVISGCIGGSIMVAARG<br>VWGNLFSHQKGVVKGVEKAMLLMALVEVFNFPVTVCGGIVRGTGRPWLSMY<br>ASLGGFYFLTLPLGVVFAFKLRFGLGGLLIGLLIGIAACLVLLLTFIVRINWVEEA<br>TKAQTFVCNAQVHEQVSRNEVNELTENPEGDQVCKETSITPYSCI*
Glyma.05G054100.1.Wm82.a2.v1	MNLARFAKTAITTAFLGHLGELNLAGGALGFSFANVTGFSVLNGLRGAMETK<br>NVRLLHKTLLMTTLLLLLVTLPLSFLWLKVDKILIRFGQQQEISIVAKTYVSYLTP<br>DLLVTSLLCPLKAYLSSQCMTLPTMFSSAVTLAFHIPVNILLSKTMRLRGVSIAV<br>WINDLMVMVMLAIYVVILERRNGSMLWKEGGWWDQNMMDWIRLIKLSGSC<br>CLNTCLEWWCYEILVLLTGHLANAKQAVGVLALVLNFDYLLYSVMLSLATCVS<br>TRSAVSVISVCIGGVVKGLKKTMLVIALVEVFNFPVTVCGGMVRGTARP*
Glyma.05G226400.1.Wm82.a2.v1	MEGAAEKERLQCWSIRREVKAVVELAFPIGITALIFYARSMVSMLFLGHLGELEL<br>AAGSLGMAFANITGYSVLSGLALGMEPMCSQAFGAKRVKVLSLTLHRCVMFLL<br>VCSIPISLLWLNMSSILLLLHQDPNITLMAHTYLIFSLPDLLTHSFLHPIRIYLRAQ<br>GVTHPVTLASLAGTLLHLPFNYLLVTRLRLGLAGVAAASAASNLSILLFLGAAV<br>CFTGLHCAAPSRDCFSGWKPLLRLAAPSCVSVCLEWWWYEIMIILCGILVDPTA<br>TVASMGILIQTTSLIYVFPSSLGFAVSTRVGNELGANRPSRAKLSAVVSVFLAAIM<br>GFSAMFFAVGMRRRWGRMFTADEDIIRITSMALPILGICELGNCPQTVGCGIVR<br>GMARPNTAANVNLGAFYLVGMPVAVGLGFWFDVGFCGLWLGLLSAQVCCA

	GLMLYVIGTTDWEFEAHRAQWLTLVEDGVMDGQKQPLTSVVTEAPST*
Glyma.05G243100.1.Wm82.a2.v1	MRIVREEVVEEVKRLWLAVPLFSVGILLHILQAISIMFVGHLGTLPLSGASMASSFASVTGFNLLPFYLFASSKLVIGVSYCTGHILWSIKWSRTVPYAWHTHAEIHACCFNDMIPSLFAYGILRCILKFLQTQKIVFPMVLTSGIAAVLHVLFCWLLVFKSGLANRGAALANSISYWVNAILISLYVRFSSACKHSWTGFSKMALHNLLDFLKLEWTFKLMVLMSGLLPNPKLETSVFSICLNTFGLGWMIPFGFSAAIIETEYAIYSTHVCASFVRCQVVSYIRFIVTIESTKSLMSTATINIKTPKEKEKKIIQPIKIQFTYLSSPKVYFFSSAIYSNCSRKCPHTMFSMNSSILVSENVKNKNVQGGAE*
Glyma.06G091100.1.Wm82.a2.v1	MCNPKPSSSSPFLCPTKNHIISTPDPKLINHPPPVDDQLQDELHRWPTPNEIVAEMKAIGKISGPTALTGLIIYSRAMISMVFLGYLGEMELAGGSLSIGFANITGYSVISGLAMGMEPICGQAYGAKQLKTLGLTLQRTVLLLLSSSLPISLTWLNMKNILLWCGQDHEISSTAQTFILFSIPDLFLLSLLHPLRIYLRTQSITLPLTYCSAVSVLLHVPLNFLLVVHLKMGVSGVAIAMVWTNLNLFLFLSSFIYFSGVYKDSWVPPSTDCLRGWSSLLALSVPTCVSVCLEWWWYELMIILCGLLLNPKATIASMGILIQTTSLVYVFPSSLSLAVSTRVGNELGANRPAKARISMIVSLACAVALGVTAMLFTTLMRHRWGRFFTSDQQILHLTSIALPIVGLCELGNCPQTTGCGVLRGSARPTVGANINLGSFYLVGMPVAVLLGFVGKMGFPGLWLGLLAAQGSCAALMIFVLCTTDWNAQVQRANELTNANSAPSKLPTTECNNVRLPEILVMDTEDHHDEIITKTPSLETDPLIIHTSK*
Glyma.06G099600.1.Wm82.a2.v1	MASALETLCGQAYCAGHHRMLGVYLQRSWVVPVFHSDVAGVHFCHARVEVNRETHCGGGTGGFSGALVDPFHLSFPFQFTLQRFLQCQLKTGIIAWVSGWLL*
Glyma.06G103400.1.Wm82.a2.v1	MEENLLVLAKGSGEEQKVAWEGLGEEMKRMIDIAGPMVVVTASQRLLQVVSVMMVGHLNDDLFLSSAALAISLTAVTGFSFLMGMASGLETICGQAYGAQQHKKIGVQTYTAIFALTFVCLPFTFLWINMEKILVFIGQDPLIAKEAGKFIIWLIPALFAYAILQPLVRYFQMQSLLLPMLMTSCVTLCVHIPLCWVLVFKTRLNNVGGALAMSISTWSNVIFLGLYMRYSPRCAKTRAPISMELFQGLREFFRFAIPSAVMICLEWWSFELIILLSGLLLNPQLETSVLSICLNTTSILYAIPFGIGAAASTRISNELGAGNPHGACVSVLAAISFAIIETTVVSGTLFACRHVFGYVFSNEKEVVDYVTVMAPLVCISVILDNIQGVLAGVARGCGWQHIGVYVNIGAFYLCGIPMAILLSFFAKMRGKGLWIGVQVGSFAQCVLLSTITSCINWEQQTIKARKRLFGSEFSADDRLI*
Glyma.06G305200.1.Wm82.a2.v1	MDSTSVHKDIDEPLLVSEEPSSPQPPSFTQSFSSRHGSDGELERILSNTSVPFAKRIGPATWVELKFLFHLAAPAVIVYLINYVMSMSTQIFSGHLGNLELAAASLGNTGIQVFAYGLMLGMGSAVETLCGQAYGAKKFNMLGIYLQRSTVLLTLAGILLTIIYIFSEPILIFLGESPRIASAAALFVYGLIPQIFAYAINFPIQKFLQAQSIVAPSAYISAATLLVHLVLSYVVVYKVGLGLLGASLVLSVSWWIIVIAQFVYIVKSERCKHTWRGFSFQAFSGLAEFFKLSAASAVMLCLETWYFQILVLLAGLLPHPELALDSLSICTTFSGWVFMISVGFNAAASVRVSNELGARNPKSASFSVMVVTLISFIISVIVALVVLAIRDIISYAFTDGEEVAAAVSDLCPLLALSIVLNGIQPVLSGVAVGCGWQTFVAYVNVGCYYGIGIPLGSVLGFYFKLSAKGIWLGMLGGTVLQTIILVWVTFRTDWNNEVEEAAKRLNKWENKTEPLVN*
Glyma.06G318	MEEGSETGKWGWMKRRRAMREELKKVGTIAAPMVVASVLQYLLQVVSLVMVGHLNQLSLSTVAIATSLTNVSGFSVLSGMAGGLETLGGQAFGAGQYEKFGQYT

700.1.Wm82.a2.v1	YTAVISLSLICFPITILWTFMDKILTLLGQDPTISLEARKYAIWLIPALFGSAILKPLTRFFQTQSLISPMILTSAIALCFHGATCWTLVFKLELGHVGAAISFSLCVWFNVMLLLSFVRYSSACEKTRIPFSKNALVGVGDFFRFAVPAAVMVCLKWWACEILVLLAGLFPNPKLETSVLSICLTISTLHFTIPYGFGAAASTRVSNELGAGNPQAVRVAVSATMFLAVTEGLIVSATLFGCRHILGYAYSDDRMVVHYVAVMTPLLCLSIFTDSLQGVLSGVARGSGWQHLGAYVNLGAFYLVGIPVGIVLGFVAHLRAKGLWIGIVTGSIVQSILLSLVTALTNWKKQAMMARERIFDVKPPDENESNHMTSA*
Glyma.07G099900.1.Wm82.a2.v1	MGIWRNEISEEAKKQLWLSGPMVFVSVFQYSLQMISLMFVGHLDELLLAGVSLATSFVNVTGFSVLLGMSSALDTFCGQSYGAQQYHMVGIHMQRAIVIIMLATIPMSFIWAYLRPILVVLHQDKTIAAQAQLYATYLIPSLSANALLRCITKFLQTQNIVLPMMLASGFTTLAHALLCWLLVLKFGLGIKGAAIAFCISNWLNTVLLALYIRFSSSCKSTWTGFSRESLQNIPQFLSLAFPSALMVCLEQWTFQIMVLLSGALPNPKLQTSVLSICFNTTGLFWMIPFGVSVAASTRISNELGAGCPKAAYLAVKVTLLMSFVVGALGFILLMVTRNIWGHIFTNIPEVIRYVASMTPILASSVFVDSIQTALSGIVRGCGWQKLGAFVNLGSYYLVGLPFAIVLAFVLHIKGEGLLLGIVIALTMQVVGFLVITLRTNWEKEANKAAKRIRSNGVPTDANALPSDQN*
Glyma.07G100000.1.Wm82.a2.v1	MALWKEEAKKQLWLAGPMVFVSVFQYSLQVISLVFVGHLDELLLASTSLATSFVNATGFNVLMGMSSALDTFCGQAYGAKQFHMLGVHTQGAMLVLTLVTIPLSIIWVFLGPILVALHQDKEIAAHAQLYARYLIPSLSANGLLRCITKFLQTQNIVFPMVLATGLTSFLHFFLCWVLVQKVGLGIKGSAIAICISNWFNTIILALYIKLSPSCKTTWTGFSKESLHNIPKFLKLAFPSTLMVCLESWTFEIMVLLSGALPDAKLQTSMLSICLNTSGIFWMIPFGISAAGSTRISNELGAGSPKAAYLAVKVTMFLASAVGILEFASLMILWRVWGRVFTNVHEVVKYVTSMMPLVASSTFIDSIQTAFQGVARGCGWQKLGAYVNLGSYYFLGVPFSVVSAFVFHMKGQGLFLGILIALTVQVVCFLLVTLRANWEKEAKKAAKRVGGDGVQLGDLPCDQNANTA*
Glyma.07G100100.1.Wm82.a2.v1	MGIWDKEIADEVTKQLWVAGPMICVCVCQYSLQMMSLMFVGHLDELLLAGASLATSFVNVTGFNVLMGMSSALDTFCGQGRAMLVLILVTIPVSIIWVFLGPILVALHQDKEIAAQAQQYARLLIPSLSANGLLRLLHILLCWTFVLKLGLGFKGSVIAICISNWFNTILLTLYIRFSPSCKTTWTELDLFSLGGILNNPVDLFCLEAWTFEIMVLLSGALPNAKLQTSVLSICLNTTGIFWMVPFEVSAAGSTRISNELGAGRAKAAYLAVKVTMFLASAVGILEFAALLLVRRVWGRAFTNVHEVVTYVTSMIPIVASSPFIDSIQTAFQGVARGCGWQKLGAFFNLGSYYFLGVPFAIVTAFVLHMKGQGLLLGIVLALIVQVVCFLVVTLRTNWEKEANKAATRVGGSIVQVEHIKLS*
Glyma.07G124600.1.Wm82.a2.v1	MCESKSEQRHDDKEKQTEELGGVLTRRSTIMWDVIGEAKSLWELAFPTALTGLIFYARSMVSMLFLGHLGDTELAAGSLAIAFANITGYSVLSGLSLGMEPLCSQAFGAKRPKLLSLTLQRCVIFLLFSSIPISLLWLNMSKVFILLHQHTHITQMAQTYLVFLLPDLVTNSFLHPIRVYLRAQNITHPVTLASLAGTLLHVPFNLLLVQRGLPGVAAASAASSFSILSLLVLYVWISGVHLATWTAPSRECFGGWEPLLRLAAPSCVSVCLEWWWYEIMILLCGVLVDPTASVAAMGIFNPDDVVDLCLPLVSGICGFHARGEPAWREQGPRARMSAVVAVFFAAVMGFSAVVFATAMRRRWGRMFTGDEGILRLTAAALPILGLCELGNCPQTVGCGVVRGTARPNVAANVNLGAFYLVGMPVAVGLAFWLEVGFCGLWLGLLSAQVCCAGLMLYMIGTTDWEYQACRAQLLTALDQGSDGHKQPLLAAVDNNNS*
Glyma.	MASSLSTTSFLCTHSLKSPTLKPLTKPHCFARFRARVRVPPKASSQNNNANNDS

07G156 100.1. Wm82. a2.v1	VETPSLQPSSPKPPYNSSSSLLRHFSNGWFKFDELGMEIVLIALPAALALAADPIA<br>SLIDTAFVGHIGAVELAAVGVSASVFNLVSKAFNVPLLNVTTSFVAEEQALIRKE<br>EESILSDENGKDQSKKLLPSVSTSLALAATLGIAETVVLTLGSGILMNIMGIPADS<br>PMRGPAEQFLTLRAFGAPAIVLALAAQGTFRGFLDTKTPLYAVGVGNFLNAIL<br>DPILIFLFGLGIGGAAVATVISEYLIAFILLWKLSDKVLLIPSEFDGRKFFSYLKSGG<br>LVSARTLAVFITVTLSTSVAAQQGPIPMAGHQICMQVWLSVSLLNDALALAGQ<br>ALLARNYSLGNYEQARLVIYRVIQIGLGAGITLSIILFFGFGAFSSLFSTDSEVLDV<br>AWSGIWFVAGSQPVNALAFVIDGIYYGVSDFGYAAYSMVLVGLVSSTFLLVAAP<br>VGLPGVWTGLFIFMALRVLAGVWRLSSKSGPWDTIWYRDGAED*
Glyma. 07G246 100.1. Wm82. a2.v1	MLAEQKSQNTYPTVTEVLEEAKRMTDIGFPIAAMSLVGYLKNMTLVVCMGRL<br>GSLELAGGSLAIGFTNITGYSVLSGLAMGMEPLCTQAFGSRNFSLLSLTLQRTIL<br>MLLLFSLPISLLWLNLESLMLCLRQNPDITRVATLYCCFAIPDLIANCFLHPLRIY<br>LRSKGTTWPLLWCTLLSILLHLPTLTFLTFKLNLGVPGIAISSFVANFSNLFFLLLY<br>MFYTRVRKESLHVPLLMPRHMSHNVTTCSSTSTIAKEWGVLMKFSIQSCLAVCL<br>EWWWYELMTISAGYLHNPRVSLATAGIVIQTTSLMYTLPTALSASVSTRVGNEL<br>GAGQGERARLSTVVAIGLALVSSIFGLLWTTMGRERWGRVFTSDSEVLQLTMA<br>VLPIIGLCELANCPQTTSCGILRGSARPGVGAVINFCSFYLVGAPVAIVLAFYWK<br>LGMVGLCYGLLAAQIACVVSIFVVVYKTDWERESLKATCLVGKSSCGTFAYED<br>QTAVKCEGVVFLHNSEK*
Glyma. 08G033 200.1. Wm82. a2.v1	MGSKTEEVVDYTLMEGAVEKEGQHCWSIRREVKAVGELAFPIALTALIFYARS<br>MVSMLFLGHLGELELAAGSLGMAFANITGYSVLSGLALGMEPLCSQAFGAKRV<br>NVLSLTLHRCVMFLLLCSIPISLLWLNMSNILVDLLHQDPNITLMAHTYLLFSLP<br>DLLTHSFLHPIRIYLRAQGVTHPVTLASLAGTLLHLPFNYLLVTRLRLGLAGVAA<br>ASAASNLSILLFLGAAVFFSGLHCSAPSRECLSGWKPLLRLAAPSCVSVCLEWW<br>WYEIMIILCGLLVDPTATVASMGILIQITSLIYVFPSSLGFAVSTRVGNALGANRPS<br>RAKLSAVVSVFLAAIMGFSAMFFAVGMRRRWGTMFTADEDILRITSMALPILGI<br>CELGNCPQTVGCGVVRGTARPNTAANVNLGAFYLVGMPVAVGLGFWFDVGF<br>CGLWLGLLSAQVCCAGLMLYVIGTTDWEFEAHRAQLLTLVEDGVMDGQKQP<br>LTGVVTETPSS*
Glyma. 08G050 500.1. Wm82. a2.v1	MDREDQKASLLSQLIHITVEDRLKVDNIQRNKDKQQAIERAELYEEVRKQLWL<br>AGPLISVSMLNYSQQIISVMFVGHLGQLPLSGASMATSFASVTGFSLLVGMASAL<br>DTLCGQSYGAKQHHMLGIHMQRAMLVLMIVSINLAFIWANTRSILVALGQDP<br>EISAEAGQYAQLMIPSLFAYGILQCLNRFLQTQNIVFPMVFSSGVTTLLHILICWT<br>MVFKSGLGNKGAAIANAISYWINVLILILYVKFSPSCSKTWTGFSKEALHGIPSFL<br>KLAIPSALMVCLEMWSFEMMVLLSGLLPNPKLETSVLSICLNTSTSVWMIPFGLS<br>GAVSTRVSNELGAGHPRAARLAVYFVFIMAIIEGTFVGAVMIIIRNIWGYAYSNE<br>AEVVQYVAIMLPILATSIFLDALQCVLSGTARGCGWQKKGAFINLGSYYLVGIPS<br>AILFAFVLHIGGKGLWLGIICALVVQVSCLLIITIRTDWEQEAKKVKDRVYDSMR<br>AEVIVS*
Glyma. 08G050 700.1. Wm82. a2.v1	MRIGREEVVEEVKRLLWLAVPLFSVGILQYILQAISVMFIGHLGTLPLSGASMASS<br>FASATGFNLLLGLATALDTFCGQSNGAGQYHMLGIHMQRSMLVVLMMSVFLS<br>IIWYNTEPILKAMHQDKAISKEAGSYTQYMIPSLFAYGLLQCILKFLQTQKIVFP<br>MVLTSGIAAVLHVLLCWLLVFKSGLGIKGAALANSISYWINVILISLYVRFSSAC<br>KHSWTGFSKMALHNLLDFLKLAAPSAVMHCLKVWTFELMVLMSGLLPNPKLE

	TSVLSICLNTFGLAWMIPFGFSAAVSVRVSNELGSGNPQAASLAVRVVLSMALIE<br>GVILVSAMILLRNVWGHVYSNDKEVIRYVSFMMPVLALSSFLDGIQGTLSGILA<br>GCGWQKIGAYVNLGSFYLVGVPCAVVLAFIVHMKAKGLWMGIIFAFIVQVSLY<br>IIITFRTNWEEQARKAQRRVELITIPPTTRDSVLPHQKQELVP*
Glyma.08G244400.1.Wm82.a2.v1	MAYGGGGFAISYPLAKALHIISWIVLFLTAICLLPLFIFTSPILTLLGQDESITEVAR<br>TICICSIPILFSYIVSNNCQTFLQSQSNNVIISFLAALSIIIHVSLSWLFTMQFKYGIPR<br>AMISTILAYWIPNIGQLIFITCDWCPETWKGFSFLAFKDLWPASKLSISFGAMLS*
Glyma.08G282600.1.Wm82.a2.v1	MEDHDDQTHPLLTRRHHTPDSSAVVFTATSDDIAPIGGAGDFAREFFAESKKL<br>WYLAGPAIFTSVCQYSLGGVTQVFSVHVNTLALAAVSVENSVIAGFSLGITFGM<br>GSALETLCGQAYGAGQVHMLGVYMQRSWVILNATAILLSLLYIFAGHMLRAIG<br>QTEAISAAAGEFALWMIPQLFAYAVNYPAQKFLQAQSRIMVMAWIAAAALVL<br>HTLFSWLLILEFGWGLVGAAVVLNASWWFIDIAQLVYIVSGACGEAWSGFTFK<br>AFHNLWGFVRLSLASAVMLCLEVWYFMALVLFAGYLKNAEVSVDALSICMNI<br>LGWTIMVSFGMNAAVSVRVSHELGASHPRTAKFS
Glyma.09G042700.1.Wm82.a2.v1	MVAEGKSQKPYPTAAEVVEELKRMGDIGVPIAAMSLAGYIKNMVLVVCMGRL<br>GSLELAGGALAIGFTNITGFSVLSGLAMGMEPLCTQAFGSRNFSLVSLTLQRTIL<br>MLLAASLPISLLWLKLEPLMLWLHQNPEITKVASVYCFFSIPDLIANSFLHPIRIYL<br>RSKGTTWPLLWCTLLSILIHIPIVAFFTFKLHLGVPGIAMSAFVANFNTLFFLLSY<br>MLYMRVSKGSLSMPLLLSSTCQLPSPSPQQQQQQHHHQDQTSLKTTTLGKEWG<br>MLIRFSIQSCLGVCLEWWWYEFMTILAGYLYNPRVALATAGIVIQTTSLMYTLPT<br>ALSASVSTRVGNELGAGQPERAKLSTIVAIGLSLASSILGLLWTTIGRERWGRVFT<br>SDSEVLELTMSVLPIIGVCELANCPQTTSCGILRGSARPGVGAGINFYSFYLVGAP<br>VAIVIAFVWKLGLVGLCYGLLAAQIACVVSILVVVYNTDWERESMKAKSLVGIF<br>KSSCDHDHHYGDQTVKCEEGLLSFSMRKNSDQKK*
Glyma.09G102800.1.Wm82.a2.v1	MVINIKQAYFFILMDIMDENGSSSNAPKNKRNLPVSVFFKDARHVFKMDSIAKE<br>ILGIAFPSALAVAADPIASLIDTAFIGHLGPVELAAAGVSIALFNQASRITIFPLVSI<br>TTSFVAEENTIEKINTEKKLSDKAKSKEQVMLDDHSLQDIEKVASKENNETENV<br>EMNDCNTSICKSTSDTSSSSSNKSVPKDGRKKRHVASASTALLFGTILGLLQATT<br>LIFAAKPLLAAMGLKPDSPMLNPAIKYLRLRSLGAPAVLLSLAMQGIFRGFKDT<br>TTPLYVILSGYALNVILDPVLIFYCKLGIKGAAISHVLSQYLMALALMVILTRKVD<br>LVPPSIKDLQIFRFLKNGGLLLARVIAVTFCQTLAASLAARFGPIPMAAFQTCLQ<br>VWLTSSLLADGLAVAVQAILACSFAEKDYEKVLVAATRTLQMSFVLGVGLSFA<br>VGFGLYFGAGIFSKSVLVVHLIRIGLPFVAATQPINSLAFVFDGVNYGASDFAYS<br>AYSLVTVSLASVASLFLLSKSKGFVGIWIALTIYMSLRMFAGVWRMGTGTGPWR<br>FLRGRSMS*
Glyma.09G134000.1.Wm82.a2.v1	MTILSTSLTSIYAGHIGDIELSSIALYQGVMSAIYFYLLFGMSSALATLCGQAFGA<br>GQIQSTCIYVQRSWIILTATCTILLPIFVYATPILKLLGQDEGIAELAGRYSIQVHR<br>QKNPTFAYFGFGLCGLPDLRSCCCGTSSTSSCWCCWSCYVGDGGSVHVLGLVT<br>SCISLTSVLHCQLAPSSKMTSLEMINCFESVIPYMFSFAIAFPVQRFLLAQSKVKVI<br>MCIAFVDLLIQNGLPYIFINVFGWGVTSLAITANIVGWLYAVALVVYTIDWCRE<br>KWISLQSSVMNCLEQWYITCIMLHAGLLDNPVIGVGSYSICFNIQGWDDMLLL<br>GINTVICIGSIPSKCSCLQYAWQVAAIYSFCMKMFLSLLLGILFMTVIFFSKDEFAK

	IFTDSEDMILADSDLAHLLGVTIVLNSASQVMSGVAIGSRWQVMVGYINLACYY<br>IVGLPIGIFLGFKLHLGVKGLWGGTMCSSILQILVLFTIILKTKWSKEMEQTAHR<br>MRLWSNNITTSPSKKMLYEREQCGVI*
Glyma.<br>09G134<br>100.1.<br>Wm82.<br>a2.v1	METPLVVQNFTSEADYFPVKSLKDVKFVLWAETVKIWRIALPVALTHLFQVLT<br>NSSTSIYAGHLGDIELSSISVSQGVMSSIYFQLLFGMSSALATLCGQAFGAGQIQS<br>TCIYVQRSWIILTATCIILLPIYIYATPILKLLGQDEGIANLAGRYSIQVIPHMFSFAI<br>VFPTLRFLQAQSKVKVIMCIAFVVLLIQNGLLYIFINIFGWGITGLAMVSNIIGWL<br>YAGALVVYTISWCKEEWSGFSWMAFRDLLAFAKLSLQSSVMGCLEQWYMTCI<br>MLLAGLLDNPVIAVGSYSICFSVQGWHFMLLLGISTAISVRISNALGMSQPRAA<br>KYTFCVTMFQSLLLGVLFMNVIFLTKEDFAIIFTNSEDMIQAVADLAYLLGVTM<br>VLNSASQVMSGVAIGSGWQVMVAFINLACYYIVGLPIGYFLGFKQHLGVKGLW<br>GGTMCGSVLQILILLLIIRKTNWTKEVEQTAHRMRIWNVNNFRSDLAENVT*
Glyma.<br>09G134<br>200.1.<br>Wm82.<br>a2.v1	METPLVTEKFTSESDYLPVKSLKDVKFVLWTETVKIWRIAFPMALSALFQFLTISS<br>TSIYAGHIGDIELSSISVYQGVISALYFYLLFGMSSALVTLCGQAYGAGQIQSTCIY<br>VQRSWIILTATCIILLPIYVYATPILNFIGQDQEIADLAGRYSIQVIPYMFSCAIAFP<br>FQTFLQSQIKVKVITCIALAVLVIQNVLLYIFINVFGWGTTGLAMVTNIIGWVYA<br>AALVVYTIGWCKEEWTGFSWMAFRDLWSFAKLSLASSVMSCLDQWYSTCIILL<br>AGLLDNPVIDVGSYSICFNVQGWHSMLLLGISAAISIRVSYILGKSHPRAAIYSFC<br>VTMFQSLLLGIVFMTVIFLSKDEFAKIFTNSKDMIRAVADLAYLLGVSMVINSAS<br>HVMSGVAVGSGWQVMVGYINLACYYIVGLPIGIFLGFNQHLGVKGLWGGTM<br>CGRILQMLVLLVIIWKTNWSKEVEQTAHRMRIWSINNLHSDAMGNVT*
Glyma.<br>09G146<br>800.1.<br>Wm82.<br>a2.v1	MCHPTLDVTSNCKCCSTSPPDCPISIKDPLISKNQNNNTSSLPPPPPPQQHNNTT<br>TTTTKTKTPPPPPPPSTTTTKTKATTTSKPHNFPESIKEVFSISKIAIPMILTGLLLYC<br>RSMISMLFLGHLGELALAGGSLAVGFANITGYSILSGLAVGMEPICGQAFGAKR<br>FTLLGLCLQRTILLLLFTSLPITLLWLYMKQILLLCGQDEAIATQAQQYLVYSIPD<br>LIAQSFLHPLRIYLRTQSITLPLTLCASFSILLHIPINYFLVAHLKLGIKGVALGGV<br>WTNFNLVASLILYIVFSSTHKKTWGGFSFECFTQWKSLLNLAIPSCVSVCLEWW<br>WYEIMILLCGLLVNPKATVASMGILIQTTSLLYIFPSSLSFSVSTRVGNKLGAQKP<br>SKARLSAIVGLSCSFMSGVLALFFALMVRNTWASMFTKDKEIITLTSMVLPIIGLC<br>ELGNCPQTTGCGVLRGTARPKVGANINLGCFYLVGMPVSIWLAFFTGYDFQGL<br>WLGLLAAQGSCAVTMLVVLCRTDWEFEAQRAKKLTGMGVDHNHHEVDDPE<br>KPLKHESKEDSLLLADSDENEQ*
Glyma.<br>09G178<br>700.1.<br>Wm82.<br>a2.v1	MKRPLPPSVSSDNVEDRKMKKKVKALDTTTMWSAWRASMFDDNLLFEVLKHI<br>DTSDVGMHKQAVTQGSHVIESEGMMDFLVGLLVFWRRVSLEASTFEIIVLLTGA<br>LPNSKLQTSVLSICLNTTGVFWMVPFGVSVAGSIRISNELGDGSAKAAYLAVKV<br>TMFLGSAVGILEFAVLMLVRKVWGRAFTNIHEVATYVTAIIPIVASSAFIDSIQTA<br>FQGVARGCDRQKLGALINLGSYYLLGVPFAIVTACVLHTKGQVKSSHFVIIKKD<br>LTINNILKKGIVLALIVQVVCFLVVTLRTKWEKEVSCPIFNSVAMLSFINYGY*
Glyma.<br>09G178<br>800.1.<br>Wm82.<br>a2.v1	KMALWKEEAKKQLWLAGPMVFVSVFQYSLQVISLMFVGHLDELLLASASLATS<br>FVNATGFNVLMGMSSALDTFCGQAYGAKQFHMLGVHTQGAMLVLTLVTIPLS<br>IIWVFLGPILVALRQDKEIAAHAQLYARYLIPSLSANALLRCITKFLQTQNIVFTM<br>VLASGLTSLLHFFLCWALVQKIELGIKGSAIAICISNWFNTIILALYIKLSPSCKTT<br>WTGFSKESLHNIPRFLRLAFPSTLMVCLESWTFEIMVLLSGALPNAKLQTSVLSIC<br>LNTSGIFWMIPFGISAAGSTRISNELGAGSPKAAYLAVKVTMFLASAVGILEFASL

	MLLWRVWGHVFTNVHEVVKYVTSMMPLVASSTFIDSIQTAFQGVARGCGWQ<br>KLGAYVNLGSYYFLGVPFSVVSAFVFHMKGQGLFLGILIALIVQVVCFLLVTLRA<br>NWEKEIEI*
Glyma.<br>09G178<br>900.1.<br>Wm82.<br>a2.v1	MKRVSRQEVVEEMKKQAWLAGPLFTVGVLQYSLQVISVMFVGHLGELPLSGAS<br>LATSFASVTGFNLLMGMASALDTLCGQSFGAGQHHMLGIQMQRATFVLSFVS<br>VFLAIMLVFTKHILVAMHQQVAIAEEAGVYAIYMIPSLFAYGIFQCLLKFLQTQ<br>NIVFPMVLSSAVVALLHIPLCWVLVIKSGIGSKGAAIANSVSYWLNVLLIGFYVK<br>FSSSCAKTWTGFSVKALQNIPEFLKISIPSACMLCLKAWTFELMVLLSGLLPNPQ<br>LETSVLSICLNTFVIAWMIPFGLSCAVSTRVSNELGAGHPQAASLAVRVALFLVL<br>ADGIMMVLVMILLRKIWGNLYSSDTHVIKYVAAVMPILATCSFLDGIQSVLSGI<br>ARGSGWQKIGAIVNLGSFYFVGVPSSVVLAFVLHMKGKGLWLGIVSAFIVQVIL<br>FGVITIRTSWDKEANKAAMRVKDTKIPQELPQRDPFTITEMN*
Glyma.<br>09G179<br>000.1.<br>Wm82.<br>a2.v1	MERGDQNQSASLQSPLIKHSHSSSGRGIERREVIEEVKKQLWLAGPLISVTLLNF<br>CLSIISVMFVGHLGELSLSGASMATSFASVTGFSLLVGMASSLDTFCGQSYGAKQ<br>YHMLGIHLQRAMFTLMIVSIPLAIIWANTRSILTFLGQDPEIAAEAGSYARFMLP<br>SLFAYGLLQCLNRFLQTQNIVFPMMCSSAITTLLHVLICWILVFKSGLGNRGAA<br>VANSISYWLNVTILSLYVMFSPSCAKSWTGFSKEALHNIPSFVRLAIPSAVMVCL<br>EMWSFELMVLLSGLLPNPKLETSVLSICLNTTAAAWMIPFGLSGAGSIRVSNELG<br>AGRPWNARLAVRVVLVLAIIEGIIVGTVMILVRNIWGYAYSNEVEVVEYVATMF<br>PILAASNFLDGLQCVLSGTARGCGWQKIGAFVNLGSYYIVGIPSSIVFAFVLHIG<br>GKGLWLGIICALIVQMCSLMIITIRTDWDQEAKKATDRVYNSVTLESLVS*
Glyma.<br>09G257<br>400.1.<br>Wm82.<br>a2.v1	METPLMFNSDLNSTGTTELQYAPEGLVDTNGGDYTEMSGLADFKNVFSVESVK<br>LWTIAAPIAFSILCNYAVNSFTTIFVGHLGDLELSSVSLSLSVVSNFSFGFLLGMAS<br>ALETLCGQAFGAGQVEMLGVYMQRSWLILLGACICLTPIYIYAEPILLLLGQEPE<br>IAELAGVFTIQSIPQMFSLAINFPTQKFLQAQTKVGFLAWVGFGAFIFHIILLWILL<br>KVLALGTTGAAVAYSTTAWVIALAQTAYVIGWCKDGWRGFSWLAFKDLWAF<br>VKLSVASAVMLCLEVWYFMILIVLTGHLDNAVIAVGSLSICMTINGFEGMLFIGI<br>NAAISVRVSNELGSGRPRAAKYSVIVTIIESLVIGLICAAIILITKDHFAIIFTESKEM<br>IKAVSKLAGLLGLTMILNSVQPVISGVAVGGGWQALVAYINLFCYYIMGLPLGF<br>LLGYKLGYRVEGIWVGMICGTILQTLILLYIVYKTNWNKEVELASSRMRKWTGQ<br>EIEINLANSQQPVTH*
Glyma.<br>09G276<br>100.1.<br>Wm82.<br>a2.v1	MCTGAESRGIVDGNNPVTNSKVGGVYHEINGMRSTEGGDGEGKRNSGCSGRFI<br>QGLHQHLHQLLKALFPTLSLSEVKEELKSLANIACPMMMTNVLLYSRSAISMLY<br>LGRQGKVELAGGSLAIGFANITANSFLKGLTMGMDPICCQAYGAKRWSVLSQT<br>FCKTLCLLLLVAIPISLLWLNMAPLLHWLGQDPEVTKVAQVYMVFSIPELLAQV<br>HLNPLRSFLRTQGLTTPLTIAASFAAILHLPINYFLATYLELGVKGIALATGLNSI<br>NMILGLVLYLVVSEKPLKPWEGVTILSSFHDWRPLLTLALPSCISVCLEWWCYEI<br>MLFLCGLLSNPQTTIATMGVLIQTTGFLYVFPFSLSAALTTQIGHSLGAGQPSRA<br>QNTAKIGLFIAFALGVSAFVFLLFVRNVWGKLFTNETQIVDMVTAILPILGLCEI<br>GNWPQTAACGILSGTARPYVGARINLCAFYLIGLPVAIFAAFMHRYQLRGLWF<br>GMLAAQISCFCMMVYTLVQTDWGHQSRRAEQLAQTTDEENVNNDEKSGLLD<br>SDL*
Glyma.<br>10G099	MLPLLMLFSSIRNAFSSDELGLEILNISLPTTLALAADPIASLIDTAFIGHIGPVELA<br>AVGVSIAIFNQISKITIIPLVSVTTSLVAEEDAVDEQNQQSEREMLMKVSNEDVK

900.1.<br>Wm82.<br>a2.v1	LDVHDHAEKAGNSSSANVGRVAKLDHDKSYIPSASSGIVIGGLLGVLQALFLIF<br>TAKPMLSYMGVDSNSPMFKPAQQYLTLRSFGAPAVIISMAIQGVFRGIKDTKTP<br>LYATVMGDVTNIILDPLLMFVLRLGVNGAAISHIISQYLIAIMLLWSLMKQVVLL<br>PPSIQDFRFGKILKNGFLLLIKVASATFCVTLSTSLAARKGSTTMAAFQICLQIWM<br>ATSLLADGLAVAAQAIIASAFARDDYKKVIASASRVLQLGLILGLVLSVLLLSLL<br>PFASRLFTNDINVLQLISIGIPYVAATQPINALAFVFDGVNYGASDFTYSAYSMIM<br>VALVSILSLYMLSSSLGFTGIWIALLIYMTLRIFAGFWRIGTGSGPWSFLKENSVEL<br>Q*
Glyma.<br>10G231<br>800.1.<br>Wm82.<br>a2.v1	MITVFLSKDNLAIIFTSSKDMQLTVADQAYLLGLTMIINSAAQLMSGVAIGSGW<br>KLTVAYINLTCYYMVGLPLGFYLVSH*
Glyma.<br>10G232<br>200.1.<br>Wm82.<br>a2.v1	MDAPLLLAKGEGAALVTENGDYVAVRELKEVKKVFWIETKRVWQIAMPIVFNI<br>WCQFGVNSVTSMFVGHLGDIQLSAISLINSVIGTFAFGFMLGMGSATETLCGQA<br>FGAGQVNMLGVYMQRSWVILSVTSILLLPIYIFAGPILKFLGQQEDIADLAGSFSI<br>LVIPQFLSLPFNFPTQKFLQAQSKVNIIAWIGLVALILHIGMLWLLIYVLDFGLAG<br>AALAFDITSWGITVAQLVYVVIWCKDGWTGLSWLAFKDIWAFVRLSLASAVM<br>LCLEVWYMMSVIVLAGNLDNALVAVDSLSICMNINGWEAMLFIGVNAAVSVR<br>VSNELGLGHPRAAKYSVYVTVFQSLFLGIFFMAIILATRDYYAIIFTNSEVLHKAV<br>AKLGYLLAVTMVLNSVQPVVSGVAIGGGWQALVAYINIGCYYLFGLPLGFLLG<br>YEANLGVEGLWGGMICGIVIQTLLLLLILYKTNWKKEVEQTTERMRIWGGQDI<br>GVDKIVAST*
Glyma.<br>10G239<br>000.1.<br>Wm82.<br>a2.v1	MCHISSQLPCKCNSMKSQEAEESDMKITNPLIQKDITVTPPHHHVLKELISISKIA<br>LPMILTGLLLYCRSMISMLFLGRLGELALAGGSLAVGFANITGYSILSGLAVGME<br>PFCGQAYGAKKFTLLGLCLQRTILLLLFTSIPISLLWLYMKHILLLCGQDEAIATQ<br>AQSYLLYSIPDLLAQSFLHPLRIYLRSQSITLPLTLCATFSILLHIPINYLLVSHLNW<br>GIKGVALSGVWTNFNLIASLILYIVFSGTHKKTWGGFSFECFTQWKSLLDLAIPS<br>CISVCLEWWWYEIMILLCGLLVNPRATVASMGILIQTTSLLYILPSSISFSVSTRVG<br>NKLGAQKPSKAKLSAIVGLSCSFMLGFLAFVFTILVRNIWASMFTQDKEIITLTSL<br>VLPIIGLCELGNCPQTTGCGVLRGTARPKVGANINLGCFYLVGMPVAVWLGFF<br>AGLDFQGLWLGLLAAQGSCAVTMLVVMSQTDWDVEALRAKKLTSVVVAVD<br>DSKEVGAEKPPKAEIKEDSLLSLADSEEDKQSWV*
Glyma.<br>10G267<br>300.1.<br>Wm82.<br>a2.v1	MEENLIITKQSEKQRVTWDGLGEEMKRIIRVAGPMVFVYASQNLLQVVSIMMIG<br>HLNDELFLSGAALAISLATVTGFSLLTGMASGLETICGQAYGARQYQKTGVQTY<br>TAIFSLTCVCLPLTIIWISLENILVFIGQDPLIAHEAGNFIIWLLPALFAYAILQPLV<br>RYFQMQSLLLPMLATSCVTLCLHIPLCWALVFKTELSNVGGALAMSISIWLNVI<br>FLVLYMRYSPACEKTRAPVSMELFQGIWEFFRFAIPSAVMICLEWWSFELLILLSG<br>LLPNPQLETSVLSICLNTISTLYAIAFGIAAAASTRISNELGAGNPHSARVAVLAS<br>MSFAIMEATIISGILFVCRHVFGYTFSNKKEVVDYVTVMAPLVCISVILDNIQGVL<br>AGIARGCGWQHIGVYVNLGAFYLCGIPVAASLAFLAKMSGKGLWIGLQVGAF<br>VQCALLSTVTSCTNWEQQAMKARKRLFDSEISAENILV*
Glyma.<br>10G267	MEENLLAKQREKQKVTWDGLGEEMKRIICIAVPMVIVTATQYLLQVVSIMMVG<br>HLNNNLYLSGAALAISLATVTGFSVLAGMASGLETICGQAYGAQQYEKVGVQT

400.1. Wm82. a2.v1	YTAIFSLTVVCLPLTFIWISMEKILVFIGQDPLIAQEAGKFLIWLVPALFAHAIMQ<br>PFVRYFQMQSLLLPMLISSCVTLCIHIPLCWALVFQTGMNNIGGALAMSISIWLN<br>VTFLGLYMRYSPACAKTRAPISMELFQGIWEFFRFAIPSAVMICLEWWSFELLILL<br>SGLLPNPQLETSVLSICLNTISTLFSIPFGIAAAASTRISNELGAGNPHAAHVAVL<br>AAMSFAIMETAIVSGTLFVCRHDFGYIFSNEKEVVDYVTVMAPLICISVILDSIQG<br>VLAGVARGCGWQHIGVYVNLGAFYLCGIPVAATLAFLAKMRGKGLWIGVQV<br>GAFVQCILFSTITSCINWEQQAIKARKRLFDSEISADNRLV*
Glyma. 10G267 600.1. Wm82. a2.v1	MSFTITEATIVSGTLFACRHVFGYIFSNEKKVVDYVTVMAPLICISVILDSIQGVLA<br>GVARGCGWQHIGVYVNLVAYYLCGIPVAASLAFLEKMRGKGLWIGVQVGAFV<br>QCVLLSIITSCINWEQQAIKARKRLFDSEFPADNRLV*
Glyma. 10G267 700.1. Wm82. a2.v1	MEESLVKKHEQERVTWGVYSEEMRRVCHIAGPMVAVVSSQYLLQVVSTMIVG<br>HLGELYLSSAALAISLSGVTGFSLLMGMASGLETICGQAYGGQQYQRIGIQTYTA<br>IFSLILVSIPVSLLWINMETILVFIGQDPLISHEAGKFTIWLVPALFAYAILQPLVRY<br>FQIQSLLLPMFASSCVTLIIHVPLCWALVFKTSLSNVGGALAVSISIWSNVIFLVLY<br>MRYSSACAKTRAPISMELFKGMWEFFRFAIPSAVMVCLEWWSYELLVLLSGLLP<br>NPQLETSVLSVCLNTIATLYTIPFGIGAAASTRVSNELGAGNSHAARVAVLAAM<br>SLAVIETSIVSATLFACRNVFGYIFSNEKEVVDYVTAMAPLVCISVILDSIQGVLTG<br>IARGCGWQHLGVYVNLGAFYLCGIPMAALLAFLVRLGGKGLWIGIQSGAFVQ<br>CILLSIITGCINWEKQAIKARKRLFDEKISADNILV*
Glyma. 10G267 800.1. Wm82. a2.v1	MKRVGYLVGPMITVTLSQYFLQIISMVMVGHLGKLALSSTAIAISLCAVSGFSLIF<br>AMSCALETQCGQAYGAHQYRKFGVQMYTAIVSLTLACLPLSPLWVYLGKILIFL<br>GQDPLISQEAGKFALCMTPALFDYATLQALVRYFLMQSLTPNEKTCISHLFNSL<br>VISSSITLCFHVAFCWLLVFKFGFGNLGAAFFIGTSYWLNVILLVLYMKFSIECKK<br>TWVPISTELFHGIGEFFRCAIPSAGMICLEWWSFELLFLLSGLLPNPELETSVLSIC<br>LSVTTTIYTIPEAIGSAASTRVSNALGGGSPQLAQVSVSAAMTLAASAAILVSSIIF<br>ACRQVVGYAFSNELDVVDYFTEMVPLLSISVILDTLHDTLSGIARGCGWQHRG<br>AYVNLDAYYVVGIPIAAILGFCLQLRGKGLWIGILTGAFCQTVMVSLITSCTNW<br>EKQVCLHFQVHALHLAQS*
Glyma. 11G026 300.1. Wm82. a2.v1	MCQLSSSPSTLCDSNEGHPNMPTTKIHEEPAHMLPLLIPKPPTTLFEQGQNNHQ<br>TKTHFSLALNEVKCIANIALPMVLTGLLLYSRSVISMLFLGRVGELALAGGSLAI<br>GFANITGYSILSGLAMGMEPICGQAFGAKRFKLLGLAMQRTIVLLLLTSVLISFL<br>WLNMKKLLILCGQQEDIATEAQSFILFSIPDLVAQSLLHPLRIYLRSQSITLPLTYT<br>ASLSILLHVPINYFLVSVLKLGIKGIALGAVWTNFNLVVSLILYIWVSGVYKKTW<br>PGVSLKGILSGWKSLLNLAIPSCISVCLEWWWYEIMILLCGLLINPQATVASMGV<br>LIQTTALIYIFPSSLSFAVSTRVGNELGAENPKKAKVAALVGLCISYGLGFSALFF<br>AVSVRQAWASMFTRDAEIIALTSMVLPIIGLCELGNCPQTTVCGVLRGTARPKL<br>GANINLGCFYLVGMPVAVRLSFFAGFDFKGLWLGLLAAQASCMFTMLIVLART<br>NWEGQVQRAKELTSSSEEQDQNCFFSSCATKEYCSDSLLV*
Glyma. 11G028 900.1. Wm82.	MGFRSGFDELRVQRGIALPMVAMNLAWFAKTAITTAFLGRLGELSLAGGALGF<br>TFANVTGFSVLNGLCGAMEPIYVDRLMTLLMTISLLLLVSLPITFLWLNVDKILIL<br>FGQQQDISTVARTYVSCLIPDLFVASLFCPLKAYLSSQTITLPTMFSSAVALAFHIP<br>INIVLSRTMGLRGISMAVWITDLIVVVLLAIYVLILERKKESMWKEGGWWDQSIE

a2.v1	DWIRLLKLCGSCCLNTCLEWWCYEILVLLTGHLTNAKQAVGVLAIVLNFDYLL<br>FSVMLSLATCVCTRVSNELGANQAGLAYRSACVSLALGFISGCIGSLVMVAARG<br>IWGPLFSHDVGIIKGVKKTMLLMGLVEVFNFPLAVCGGIVRGTARPWLGMYA<br>NLGGFYFLALPLGVVSAFKLRLGLVGLFIGLLTGIVTCLTLLLVFIARLNWVEEA<br>AQAQTLTGQEQVKELSKYDAEELIDAHKKDVV*
Glyma.<br>11G112<br>100.1.<br>Wm82.<br>a2.v1	MAFKLKSLPLLCSLHSPSRQNPNSTLSNHHFSRRFFAPSIPPTLCLSGAASASTFH<br>RHRFFVTARSQDEDQITEALEQEEEKDNEEISRQGEKKELAKQGIWDQIKEIVMF<br>TGPATGLWICGPLMSLIDTAVIGQRSSIELAALGPATVVCDYMCYVFMFLSIATS<br>NMVATALAKQDKEEVQHHISVLLFVGLSCGIAMLLFTRLFGAAIITAFTGPKNV<br>HVVPAASNYVKIRGLASPALLVGWVAQSASLGMKDSLGPLKALAAATVINVA<br>GCVLLCTYLGYGIVGAAWATMVSQVVASYMMIQNLNMKGYNALAFSIPSGKE<br>LLTIFGLAAPVFITLMSKVAFYALLIYFATSMGTHTMAAHQVMVQTYLMCTVW<br>GEPLSQTSQSFMPELIYGVNRSLSKARMLLRSLVIIGAILGLLLGIIGTSVPWLFPNI<br>FTPDRMVIQEMHKVLIPYFIALAVTPPTVSLEGTLLAGRDLKFISLSMSGCFCVGS<br>LVLWALSSRYGLLGCWFSLALFQWARFSMALQRLLSPKGILYSEDTEQYKLLKL<br>RTA*
Glyma.<br>11G112<br>200.1.<br>Wm82.<br>a2.v1	MALKLSSLSLHSSLRHQHPNSSHSNHRIPLRFFAPSLPPTSCLSGAASVSTFHRTL<br>FAVTVRAFQSQDESKSSDVFEEEEKDEEISRQGEKKELAKQSIWSQIKEIVMFTGP<br>ATGLWICGPLMSLIDTAVIGQRSSIELAALGPATVVCDYMSYVFMFLSIATSNMV<br>ATALAKQDKEEVQHHISVLLFIGLSCGVGMLLFSRLFGASLITAFTGPKNAHVV<br>PAASNYVKIRGLAWPALLVGWVAQSASLGMKDSLGPLKALAAATVINFAGCI<br>LLCTYLGYGIVGAAWATMVAQVVAAYMMIQNLNMKGYNALAFSIPTGKEIL<br>MILGLAAPVFLTLMSKVAFYALLIYFATSMGTHTMAAHQVMVQTYGMCTVW<br>GEPLSQTAQSFMPELIYGVNRSLSKARLLLKSLVTIGAMLGLLLGIVGTSVPWLF<br>PYVFTPDRMVIQEMHKVLIPYFIALAITPPTHSLEGTLLAGRDLKFISLSMTGCFC<br>VGTLVLWALSSRFGLLGCWFSLALFQWARFSIALRRLLSPKGILYSEDTDQYKLR<br>KLRTA*
Glyma.<br>11G133<br>900.1.<br>Wm82.<br>a2.v1	MSLILGLALAFLLGTGLHFGAKLFTKDVNVLHLIRIEIPFVAATQSLNSLAFVFD<br>GINFGASDFAYSAISLDAVAIVSIICLLILSSASGFIGTWIAMTIYMDLRAIVGFLRI<br>GTRSGPWELLNY*
Glyma.<br>12G099<br>300.1.<br>Wm82.<br>a2.v1	MDSTSVHKDIDEPLLVSNEPSPEPPSCTQSFSSKHGSDGELERILSNTSVPFAKRL<br>GPATWVELKLLFHLAAPAVIVYLINYVMSMSTQIFSGHLGNLELAAASLGNTGI<br>QVFAYGLMLGMGSAVETLCGQAYGAKKFDMLGIYLQRSTVLLTLAGIILTIIYIF<br>SEPILIFLGESPRIASAAALFVYGLIPQIFAYAVNFPIQKFLQAQSIVAPSAYISTATL<br>LVHLVLSYFVVYEVGLGLLGASLVLSVSWWIIVIAQFVYIVKSEKCKHTWRGFSF<br>QAFSGLPEFFKLSAASAVMLCLETWYFQILVLLAGLLPHPELALDSLSICTTVSG<br>WVFMISVGFNAAASVRVSNELGARNPKSASFSVVVVTLISFIISVIVALVVLAIRD<br>VISYASQTVKRWLLLSQIFVLYLLFLLFSTAFNLSYLGWLLDVDGKLLLRM*
Glyma.<br>12G122<br>000.1.<br>Wm82.	MEQVDLVPPSIKHLQLDRFLKNDKGMSASLVARQGPTSMAAFQAILAGAFAN<br>KDFDKATATASRVLQRHTLCKCDALFVILHMGLVLGLALAFILGTGLHFGAKIF<br>TQDANVHHLIQIGIPFVAVTQPLNSLAFVFYGINFGASDFAYSAFSMVVVAILSII<br>CLLILSSVGGFIGIWVALTIYMGLRAFAGFWRIGTGSGPWEFLRSS*

a2.v1	
Glyma.12G194000.1.Wm82.a2.v1	MGSAEKEPLLTAEGTRSNSKHESSSHGQLERILSDDTLPFLNRVGPATWIELKLL<br>FFLAAPAVIVYLINYLMSMSTQIFSGHLGNLELAAASLGNTGIQMFAYGLMLG<br>MGSAVETLCGQAFGAQKYGMLGVYMQRSTILLSLAGVVLTVIYVFSEPMLIFLG<br>ESPRIASAAALFVYGLIPQIFAYAANFPIQKFLQAQSIVAPSAYISAATLVVHLGM<br>SWVAVYEIGLGLLGASLVLSLSWWIMVIGQYVYIVKSERCRRTWQGFTWEAFSG<br>LYGFFKLSAASAVMLCLETWYFQILVLLAGLLPNPELALDSLSICTTISGWVFMIS<br>VGFNAAASVRVSNELGARSPKSASFSVVVVTVISFIISVIAALVVLALRDVISYAFT<br>GGEEVAAAVSDLCPLLALSLVLNGIQPVLSGVAVGCGWQAFVAYVNVGCYYG<br>VGIPLGAVLGFYFQFGAKGIWLGMLGGTVMQTIILLWVTFRTDWTKEVEEAAK<br>RLTKWEDKKEPLLN*
Glyma.12G225600.1.Wm82.a2.v1	MLGIYLQGSCIISLIFSIIISIIWFYTEPILVLLHQSPDIARTAALYMKFLIPGVFAYSF<br>LQNISRFLQTQSVVMPLVALSALPLLIHIGIAYGLVQWPGLSFAGAPLAASISQW<br>ISMLLLALYVMYAKKFKQAWQGFSMHSFHYVFTNMKLALPSAAMVCIRLLVL<br>VFLAGLLPDSQITTSLIAICLNTQFIAHMVPVGLGAAERLVEEFASVTPLLAISIVL<br>DAVEGVIQGVARGCGWQHSTGLWIGLICGQLCQVGTLFLFLRRAKWTKLNLS<br>GVKDKEHPLVV*
Glyma.12G237400.1.Wm82.a2.v1	MDENGCSNEPNNKWKMPLFVFFRDARLIFKLDALSREILGIAFPAALAVVADPI<br>ASLIDTTFIGHLGPVELAAAGVSIALFNQASRITIFPLVSITTSFVAEEDTIQRLINK<br>ETETDNIENETITKENVEAPKKFKGETDESNNVVAKSTFTSGDVEKLATGNMGI<br>NNENVTSSTKSKPKVGKKRIASASTALLFGTILGLLQTAILTFAAKPLLYAMGLK<br>HDSPMLIPAEKYLRLRSIGSPAVLLSLAMQGIFRGFKDTTTPLYVIVSGYAFNVLL<br>DPILIFYLKLGLKGAAMAHVISQYMMAITLLLLLMKRVHLVPPSIKDLQIFRFLK<br>NGGLLLTRVVSVTFCMTLAASLAARLGSIPMAAFQPGLQIWLASSLLADGLAV<br>AVQTMLACSFAEKDYNKATAAATRTLQMSFVLGVGLSFAVALGLYFGPGIFSK<br>NANVVHLIKISMPFVAATQPINSLAFVFDGVNYGASDFAYSAYSLVLVSLVSIPIE<br>ILLFRSKQFVGIWIALTIYMILRMLVGIWRMGTGTGPWYYLRKGPWCYMRGHS<br>LP*
Glyma.13G203000.1.Wm82.a2.v1	MDENRSSNEPNKWKMPLFVFFKDARNVFKLDALSREILGIALPSALAVSADPIA<br>SLIDTAFIGRLGPVELAAAGVSISLLNQASRITIFPLVNITTSFVAEEDTIQKLNTK<br>AAENGNSKAKFGETIVPEDHMLQDMEKGTPKVMNTDAPTEFREDKDESQEYN<br>ATGNNDTNIGDGANTICKFSSVTSSKKSKDKVGKKKRLIASASTALLFGTILGLI<br>QAAVLIFATKPLLGVMGVKRDSPMLKPAESYLRLRSFGAPAVLLSLAMQGIFRG<br>FKDTTTPLYVIVSGYALNVILDPIFIFTLKLGIKGAAIAHVLSQYMMAFTLLLILM<br>KKVHLLPPRIKDLQIFRFLKNGGLLMLKVIAVTFCVTLATSLAARLGSIPMAAFQ<br>TCLQVWMTSSLLADGLAVAVQAILACSFTEKDYKKQQQQQQGHCK*
Glyma.13G275400.1.Wm82.a2.v1	MENVEQIKEEIEKMVASSSSEGTGVPLLRASDNNGRDQNTRSPQAEGWWNKV<br>LDMEEAKHQLLFSLPMILTNLFYHLIILVSVMLVGHLGELQLAGATLANSWFSV<br>TGVAVMVGLSGALETLCGQGFGAKEYQMLGIYLQASCIISLIFSIIISIIWFYTEPIL<br>VLLHQSPDIARTAALYMKFLIPGVFAYSFLQNILRFLQTQSVVIPLVVLSALPMLV<br>HIGVAYGLVQWSGLSFTGAPVAASISLWISLLLLALYVMYAKKFKQTWKGFSTH<br>SFRYVFTNMRLALPSAAMVCLEYWAFEVLVFLAGLMPDSQITTSLIAICINTEFIA<br>YMITYGLSAAASTRVSNELGAGNPERAKHAMSVTLKLSLLLGLCFVLALGFGH<br>NIWIQFFSDSSTIKKEFASVTPLLAISILLDAIQGVLSGVSRGCGWQHLAAYINLA

	TFYLIGLPISCFLGFKTNLQYKGLWIGLICGLLCQSGTLFLFIRRAKWTKLDLSRD<br>NDKERPLVV*
Glyma.13G275500.1.Wm82.a2.v1	MKEEIEKMVASNSSDDSIGTPLVIRGSDNNGRDQNTRLHQVEGWWNKVLDME<br>EAKCQLLFSLPMILTNTFYYLITSISVMLVGHLGELQLAGSTLANSWFNVTGSAV<br>MVGLSGALETLCGQGFGAKEYQMLGIYLQASCIISLIFSIIISIIWFYTEPILVLLHQ<br>SHDIARTTALYMKFLIPGLFAYSFLQNILRFLQTQSVVMPLVALSALPLLIHIGIA<br>YGLVQWPGLSFTGAPVATSISQWISMLLLALYVMYAKKFKQTWQGFSMHSFHY<br>VFTNMKLALPSAAMVCLEYWAFEVLVLLAGLLPDSQITTSLIAICLNTQFIAYM<br>VPVGLGAAGSTRVSNELGAGNPEQAKHAMNVTVKLSFLFSFCFALALGFGHNI<br>WIQLFSGSAKIKEEFASMIPLLAISIVLDAVQGVMQGVARGCGWQHSTVYINLA<br>TFYLVGLPISCLLGFKTNLHYKGLWIGLICGLLCQVVTLFLFLRLAKWTKLDLSG<br>DKDKDHPLVV*
Glyma.13G275600.1.Wm82.a2.v1	MLKSRSLDGTVTHFTPLLDVSHHKENTRQYRWWNSKILDLEEAKHQLLFSLPM<br>FLTNLFYYLIVLVSVIFAGHLGDLQLAGATLANSWFSVTGLAVMIGASYFNNEK<br>QDKILDLEEAKHQLLFSLPMFLTNLFYYLIVLVSVIFAGHLGDLQLAGATLANS<br>WFSVTGLAVMVGLSGALETLCGQGFGAEEYQMLGIYLQASCIISLIFSIIISIIWFY<br>TEPILVLLHQSQDIARTTSLYTKFLIPGLFALSFLQNILRFLQTQSVVNFIGAPVAV<br>SISLWISIPLLVMYIMYAERFRQTWTGFSFESFNYIFTDLKLALLSAAMVCFEYWA<br>FEIMVFLAGLLPDPTISTSLIAICTNTELIAYLITYGLSAAASTRVSNELGSGHLDR<br>AKHAMGVSLKLSLLLGLCFVLALAFGHNIWIQMFSDSSKIKEELASLTPFLSISIL<br>LDSVQGVLSGVIRGCGWQHLAAYVNLATFYLIGLPISGLLGFKFNLQVKGLWIG<br>LICGLACQTGTLSFLAWRAKWTKLNVSLDRDEIQPPSA*
Glyma.13G339800.1.Wm82.a2.v1	MQDIGAPSVSLQLCITSLNKLPLFVWFLKQHNSEFGVSDMAEKESIHSFGDWRR<br>IPICTFFQDARLVFKADSLGREILSIALPAAMALTADPIASLVDTAFIGQIGPVELA<br>AVGVSIALFNQVSRIAIFPLVSVTTSFVAEEDTLSGANPQTEEGRCLEAGQPTDTE<br>TKELLPQKVTGGNVHNSDFVGESFNIAKEERKRRHIPSASSAIFIGGILGLIQAIFL<br>ISAAKPLLNFMGVTSDSPMLHPAKQYLKLRSLGAPAVLLSLAMQGVFRGFKDT<br>KTPLYATVAGDVTNIALDPLFMFVFRLGVSGAAIAHVISQYLISVILLWRLLEQV<br>DLIPPSINHLQLDRFLKNGFLLLMRVIAVTFCVTLAASLAARQGPTSMAAFQVC<br>LQVWLAVSLLADGLAVAGQAILAGAFANKDFNRATATASRVLQMGLVLGLA<br>LAFILGTGLHFGAKIFTQDANVLHLIQIGIPFIAVTQPLNSLAFVFDGVNFGASDF<br>AYSAFSMVVVAILSIISLLILSSAGGFIGIWVALTIYMGLRAFAGFLRIGTGSGPWE<br>FLRSS*
Glyma.14G002000.1.Wm82.a2.v1	MGVVEIYKRKTLQSSANNFNCRGRRYILWAAALMLMEAIHFTTSQYQLQYHG<br>GFTRIPFLRKRNHISFGYGHHPHHHFLRHSPSLSSPFIALGSKPSSYTSEDQHHSD<br>SASVDVKRELISLTLPALASQAIDPLAQLMETAYIGRLGTVELASAGVSISIFNIIS<br>KLFNIPLLSVATSFVAEDIAKSSSAADAKTKQQLSSVSTALLLALALGFFEALALY<br>LGAGAFLHLIGVPTQNPTYVPARHFLSLRAVGAPAVVLSLALQGIFRGFKDTKT<br>PVICLGIGNFSAVFLFPLLMYYFRLGVTGAAISTVISQYIGTMLMIWCLNKRAELL<br>PPKMGDLQFGSYIKSGGFLLGRTLAVLSTMTLGTSIAARHGPVAMAAHQICMQ<br>VWLAVSLLTDALAASGQALIASSVSRHEYKVVKEVTSFVLRIGLVMGICLTAILG<br>ASFGSLATIFTQDSEVLQVVKTLALFVSASQPFNALAYIFDGLHYGVSDFRYAAF<br>SMMFVGAVSSAFLVFAPPLFGLQGVWLGLVLFMALRAAAGAVRLLSKNGPW<br>WFLHRDLQIAEVVS*

Glyma.14G032400.1.Wm82.a2.v1	MSSLEHQPLLPRLDSHSHIQNLSSDAIEEFLEHRPIALRWWSKLIVWESRLLWLL<br>SGASIVVSIFNYMLSFVTLMFTGHLGSLELAGASVASVGIQGLAYGIMLGMASA<br>VQTVCGQAYGAKKHGAMSIILQRAIILHIGAAVILSFLYWFSGDFLKAIGQSDSI<br>AERGQVFARGIILQLYAFAISCPMQRFLQAQNIVNPLAYMSVGVFLVHILLSWL<br>VIYVLGYGLQGAALTLSFSWWLLVLFNGLYIIFSPRCKETWAGFSVKAFKGIWPY<br>FKLTVASAVMLCLEVWYNQGLVLLSGLLSNPTISLDSISICMNYLNWDMQFML<br>GLSTAASVRVSNELGASHPRVAKFSVFVVNGTSILISVVFCTIILIFRVSLSKLFTSD<br>SDVIDAVSNLTPLLAISVFFNGIQPILSGVAIGSGWQALVAYVNLASYYVVGLTV<br>GCVLGFKTSLGVAGIWWGMILGVLIQTVTLIILTARTNWQAEVEKAVVRINKSA<br>ENDTLDQLVADT*
Glyma.14G078000.1.Wm82.a2.v1	MDSPLLENIDNSTTYVEEKKTLNSVVKKFGFESKKLWKIAGPAILTSICQYSLGA<br>LTQTFAGLVGDLELAAVSVENSVVAGLAFGVMLGMGSALETLCGQAYGAGQS<br>TMLGVYMQRSWVILFVTALILLPLYIWSPPILRLFGQTAEISDAAGKFALWMIPQ<br>LFAYAINFPMVKFLQAQRKVLVMLWISVVVLVLHTFFSWFLIFKLGWGLIGAAI<br>TLNTSWWVIVIAQLLYIFITKSDGAWNGFTWLAFSDLFGFVKLSLASAVMLCLE<br>FWYLMILVVITGRLKNPLVPVDAISICMNINGWDAMIAIGFNAAISVRVSNELG<br>AGDFKAAKFSVWVVSITSVFIGVVAMIGVLSTKDYFPYLFTTSVPVANETTRLAA<br>LLGVTVLLNSLQPVLSGVAVGAGWQSLVACINIVCYYVIGLPAGIILGFPLGLGA<br>EGIWSGMIAGIVLQTTILIIVTSIRNWKKEAEEAESRVKKWGGAIAYDQ*
Glyma.14G158100.1.Wm82.a2.v1	MLGVYMQRSWVIVNATAILLSLLYIFAGPMLRAIRQTEAISAAGGEFAVWMIPQ<br>LFAYAVNYPAQKFLQAQSRIMVMAWIAAAALVLHTLFSWLLILEFWWGLVSA<br>VVVLNASWWFIDIGQLHTFSSIL*
Glyma.15G034600.1.Wm82.a2.v1	MPNIGAPSVSLQLCITSLNKLPLLVWFLKQHSEFGFSNMAEKESMYSLGDWRRI<br>PICTFFKDARLVFKADSLGREILSIALPAAMALTADPIASLVDTAFIGQIGPVELA<br>AVGVSIALFNQVSRIAIFPLVSVTTSFVAEEDTLSGENPHIEEGRCLETGPPKDAE<br>TKELLPHKVTGGNNHNSDFVGECFNIAKEEHKRRHIPSASSAIFIGGILGLIQAIF<br>LISAAKPLLNFMGVTSDSPMLHPAKQYLKLRTLGAPAVLLSLAMQGVFRGFKD<br>TKTPLYATVAGDVTNIALDPLFMFVFRLGVSGAAIAHVISQYLISAILLWRLMEQ<br>VDLVPPSIKHLQLDRFLKNGFLLLMRVIAVTFCVTLAASLAARQGPTSMAAFQV<br>CLQVWLAVSLLADGLAVAGQAILAGAFANKDFDRATATASRVLQMGLVLGL<br>ALAFILGIGLHFGAKIFTQDANVLHLIQIGIPFVAVTQPLNSIAFVFDGVNFGASD<br>FAYSAFSMVVVAILSIICLLILSSAGGFIGIWVALTIYMGLRAFAGFLRIGTGSGPW<br>EFLRSS*
Glyma.15G107100.1.Wm82.a2.v1	MMESQNQNLLRQPLINSTHHHHHSADSRLEEVLSDPTLPWSKRILSATWIELNL<br>LFPLAAPAILVYVFNNLMSNVTRAFAGHLGNLELAAANLGNSGIQLFAYGLML<br>GMGSAVETLCGQAYGANKYEMLGIYMQRAIIVLTITGIPLTVVYIFCKPILLLLGE<br>PPEVASVAAMFVYGLIPQIFAYAVNFPIQKFLQAQSVVAPSTYISAATLVLHVAL<br>SWVVVYKLGFGIMGSSLMLSLSWWIIVGAQFLYVVSASKFKDTWSGFSVEAFSG<br>LWDFVKLSAASAVMLCLETWYFQVLVLITGLLDNPQLSLDSISVCMAITGLTMH<br>IGIGFNAAASVRVSNELGAEHPKSAAFSVIVVNMISFIIAVIEAVVVLALRRVVSY<br>AFTDGETVANAVSDLCPYLAVTLILNGIQPVLSGVAVGCGWQAIVAYVNVGCY<br>YGIGIPLGCVLGFTFGLGVQGIWSGMIGGTMLQTLILLWITLRTDWNKEVNTAK

	KRLNKWGYKKEPKIQS*
Glyma.15G150300.1.Wm82.a2.v1	MVAEEKSQKTYPPTAEVVDELKRMGDIGVPIAAMSLVGYIKNMVLVVCMGRL<br>GSLELAGGALAIGFTNITGFSVLSGLAMGMEPLCTQAFGSRNFSLVSLTLQRTII<br>MLLVASLPISLLWLKLEPLMLWLHQNPEITKVASVYCFFSIPDLIANSLLHPIRIYL<br>RSKGTTWPLLWCTLLSILIHIPIVAFLTFKLHLGVPGIAMSAFVANFNTLFFLLSY<br>MLYMRVSKGSLSMPLLISSRPLSSSPRQHHHQDQTSLKTTTTLGKEWGMLIRFSI<br>QSCLGVCLEWWWYEFMTILAGYLHNPRVALATAGIVIQTTSLMYTLPTALSAS<br>VSTRVGNELGAGQPERARLSTIVAIGMSLASSILGLLWTTIGRNRWGRVFTSDSE<br>VLELTMSVLPIIGVCELANCPQTTSCGILRGSARPGVGAGINFYSFYLVGAPVAIV<br>MAFVWKLGLVGLCYGLLAAQIACAVSILVVVYNTDWERESLKAKSLVGIYKSS<br>CDDQHHGDQTVKCEEGVVFLNEKE*
Glyma.15G274600.1.Wm82.a2.v1	MDENGSSSNAPKNKRNLPVSVFFKDARHVFKMDSIAKEILGIAFPSALAVAADP<br>IASLIDTAFIGHLGPVELAAAGVSIALFNQASRITIFPLVSITTSFVAEESTIEKINTE<br>KKLTDKTKSKEVMHDDHSLQDIEKGASKEKNETPTESSAVRGNTTCVPENVEM<br>DDCNTSICKSTTETSSSSNKSVSKAGRKKRHIASASTALLFGTILGLLQATTLIFAA<br>KPLLAAMGLKPDSPMLNPAIKYLRLRSLGAPAVLLSLAMQGIFRGFKDTTTPLY<br>VILSGYALNVILDPVLIFYCKLGIKGAAISHVLSQYLMALALMVILTRKVDLVPPS<br>IKDLQIFRFLKNGGLLLARVIAVTFCQTLAASLAARFGPIPMAAFQTCLQVWLTS<br>SLLADGLAVAVQAILACSFAEKDYEKVLVAATRTLQMSFVLGVGLSFAVGVGL<br>YFGAGIFSKSVLVVHLIRIGLPFVAATQPINSLAFVFDGVNYGASDFAYSAYSLVT<br>VSLASVASLFLLSKSKGFVGIWIALTIYMSLRMFAGVWRMGTGTGPWRFLRGRS<br>MS*
Glyma.16G149100.1.Wm82.a2.v1	MEETLLLPKENKRVSSNSMSSSSCSGFVQEFKKFLLQVVSLMMAGRSSWRTLPC<br>RYCIGHFLCRLHDNNSFLLSLMGMAGALETQCGQSFGTEQFHKLGNYVFCAIL<br>FLILSSAPISILWIFMDKLLVLLGQDHAISLVAGNYCIWLIPTLFGYSRFGSLFSDSE<br>LDLSNACNLSCCFSFAYTYLLAALSIGISYWLSVMLLIVYTQCFKKHQRVFLLSH<br>PICTYDLLSGGRSLELLVIFAGLLPNPKLETSVLSIWCVSP*
Glyma.16G157300.1.Wm82.a2.v1	MGDNKDHDFFSHKFPTTSQVMEEMKELWGMALPITAMNMLVFVRAVVSVLF<br>LGRLGSLELAGGALSIGFTNITGYSVLVGLAAGLEPVCSQAFGSKNWDLLSLSLQ<br>RMVLILLMAIVPISLLWLNLERIMLFMGQDSAITGMASLYCFYSLPDLLTNTLLQ<br>PLRVFLRSQKVTKPMMYCSLVAVLFHVPLNYLLVVVMGLGVPGVAMASVMT<br>NLNMVVLMAGYVCVCRKREVVLKWRCGGGGVVCSGLGQLMGFAVPSCLMIC<br>LEWWWYEIVTVLAGYLPRPTLAVAATGILIQTTSMMYTVPMALAGCVSARVG<br>NELGAGKPYKAKLAATVALGCAFVIGFINVTWTVILGQRWAGLFTNDEPVKA<br>LVASVMPIMGLCELGNCPQTTGCGILRGTARPGIGAHINLGSFYFVGTPVAVGL<br>AFWFKVGFSGLWFGLLSAQVACAVSILYVVLVRTDWEAEALKAEKLTRIEMGS<br>CNGLRNKENERDEESKGLLVNGNGNNKDDTC*
Glyma.16G180000.1.Wm82.a2.v1	METPLVIQKHTSEPDYLPVESLKDVMFVLRTETVKIWRVALPMALLALFQLLMD<br>SSTSIYAGHIGDIELSSIGVYQGVIGAIYFYLLFGMSSALATLCGQAFGAGKIQSTC<br>IYVQRSWIILTATCIILLPIYVYATPILKLLGQDEGIAEVAGRYSIQVIPYMFSFAVA<br>FPIQRFLQAQSKVKVIMCIAFVDLLIQNGLLYIFINVFGWGITGLAIVTNIVGWLY<br>AVALVVYTIGWCKEEWSGFCWMAFRDLWAFAKLSLASSVMNCLEQWYITCIM<br>LLAGLLDNPVIAVGSYSICFNVQGWDDMLRLGINTAISVRVSNTLGMSHPRAAI<br>YSFCVTMFQSLLLGILFMTVIFFSKDEFAKIFTDSEDMILAAADLAYLLGVTIVLN

	SASQVMSGVAIGSGWQVMVGYINLACYYIVGLPIGIFLGFKLHLGVKGLWGGT<br>MCGSILQTLVLFTIIWKTNWSKEVEQTAHRMRLYVEQ*
Glyma.<br>16G180<br>100.1.<br>Wm82.<br>a2.v1	MYGWICVAKAEVCQPRCPGASTPYSLVLMDSSTSIYAGHIGDIELSSIAVYQAFR<br>IQRFLQAQSKVKVIMCIAFVDLLIQNGLLYIFINVFGWGITGLAMVTNIIGSLCAL<br>ALVVYTIGWCREEWRGFSQLQKRNLWAFAKLSLASSVMNCLEQCFNVQGWD<br>YMLLLGINTAISLKDVKFVLWTETVKIRRITLPVALSQLFQFLTNSSTSIYVGRVG<br>DIELSSISVYQGVISSI*
Glyma.<br>16G180<br>200.1.<br>Wm82.<br>a2.v1	METPLVIQKFTSESDYLPVKSLKDLKFVLWTETVKIWRIAFPMALSALLQFLTISS<br>TSIYAGHLGDIELSSISVYQGVISAIYFDLLFGMSSALVTLCGQAFGAGQIQSTCIY<br>VQRSWIILTATCIILLPIYVCATPILKFIGQDHEIADLAGRYSIQVIPYMFSCAITFPF<br>QTFLQAQIKVKVITCIALAVLVIQNVLLYIFINVFGWGTTGLAMVTNITGWVYA<br>MALVVYTIGWCKEEWTGFSWMAFRDLWSFAKLSLASSVMSCLEQWYGTCIILL<br>AGLLDNPVIDVGSYSICFNVQGWHTMLLLGISVAISIRVSNTLGMSHPRAAIYSF<br>CVTMFQSLLLGIVFMIAIFLSKDEFAKIFTDSEDMIRAVADLAYLLGVSMVINSAS<br>QVMSGVAVGSGWQVMVGYINLACYYVVGLPIGIFLGFNQHLGVKGLWGGTM<br>CGRILQMLVLLIIIWKTNWSKEVEQTAHRMRIWSINNLHSNDMGNVT*
Glyma.<br>16G198<br>700.1.<br>Wm82.<br>a2.v1	MCHPTLDVTCNCQCCSTSPPDCPISITNPLISKPQNTTTPPPPPHQHNNTTTKTK<br>TPPPSTTATKTTTAPKPHHVPEAIKEVFSISKIVIPMILTGLLLYCRSMISMLFLGH<br>LGELALAGGSLAIGFANITGYSILSGLAVGMEPICGQAFGAKRFTLLGLCLQRTIL<br>LLLFTSLPISLLWLYMKQILLLCGQDVAIATQAQSYLVYSIPDLIAQSFLHPLRIYL<br>RTQSITLPLTLCASFSILLHIPINYFLVAHLKLGIKGVALGGVLTNFNLVASLILYIV<br>FSGTHKKTWGGFSFECFTQWKSLLNLAIPSCVSVCLEWWWYEIMILLCGLLVNP<br>KATVASMGILIQTTSLLYIFPSSLSFSVSTRVGNKLGAQKPSKARLSAIVGLSCSFM<br>SGVLALVFALMVRNTWASMFTKDKDIITLTSMVLPIIGLCELGNCPQTTGCGVL<br>RGTARPKVGANINLGCFYLVGMPVSIWLAFFTGYDFQGLWLGLLAAQGSCAV<br>TMLVVLCRTDWEFEAQRAKKLTGMGGAASGVDQSREVDPEKPLKHESNEDSL<br>LLADSDENEQ*
Glyma.<br>17G027<br>900.1.<br>Wm82.<br>a2.v1	MLAEQKFQKTYPTVTEVLEEVRRMTDIGFPIAAMSLVGYLKNMTLVVCMGRLG<br>SLELAGGSLAIGLTNITGYSVLSGLAMGMEPLCTQAFGSRNLSLLSLTLQRTILM<br>LLLFSLPISLLWLNLESLMLCLRQNPDITRVATLYCRFAIPDLIANSFLHPLRIFLR<br>SKGTTWPLLWCTLLSILLHLPTLTFLTFKLNLGVPGIAISSFVANFSNLFFLLLYMF<br>YTRVPKESLHVSLLMSHNNLIITCSSSTSTIAKEWGMLMKFSIQSCLAVCLEWW<br>WYELMTISAGYLDNPRVALATAGIVIQTTSLMYTLPTALSASVSTRVGNELGAG<br>QGERANLSTVVAIGLALVSSIFGLLWTTMGRERWGRVFTSDSEVLQLTVAVLPII<br>GLCELANCPQTTSCGILRGSARPGIGAVINFCSFYLVGAPVAIVLAFYWKLGMV<br>GLCYGLLAAQIACVVSIFGVVYKTDWERESLKARCLVGKASCGTFAYDEDQTD<br>ALKCEEGVVFLNSQK*
Glyma.<br>17G131<br>700.1.<br>Wm82.<br>a2.v1	MLTTLIPKSPTCQLHQMQNPHNNINTTHLSLSLVEAKCIANISFSMILTGLLLYS<br>RSVISMLFLGHLGELALAGGSLAIGFANITGYSVLSGLAMGMEPICGQAFGAKR<br>FKLLGLTMQRTVLLLLITSCLISLFFWLNMKKILLLCAQEQDIANEAELYIFYSLP<br>DLVLQSLLHPLRIYLRSQSITLPLTCCAAVSILLHVPVNYLFVSILNLGIKGVALSA<br>VITNLNLVVLLIIYIVFSGTHKKTWPGISRECFNGWKKLLNLAIPSCVSVCLEWW<br>WYEIMILLCGLLVNPHASVASMGVLIQTTALIYIFPSSLSFGVSTRVGNELGAGN<br>PRRAKLAAIVGLCFSFVFGLSALAFAVSVRNVWASMFTLDGEIIALTTAVLPIIGL

	CELGNCPQTTVCGVLRGTARPKLGANINLGCFYLVGMPVAVWLGFFAGFDFKGLWLGMLAAQGSCIVTMMFVLARTNWEGQALRAKELTSSDPREEKKLVEEEHEEKGFLGSCATKEASDLVV*
Glyma.17G136400.1.Wm82.a2.v1	MSATSKGESITANDWSNRQPSFPSSHTQKCPSNANNFVKMVVEELRVQRGIALPLVPMNLAWFAKLAITTAFLGHLGELNLAGGALGFSFANVTGFSVLNGLSGAMEPICGQAHGAKNARLLHKTLLMTTLLLLLVTLPLSFLWLNLGKILILFGQQQEISTVAKTYYVSNLIPDLFIKALLCPLKAYLSSHCVTLPTMFSSAVALAFHIPVNIVLSKTMGLRGVAIAVWITDLMVMVMLAIYVVVLERRSEGLLWKEGGWWDQNMMDWIRLIKLSGSCCLNTCLEWWCYEILLFLTGHLANAKQANFDYLHYAVMISLATSVSTRVSNELGANRAGQACESARVSLALSVVSGCIGGSTMVAARGVWGDLFSHDKGVVKGVKKAMLLMALVEVFNFPVTVCGGIVRGTGRPRLGMYASLGGFYFLTLPLGVVFAFKLRLGLAGFTIGLLIGIVACLILLLTFIVRINWVQEATKAQTFVCIAQVQEQVPRYEVNELVENHENDQV*
Glyma.17G136500.1.Wm82.a2.v1	MSATSKQVENLSTNDKANHRPSSPSSHTKKCPFNANFIKMVLEELRVQRGIALPLVAMNLAWFAKTAITTAFLGHLGELNLAGGALGFSFANITGFSVLNGLSGAMEPICGQAHGAKNVRLLHKTLLMTTLLLLLASLPISFMWLNVDKILICFGQQQEISTVAKTYVSYLIPDLLVTSLLCPLKTYLSSQCMTLPTMFSSAVALAFHIPVNIVLSKTMGLRGVSIAVWITDLMVMVMLAVYVVVLERRNEGMLWKEGGWWDQNVMDWIRLMKLSGSCCLNTCLEWWCYEILVLLTGHLANAKQALGVLAIVLNFDYLLYSVMLSLATCVSTRVSNELGANSAGQAYKSARVSLAVGVISGCIGGSMMVASRGVWGNLFSHDKGVVKGVKKTMFLMALVEVFNFPVTVCGGIVRGTARPWLGMYANIGGFYFLALPLGVVFAFKLRLGLAGLIIGFLIGVVACLILLLTFIVRINWVQEATKAQMLVCIPAQVQEQVPRNQVNELVR*
Glyma.17G175100.1.Wm82.a2.v1	MGLITYGKSAISTYFLGKLSKEALVGGSLAIGVANITGYSIISSLATSMDGISSQACGAQQWTLIGQTLQCSIMILTLTCITISILWLNIEPVLLFCGQNPTISSIATTYLGFSLPDLIFTSLIISFKIFLRTQDVTLPFMFSATLAPFLHAIINNVVIHTFGLGIQGVALVGSFTNIKFLIILLLYLWFSRNCSNSWQGWWYELLVLFSGVLPNATKTIATYGIIIQATSLIYNFPYALSLAVSPKVGNELGANRSDKAKASSFYALLCAFITTIVATILTVNGSARPTLGAKINLVSFYVVGLPVALLMSFVFDLGLLGLLLGLLLAQIVRASVMTIVLARTNWGASTEGKGV*
Glyma.17G247400.1.Wm82.a2.v1	MDSPLLENIDNSCSNVEEEKTPNTVVKRFGFESKKLWKIAGPAIVTSICQYSLGALTQTFAGLVGDLDLAAVSVENSVIAGLAFGVMLGMGSALETLCGQAYGAGQIRMLGVYMQRSWVILFITALILLPLYIWSPPILRLAGQTAEISDAAGKFAVWMIPQLFAYAINFPIVKFLQAQRKVLVMLWISVVVLVLHTFFSWLVIFKLGWGLIGAAVTLNTSWWVIVIAQLLYIFITKSDGAWSGFTWLAFSDLFGFVKLSLASAVMLCLEFWYLMILVVITGRLENPLIPVDAISICMNINGWDAMIAIGFNAAISVRVSNELGAGDFKAAKFSVWVVSITSVSIGVVVMIGVLLTKDYFPYLFTTSVPVANETTRLSALLAVTVLLNSLQPVLSGVAVGAGWQSLVAYINIVCYYLVGLPAGIILGFKLGLGAEGIWSGMIAGIVLQTTILIIVTSIRNWKKEAEEAESRVRKWGGAISYDQ*
Glyma.18G097600.1.Wm82.a2.v1	MIVIFLSKDEFAKIFTNSEDMIRAVADLAYLLGVSIMSGVAVGSGWQVMVGNINLACVYYVVGLPIGIFLGFNQHLGVKGL*

Glyma.18G110800.1.Wm82.a2.v1	MDKLLVLLGQDHAISLVAGNYCIWLIPALFGYVVLQALVRYFQTQSLIFPMLVT<br>SVVVLVLHIPICWVLVFELGLGQNEAALSIGISYWLSVMLLIVYTVLSVMSENLG<br>SFR*
Glyma.18G143700.1.Wm82.a2.v1	MEDHEDHTHPLLTPRHHTPDPAVVFTAKSDDIAPIAGAGDFAREFFAESKKLW<br>YLAGPAIFTSVCQYSLGAVTQVFSGHVSTLALAAISIENSVIAGFCLGITFGMGSA<br>LETLCGQAYGAGQVHMLGVYMQRSWVILNATAILLTLLYIFAAPLLRAIGQTE<br>AISAAAGDFAVWMIPQLFAYAVNYPAQKFLQAQSRIMVMAWIAAAALVLHT<br>VFSWLLMLKLRWGLVGAAVVLNASWWFIDLAQLVYIMGGACGEAWSGFTFK<br>AFHNLWGFVRLSLASAVMLCLEVWYFMALILFAGYLKNAEVSVDALSICMNIL<br>GWTIMVSFGMNAAVSVRVSNELGACHPRTAKFSLLVAVITSTLIGVMLSMVLIIF<br>RNQYPFLFSNDSEVRKIVVELTPMLALCIVINNVQPVLSGVAVGAGWQAVVAY<br>VNIACYYFFGIPLGLILGYKLDKGVMGIWSGMLLGTILQTCVLFFMVYRTDWNK<br>EASLAEDRIKQWGGHEDSKTHAIAKNDQET*
Glyma.18G206500.1.Wm82.a2.v1	MVSSLSTFFLCTHSLKSPTLKPLTKFYCFARLRFRVPPKASSRNKNANTDSVETPS<br>LQPSSLKPPYDSSSSLLRCFSGVLKFVYWVLASDGWLTFDESVIVLIALPAALALA<br>ADPIASLIDTAFVGHIGAVELAAVGVSASGFNLVSKAFNVPLLNVTASFVAEEQ<br>ALIRKEEESIPSDKNDFPFQMYIQIKLLPSVSTSLALAATLGMAETVVLTLGSGIL<br>MNIMGIPADSPMRGPAEQFLTLRAFGTPAIVLALAAQGTFRGFLDTKTPLYAVG<br>KVHNLKFEITNKYLLTEMFLGVGNFLKAILDPILIFLFGLGGATVATLISEYLIAFI<br>LLWKLSDKVLLIPSEFYGRKFFSYLKSETLLFDSNWILLCLEPAIPIAEVFKDIPNIP<br>PPPIPKMFLTLAVFITVMLSTSVAAQQGPIPPMAGHQICMQVWLSALLACNYSL<br>GNYEQASLVIFRVMQIGLGAGITLSMILFFGFGAFSSLFSTDSEVLDVARSGIWFV<br>AGSQPVNALAFVIDGIYYGVSDFGYAAYSMVISYHVGLVSSTFLLVVAPVGLPG<br>VWTGLFIFMALRVLAGLWMLSSKSGPWDTFWYKDGAED*
Glyma.18G214800.1.Wm82.a2.v1	MRSTKGGDGEGKRNCGCCGRFIEGLHQHLHQFLKALFPTLSLSEVKEELKSLA<br>NIACPMMMTNVLLYSRSAISMLFLGRQGKVELAGGSLAIGFANITANSFLKGLT<br>MGMDPICCQAYGAKRWSVLSQTFCKTLCLLLLVAIPISLLWLNMAPLLHWLGQ<br>DPEVTKVAQVYMVFSIPELLAQVHLNPLRSFLRTQGLTTPLTIAASFAAILHLPIN<br>YFLATYLELGVKGIALATGLNSINMILGLVLYLLVSKKPLKPWEGATILSSFHDW<br>RPLLTLALPSCISVCLEWWCYEIMLFLCGLLSNPQTTVATMGVLIQTTGFLYVFP<br>FSLSAALTTQIGHSLGAGQPSRAQITATIGLFIAFALGFSAFVFLLIVRNVWGKLF<br>TNETQIVDMVTTILPILGLCEIGNWPQTAACGILSGTARPYVGARINLCAFYLIG<br>LPVAVFAAFMHRYQLRGLWFGMLAAQISCFCMMVYTLVQTDWGHQSRRAEQ<br>LAQATDEEISGFLDSDM*
Glyma.18G235200.1.Wm82.a2.v1	MFNNGFNSMETTELHHAPEGLVDTNGGDYTEMSGFADFKNVFSVESIKLWTIA<br>APIAFSILCNYAVNSFTTIFVGHLGDLELSSVSLSLSVVSNFSFGFLLGMASALETL<br>CGQAFGAGQVEMIGVYMQRSWLILLGACICLTPIYIYAEPILLLLGQEPEIAELA<br>GAFTIQSIPQMFSLAINFPTQKFLQAQTKVGFLAWLGFGAFIFHVILLWILLKVFS<br>LGTTGAAVAYCTTAWIIALAQTAYVIGWCKDGWRGFSWLAFKDLWAFVKLSV<br>ASAVMLCLEIWYFMILIVLTGHLDNAVIAVGSLSICMTINGFEGMLFIGINAAISV<br>RVSNELGSGRPRAAKYSVIVTIIESLIIGLISAAIILATKDHFAIIFTESKEMIKAVSK<br>LAGLLGITMILNSVQPVISGVAVGGGWQALVAYINLFCYYIMGLPLGFLLGYKL

	GYRVEGIWVGMICGTMLQTLILLYIVYKTNWNKEVEQASERMRKWTGQEIEIN<br>LANSQQTFTR*
Glyma.18G293100.1.Wm82.a2.v1	MEEGSETGKWGWMKRRRAMREELKKVGTIAAPMVVASVLQYLLQVVSLVMV<br>GHLNQLSLSSVAIAISLTNVSGFSVLSGMAGGLETLCGQAFGAGQYEKFGQYTY<br>TAVISLSLICFPITILWTFMDKILTLLGQDPTISLEARKYAIWLIPALFGSAILKPLTR<br>FFQTQSLISPMILTSAIALCFHGATCWTLVFKLELGHVGAAISFSLCVWFNVMLL<br>LSFVRYSSACEKTRIPFSKNALVGVGVFFRFAVPAAVMVCLKWWACEILVLLAG<br>LFPNPKLETSVLSICLTISTLHFTIPYGFGAAASTRVSNELGAGNPQAVRVAVSAT<br>MFLAVTEGLIVSATLFGCRHLLGYAYSDDRMVVHYVAVMTPLLCLSIFTDSLQG<br>VLSGVARGSGWQHLGAYVNLGAFYLVGIPVGIVLGFVAHLRAKGLWIGIVTGSI<br>VQSILLSLVTALTNWKKQAMMARERIFDAKPPDENESNHMTSA*
Glyma.18G293200.1.Wm82.a2.v1	MAAPMLAANMCQYLLQVVSLMMVGHLGLLVSFSGVAIAISFAEVTGFCVLMG<br>MAGALETLCGQTYGAEEFTEIGNYTFCAIVTLLLVCLPISLLWIFMDKILLLFGQ<br>DPEISHVAHKYCICSIPALYGFAVLQCQIRYFQTQSMIFPMVFSSIAVLCLHVPIC<br>WGLVFKLGLGHVGAAYAIGISYWLNVIGLGIYMNYSPACEKTKIVFSFNALLSIP<br>EFCQFAIPSGLMFCFEMWSFELLTLFAGLLPNPQLQTSVLSVCLNTTTLHYIIPYA<br>VGASASTRISNELGAGNPKAAQDIVPILCGSFTADSLIGALSGIARGGGFQQIGA<br>YVNLGAYYLVGVPLAFLLGFVLHFNAKGLWMGSLTGSVLQVIILTVVTVLTDW<br>QKEATKARERIVEKSIKVHNDSVI*
Glyma.18G293300.1.Wm82.a2.v1	MEETLLLPKENKRVSSNSKSSSSGSGFVQEFKKVSLMAAPMVVVSVSQFLLQVV<br>SLMMAGIALATSFADVTGFNILMGMAGALETQCAQSFGTEQFHKLGNYVFCAI<br>LFLILSSAPKSILWIFMDKLLVLLGQDHAISLVAGNYCIWLIPALFGYAVLQALV<br>RYFQTQSLIFPMLVTSVVVLVLHIPICWVLVFELGLGQNEAALSIGISYWLSVMLL<br>IVYTLSGGRSLELLVILAGLLPNPKLETSVLSICLKICNLHYFIPYGTGAAVSSRVS<br>NELGAGRPQAAREAVFAVIVLTFTDAIVFSSVLFCFRHVLGFAFSNEMEVVHSV<br>AKIVPVLCLSFSVDGFLGVLCGSRLQKMGAISNLVAYYAVGIPVSLVFGFGLNFY<br>GKGL*
Glyma.19G001600.1.Wm82.a2.v1	MESSREMNKELAAPLLVPRKSGDGQENNNNNGVEVVASSSESTFCQELKRVSS<br>MAAPMVAVTVSQYLLQVVSLMMVGHFGILVSFSGVAIATSFAEVTGFSVLLGM<br>SGALETLCGQTYGAEEYRKFGNYTWCAIVTLTLVCLPISLVWIFTDKILLLFSQDP<br>EISHAAREYCIYLIPALFGHAVLQALTRYFQTQSMIFPMVFSSITALCLHVPICWG<br>LVFKLGLGHVGAALAIGVSYWLNVVWLAIYMIYSPACQKTKIVFSSNALLSIPEF<br>LKLAIPSGLMFCFEWWSFEVLTLLAGILPNPQLETAVLSICLNTTTLHYFIPYAVG<br>ASASTRVSNELGAGNPKTAKGAVRVVVILGVAEAAIVSTVFISCRHVLGYAYSN<br>DKEVIDYVAEMAPLLCVSVTADSLIGALSGIARGGGFQEIGAYVNLGAYYLVGI<br>PMGLLLGFHLQLRAKGLWMGTLSGSLTQVIILAIVTALIDWQKEATKARERVVE<br>NSIKAHI*
Glyma.19G120200.1.Wm82.a2.v1	MEMEEELKETLMHHPNNTSLESVEKEESLRKRAWEESKKMWVVAGPAIFTRFS<br>TFGIMVVSQSFIGHIGSTELAAYAIVMTVLVRFANGVLIGMASALDTLCGQAYG<br>AKKYDMLGVYLQRSWIVLFMTSILLLPIYIFTTPLLEALGQDKTIAQVAGSISLWS<br>IGIIFAFSVSFTSQMFLQSQSKNKIIAYLAAVSISIHVLLSWVLTVQFKFGLNGAMT<br>STLLAYWIPNIGQLVFIMTKCPDTWKGFSFLAFKDLLPVIKLSLSSGAMLCLEIW<br>YNTVLILLTGNMKNAEVSIDALAICLNISGWEMMIALGFFAAASVRVANELGR<br>GNSKATKFSILITVLTSFSIGFVLFLVFLFLRGKLAYIFTPDPEVAKAVGDLSPLLSF

	SFLLNSVQPVLSGVSVGAGWQSVVAYVNIGCYYLIGIPVGVLLDNLFHLEVKGIWIGMLFGTFVQTVMLITITFKTDWDKQVEIARNRVNKWAVTTENEESNSRSSISS*
Glyma.19G120300.1.Wm82.a2.v1	MEGHLKQKLLRNKERRTSSEQEEEELSLVKRVWNESQVMWIVAAPAIFTRFSTFGISVISQAFVGHIGSKELAAYALVFTVLVRFANGVLLGMASALSTLCGQAYGAKEYGMMGVYLQRSWIVLFLTAVCLLPVFIFTSPILMLLGQDESIAQVAGNIALWSIPVMFASIVSFTCQTFLQSQSKNVIIAFLAAFSIVIHVFLSWLLTMKFQFGIPGAMISAGLAYWIPNIGQLIFVTCGWCSDTWEGFSFLAFKDLWPVVKMSLSAGAMLCLELWYNTILVLLTGNMKNAEVEIDALSICLNINGWEMMISLGFMAAASVRVANELGRGSAKAAKFSIIVSVLTSLAIGFLLFLFFLFFRERLAYIFTSNKDVAFAVGDLSPLLSVSILLNSVQPVLSGVAIGAGWQSIVAYVNMGCYYAIGIPVGIVLGNVLDLQVKGIWIGMLFGTLIQTIVLIVITYKTNWDEQVTIAQKRISRWSKVDNADQENEAQRKYVS*
Glyma.19G120700.1.Wm82.a2.v1	MSSALSTLCGQAYGAKEYAMMGVYLQRSWIVMSLTTLFLLPVFIFTRPILMLLGQDEIIAEVAGTISLWSIPIIFAFIASFTCQNFLQSQSRNTIIALLAAFSIVIHVFLSWLLTIQFKLEIPGAMTSTSLAFWIPNIGQLIFITCGWCSDTWKGFSFLAFKDLWPVVKLSLSSGVMLCLELWYNTILVLLTGNMENAEVQIDALSICLNINGWEMMISLGFMAAASVRVANELGKGSSKAAKFSIVVTVLTSLAIGFVLFLFFLFLREKLAYIFTTNKDVAQAVGDLSPLLAISILLNSVQPVLSGVAIGAGWQSIVAYVNIGCYYIIGVPVGVLLGNVLNLQVKGIWIGMLFGTFILTVVLIVITYKTDWDKQVIIARNRINKWSKVESDHEIITSDN*
Glyma.19G120900.1.Wm82.a2.v1	MEGNLEKKLLSREKKSEEEEEEEKLSLVKRVWEESKVMWIVAAPAIFTRFTTFGISVISQAFIGHIGSRELAAYALVFTVIIRFANGILLGMASALSTLCGQAYGAKEYDMMGVYLQRSWIVLFLTAICLLPLLIFTSPILTILGQDESIGQVAGTISLWSIPILFAYIVSNNCQTFLQSQSKNVIISFLAALSIIIHVSLSWLFTMQFKYGIPGAMISTILAYWIPNIGQLIFITCGWCPETWKGFSVLAFKDLWPVAKLSISSGAMLCLEFWYSTILILLTGNMKNAEVQIDALSICININGWEMMIAFGFMAAASVRVANELGRGSSKAAKFSIVVTVLTSFVIGFILFLLFLFLREKVAYLFTSNEDVATAVGDLSPLLAVSLLLNSIQPVLSGVAVGAGWQSTVAYVNIGCYYLIGIPVGIVLGNIIHLQVKGIWIGMLFGTLIQTIVLTIITYKTNWDEQVIIARSRINKWSKVESDHETTTSDN*
Glyma.19G244000.1.Wm82.a2.v1	MEQVDLVPPSIKHLQLDRFLKNASLATRQGPTSRAAFQFIASKITCWFHAILAGAFANKDFDRATTTASRVLQICLILGLALAFILGIGLHFGAKIFTQDANVLQICLILGLALAFILGIGLHFGAKIFTQDANVLQICLILGLTLTFILGIGLHFGAKIFTQDANVLHLIQIGITFVVVTQPLNSLAFVFYGVNFGASDFAYSAFSMVRLLHYGNSQVVVAILSIICLLILSSAGGFIGIWVALTIYMGLRAFASFLRIGMGSGPWEFLRSS*
Glyma.20G123300.1.Wm82.a2.v1	MWIPHYFSSFLRKEEQPNGGRKMNLTFFVSSPFFPSFLAGTKEREAEYVMRWSVFGEEMKRVGYLAGPMINVTLSQYFLQIISMMMVGHLGKLVLSSTAIAISLCAVSGFSLIFGMSCALETQCGQAYGAQQYRKFGVQIYTAIVSLTLACLPLTLLWVYLGKILIFLGQDPLISQEAGKFALCMIPALFAYATLQALVRYFLMQSLTSPLFISSSITLCFHVAFCWLLVFKCGFGNLGAAFSIGTSYWLNVVLLGLYMKFSTECEKTRVPISMELFHGIGEFFRCAIPSAGMICLEWWSFELLTLLSGLLPNPELETSVLSICLSVTTTIYTIPEAIGSAASTRVSNALGAGSPQSAQLSVSAAMTLAASAAILVSSIIFACRQVVGYVFSSELDVVDYFTDMVPLLCLSVILDTLHGTLSGIARGCGWQHLGAYVNLGAYYVVGIPIAAMLGFWVQLRGKGLWIGILTGAFCQTVMLSLITSCTNWEKQAIKA

	RERTFQRSFAVEDGLVLANGENSLNLSACLVGIGKSSNFGGTKDQNLELEEGKVLITRQV*
Glyma.20G123400.1.Wm82.a2.v1	MENSLLDKDSEPENPSASAITWTVFSQEMKRVGYLAAPMITVTLSQYFLQIISMMMVGHLGKLALSSTAIAISLCAVSGFSLIFGMSCALETQCGQAYGAQQYRKFGVQIYTAIVSLTLACLPLTLFWVYLEKILIFLGQDPSISQEAGKFALCMIPALFAYATLQALIRFFLMQSLISPLVISSSITLCFHVAFSWLMVFKSGFGNLGAAFSIGTSYWLNVILLGLYMKFSTECERTRVPISMELFHGIGEFFTYAIPSAGMVCLEWWSFELLTLLSGLLPNPELETSVLSICLSIITTIYTIPEAIGSAASTRVSNALGAGSPQSARVSVSAAMTLAVSEAILVSSIIFASRQVLGYVFSNEQDVVDYVTDMVPLLSISVIVDTLHGTLSGIARGCGWQHIGAYVNL*
Glyma.20G123500.1.Wm82.a2.v1	MEESLVKKHEEDRVVRWGVYSEEMRRICEIAGPMVAVVSSQYLLQVVSTMIVGHLGELYLSSAALAISLSGVTGFSLHMGMASGLETICGQAYGAQQYQRIGMQTYTAIFSLILVSIPVSILWINMESILVFIGQDPLISHEAGKFTIWLVPALFAYAILQPLVRYFQVQSLLLPMFASSCVTLIIHVPLCWALVFKTRLSNVGGALAVSISIWSNVIFLGLYMRYSSACAKTRAPISMELFKGMWEFFRFAIPSAVMVCLEWWSYELLVLLSGLLPNPQLETSVLSVCLNTISTLYMIPFGIGAAASTRVSNELGAGNSHAARVAVLAAMSLAVIETSIVSATLFACRNVYGYIFSNEKEVIDYVTVMAPLVCISIILDSIQGVLTGIARGCGWQHLGVFVNLGAFYLCGIPMAALLAFLVRLGGQGLWIGIQSGAFVQTLLLSIITGCINWEKQAIKARKRLFDDQFSADNILV*
Glyma.20G155500.1.Wm82.a2.v1	MCHITSHLPCKCNSNPEYVVSMKPPEADMMITNPLIQKDITVTLTTLHEEKNDPRTMQTHHHVLKELISICKIAFPMILTGLLLYCRSMISMLFLGRLGELALAGGSLAVGFANISGYSILSGLAVGMESICGQAYGAKKFSLLGLCLQRTILLLLFTCIPISLLWLYMKHILLLCGQDEAIATQAQSYLLYSIPDLLAQSFLHPLRIYLRSQSITLPLTLCATFSILLHIPINYLLVSHLNWGIKGVALSGVWTNLNLVASLILYIVFSGTHKKTWGGFSFECFTQWKSLLNLAIPSCISVCLEWWWYEIMILLCGLLVNPRATVASMGILIQTTSLLYIFPSSISFSVSTRVGNKLGAQKPSKAKFSSIVGLSCSFMLGVFALVFTILVRNIWANMFTQDKEIITLTSFVLPVIGLCELGNCPQTTGCGVLRGTARPKVGANINLGCFYLVGMPVAVWLGFFAGFDFQGLWLGLLAAQGSCAVTMLVVLSRTDWDAEALRAKKLTSVVVDDSKEVGAEKPPKAEIKEDSLLSLADSDEDKQSWVSTFW*
Glyma.20G162000.1.Wm82.a2.v1	MDAPLLLVNGEGAALVAENGDYVAVRELKEVKKVFWIETKRVWEIAMPIVFNIWCQFGVNSVTSMFVGHLGDIQLSAISLINSVIGTFAFGFMLGMGSATETLCGQAFGAGQVNMLGVYMQRSWVILSVTSILLLPIYIFAAPILKLLGQQEDIADLAGSFSILVIPQFLSLPFNFPTQKFLQAQSKVKVIAWIGLVALILHIGMLWFLIYVLDFGLAGAALAFDITSWGITVAQLVYVVIWCKDGWNGLSWLAFKDIWAFVRLSLASAVMLCLEVWYMMSVIVLAGHLDNAVIAVDSLSICMNINGWEAMLFIGVNAAVSVRVSNELGLGHPRAAKYSVYVIVFQSLFLGIFFMAIILATRDYYAIIFTNSEVLHKAVAKLGYLLSVTMVLNSVQPVVSGVAIGGGWQALVAYINIGCYYLFGLPLGFVLGYTANLGVEGLWGGMICGIVLQTLLLLLILYKTNWKKEVEQTAERMRIWSGQDIGVDKIVASA*
OsMATE1	MGSSDSQAPLLLPRGSHRKEEEEEEYAAAGKVRGCCGGDGEGGWWREATAEAGRLASLAAPMIAVALLQLMMQLISTVMVGHLGEVALAGAAIANSLTNVSGFSVLMGLACGLETICGQAYGAEQYHKLALYMYRSIIVLLVVSVPIAIIWVFIPEVLPLIGQQPEIASEVGKYALWLIPGLFAFTVAQCLSKFLQTQSLIFPMVLSSSITLALFIPL

	CWFMVYKVGMGNAGAALSVSICDWVEVTVLGLYIVLSPSCEKTRAPLTWEAFSGIGSFLRLAVPSALMICLEWWSYELLVLLSGILPNPALETSVLSICISTVVLVYNLPHGIGTAASVRVSNELGAGNPEGARLVVGVALSVILCSAVLVSVTLLALRHFIGIAFSNEEEVINYVTRMVPVLSISVITDSLQGVLSGVSRGCGWQHLGAYVNLGAFYLVGVPVALFFGFAMHLGGMGFWMGMVAGGATQVTLLSIITAMTNWRKMAEKARDRVFEERIPTQSV
OsMATE10	VRVSQVAGEAQAIGRVSVPMAVTGLVMYSRALISMLFLGRLGELALAGGSLALGFANITGYSVLSGLALGMEPICGQAFGARRGKLLALALHRTVLLLLAVALPISLLWVTSTGYILKQLGQDEGVADAAQTFAAYASADLAVLAVLHPLRVYLRSQNLTLPITACSLFSVLLHGPINYLLVVRLRMGVAGVALAVALTDLNLLLALLCFLAISGAHRDSWVGPTSDCLRGWPALLRLAVPTATAVCLEWWWYELMIVLSGLLANPRATVASMGILIQATSLVYVFPSSLGQGASTRVSHQLGAGRPAGARRAAGAALSIGLVVGAAAATFMVSVRSHWGRMFTSDGEILRLTAVALPIAGLCELGNCPQTAGCGVLRGSARPASGARINLASFYLVGMPVGVALAFGARLGFAGLWLGLLAAQAACAVWMARAVAATDWDVEVARAKELTKASTTGSGTNHQHECNNSNTNTANAKANTKTTTSPAANNINAGGGGSSDNRGYVPISESGHNDGSDDLEKLEEGLMVATSGGCCGCGDALGVDTKAGDKQQCSNGGAGTAEGNAGQRRGSASSERAPLISVGDDEEAGEENDGDGGGGGHV
OsMATE11	MAGGDQRGDSPSSHELSGRLEGILADGEAPWARRACKAAALEVRLLAPIAAPAIVVYVPNNVLSISTQIFCGHLGNLELAASSLGNNGIQIFAYGLMLGMGSAVETLCGQAYGVHKYDMLGVYMQRSTVLLMATGVPLAVIYAFSRPILVLLGESPEIASAAAVFVYGLVPQIFAYAANFPIQKFLQAQSIVAPSAYTSAATLVLHLVVGWLVVYQLGMGLLGASLVLSLSWWVIVAAQFVYIAASKRCRRTWTGFSWMAFSGLPEFLKLSTASAVMLCLETWYFQILILLAGLLDDPQLALDSLTVCMTLAGWVMMISIGFNAAASVRVGNELGAGHPRAAAFSVVVVTAVSFVITVVMAVVFLMFRDYISYIFTEGETVARAVSDLCPFLAATLILNGIQPVLSGVAVGCGWQKIVAYINVGCYYFVGIPLGFLLGFKFHLGAKGIWTGMLGGTCMQTLILFWITFRTDWNKEVEEAKKRLNQWEDKKQPLLAGTVDY
OsMATE12	MGTESDQYPAHLSSSATVAGHGDDGESPEIAGAARLYVVGLIPQIFAYAANFPIQKFLQAQSIVAPSAYISAATLAAHVALSWFAVYKLGLGLLGASLILSLSWWVIVLAQFAYIVVSDRCRLTWAGFSSKAFSGLPEFLQLSAASAVMLCLETWYFQVTVLIAGLLKDPEIALDSLAVCMSISGWVFMVSVGFNAAASVRVSNELGAGNPRAAAFSVKVVTSLSLIVAAIIAAIVMCLREYLSYVFTQGEEVARAVSSMTPLLAVTIVLNGIQPVLSGVAVGCGWQAFVAYVNIGCYYIIGVPFGCVLGFHFDLGAMGIYGGMIVGLFVQTLILVYVTFRTDWNREVGEAKKRLNKWGDIAKPLLANED
OsMATE13	MGSSVKDAGGGKEEQQQLESPLLEAAVSSGGGDGGGGHGVSGELESILGDETVPWARRMWAATGVEMRLMLRLAAPAVLVYMINYLMSMSTQIFSGHLGTLELAAASLGNTGIQVFAYGLMLGMGSAVETLCGQAYGAHKYDMLGVYLQRSTVLLMATGVPLAVIYAFSRPILVLLGESPEIASAAAVFVYGLVPQIFAYAANFPIQKFMQAQSIMAPSAYISAATLAFHLVLSYLVVYQFGLGLLGASLMLSISWWVIVVAQFIYIVTSRRCRLTWTGFSMLAFSGLPDFFKLSLASAVMLCLETWYFQILVLIAGLLKDPEMALASLSVCMTISGWVFMISVGFNAAASVRVSNELGAGNPKSAAFSVVVVTVLSFFLSVVISLVILLCRDYISYIFTDGEDVATAVSKLTPLLALTLILNGIQPVLSGVAVGCGWQAFVAYVNVGCYYIVGIPLGCLLGFYFDLGAAGIWSGMIGGTLM

	QTLILMWVTFRTNWNREVEEAMKRLNKWEDKTPLLSE
OsMATE14	MAGGVSDGGAAHGASGRLESILSDSSMPLARRAWAATTIELGLLTRIAAPAVV<br>MYMINYLMSMSTQIFSGHLGNLELAAASLGNNGIQMFAYGLMLGMGSAVETL<br>CGQAFGAHKYDMLGVYLQRSAVLLTITGVPLAVIYGFSEPILVFMGQSPEIARA<br>AAIFVYGLIPQIFAYAINFPIQKYMQAQSIVLPSAYISAATLALHVLLSWVVVYKV<br>GLGLLGASLVLSISWWVIVAAQFAYIVTSPTCRHTWTGFTWQAFAGLWDFLKL<br>SAASAVMLCLESWYFQVLVLIAGLLPNPELALDALSVCMTISGWVFMISVGFNA<br>AASVRVSNELGAGNPKAAYFSVWVVTISCAIISAILAVVILCLRNYISYLFTEGEV<br>VSNAVADLCPLLAITLILNGIQPVLSGVAVGCGWQQFVAYVNIGCYYIVGVPLG<br>VLLGFVFKLGVKGIWGGMLGGTCMQTAILVWVTLRTDWNNEVEEAQKRLNK<br>WEDKKKEPLLTGIRDNN
OsMATE15	MGKLVSKSWQESKLLWHIAFPAILTAVFQFSIGFVTVGFVGHIGQVELAAVTVV<br>ENVIEGFAYGVLLGMGSALETLCGQAVGAGQVSMLGVYIQRSWIICGATAVILT<br>PTYVFTAGILIGLRQPTDIAAVAGTYTRWVIPQLFAYAANFPLQKFFQSQSKVW<br>AMTAISGIALALHVVLNYIFLTRLGHGLVAAALIGNVTWWLIILAQFIYLVSGCF<br>PEAWKGFSMLAFKNLAAFVKLSLASAIMLCLELWYYTAVLILVGLLKDAKLQV<br>DVMSVCINYQLWTLMVALGFNAAVSVRVSNELGANRPKAAKFAVAMAVSTS<br>AIVGAVFMAVFFIWRTQLPRFFSDDADVVRESAKLGYLLAATIFLNSIQPVLSGV<br>AIGAGWQSLVAFINIGCYYLVGIPLGVLFGFKLKLDAMGIWVGMSLGTLLQTAI<br>LAFISFRTKWERQAMMAEERIREWGGRNDDALPSTTTPTADDHNVDR
OsMATE16	MAIPLQGKAQQQQGEGGKGDGAVDDDGDDQPSVASELRELWGMAAPITAL<br>NCVVYLRAMVSVLCLGRLGPLDLAGGALAIGLTNITGHSVLFGLASGLEPLCA<br>QAFGSKNYDLLTLSLQRAVLLLTLAALPIALLWLHVGPILVALGQDPTISASAA<br>AYAAYALPDLAASAVLQPLRVYLRSQGITKPMAACSAIAVALHVPLNVLLVFG<br>LGFGVRGVAAAQALTNTNMVLFLLAYIRWSRACDATWKGWARPAAVASGLA<br>GLVRLAVPSCVGVCLEWWWYEVVTVLAGYLPDPAAAVGAAGVLIQTTSLMYT<br>VPMALAACVSTRVGNELGGGKPRRARMAAMVALGCAVVIGVVHVAWTAAF<br>SREWVELFTREAAVVRLAAAAMPILGLCELGNCPQTTGCGVLRGTARPAVGA<br>RINLLSFYLVGTPVAVTLAFGARVGFGGLWYGLLSAQAACVALVLLAVVWRT<br>DWHLEALRAKKLTGLEMIAAAAEGDDDECKRLIAPLPPPDGHDVAVDVV
OsMATE17	MTPPPPSPPHERKTWAESVASEFRAQRGIAFPLIAMNLTWFAKLAVTTAFLGRL<br>GDLQLAAGTLGFSFANVTGFAVLTGLCAAMDPICGQAHGASNGKLLRKTLVM<br>ATILLLGASIPIAFLWLHVDAVLLRFGQQADMSSNARSYVVCLLPDLAVTSFVN<br>PLKSYLSAQGVTLPTLFASALALALHVPLTMWMARTRGIQGVATAVWVSDLA<br>VAVMLAGYVLVSERRRKAGGGGGWVEQTRGEWVRLLRLAVPSCLNTCLEWW<br>CYEILVLLTGRLPDARRTVAVMAVTLNFDYLLFAGMLSLSVSASVRVSNELGAG<br>EAWAARRAGMVSIVGGAVGGVGGGVAMVAARRAWGSIYSSDAGVREGVGR<br>AMEVMAVLEVVNFPLNVCGGIVRGTARPAVGMYAVVAGFYVLALPLGVALA<br>FKARLGIQGLLLGFLVGAAASLAVLLTFIARMDWPAEAQKARTRTTATVAQFH<br>QHDEVVQP
OsMATE18	MAAPPSMEEPLLGGGNGEEKKGGSSRLAVVAEVRKQLYLAGPLIAGWLLQNV<br>VQMISVMFVGHLGELELSSASIATSFAGVTGFSLLAGMASSLDTLCGQAFGAKQ<br>HRLVGVYKQRAMVVLGLASVCVAAVWAYTGELLLLFGQDPEIAAAAGSYIRW<br>MIPALLAYGPLQCHVRFLQTQNAVMPVMLSSGAAAACHLPVCWLLVYGAGL

	GSKGAALANAVAYLANAAALAAYVRLSPACRSTWTGFSSEAFHDLVGFMRLA<br>VPSALMVCLEWWSFELLVLLSGLLPNPKLEASVLSICLNSGSLAFMIPFGLGSAIS<br>TRVSNELGAGRPEAARLASRVVMALGLVVGVAIGLAMILVRHLWGYAYSNEE<br>EVVQYVAKMMPILAVSFLFDDLQCVLSGVARGCGWQKIGAIVNLGAYYLVGIP<br>AALCFAFVYHLGGMGLWLGIMCALIVQMLLLLAITVCTNWEKEALKAKERVF<br>SSSLPADMT
OsMATE19	MTTCADDQTGCAFFAPLLSSKGAEVVILVAGDEAEEQQPAPVLTSKPPGRLAK<br>AVNEAWSVSLGVAFPVTPSMFTCSARGEARSILGLAFPMILTGLLLYLRSMISML<br>FLGHLGGLALAGGSLAIGFANITGYSVLSGLAMGMEPICGQAFGAGNYALLGV<br>TMQRTVLLLIAAAIPIGGLWVQMRPLLLFCGQDAAIAAVAETYIFASLPDLVLQ<br>AFLHPVRIYLRTQSINLPLTVCAGLAIAIHLPINYVLVVVLGLGVKAVALASVLA<br>NLNLVLFLLAYIFLKGVHKRTGGFLLSAESFRGWGELISLALPSCVSVCLEWWW<br>YEIMILLCGLLLNPQATVASMGILIQTTSLIYIFPSSLSFGVSTRVSNELGAGQPEE<br>ASRAATVGLVLGFGFGAFASAFAFLVRNVWASMFTADPAIVALTASVLPILGLC<br>ELGNCPQTTGCGVLRGSARPKDAASINLRSFYLVGTPVALVMAFWFHLDFRGL<br>WFGLLAAQATCTVRMLLVIGRTDWAAEAKRSKQLTGAGAANMESDDRVAA<br>DEKSRLPVDTDVERSSDHTDRC
OsMATE2	MAAAAREEQPLLLRREEGEEEGEEVGWRRRWGSEAGKLAYLALPMVAVSLTN<br>YAVQVFSNMMVGHLPGVLPLSSAAIATSLASVTGFSLLIGMASALETLCGQAYG<br>AKQYHTLGVHTYRAILTLLVVCIPLSLLWVFMGKILVLIGQDPLISHGAGRYIVW<br>LIPGLFANALIQPITKFLQSQSLIMPMLVASVATLVFHIPLCWLMVFKTGLGYTG<br>AALSISISYWLNVAMLVAYILLSSSCKETRTPPTIEAFKGLDGFLRLALPSALMICL<br>EWWSFELLILMSGLLPNPELQTSVLSICLTSITLLFTIPYGLGAGGSTRVANELGA<br>GNPEGARSAVYVVLSVAVTEALIVCGTLLASRRLLGRAYSSEEEVISFVAMMVPL<br>VCITVVTDGLQGVMSGIARGCGWQHLGAYVNLGSFYLLGIPMAILLGFVLHM<br>GAKGLWMGIVCGSISQITLLSAITFFTNWQKMAENARERVFSEKPTEPSRYHLV<br>E
OsMATE20	MEERIPLLSKRFPADGTAGVGGGREEEGGDRWWSGLAREAGKVGSMALPMA<br>AMSVAQNAVQVASNMMVGHLPGVLPLSASAIATSLASVSGFSLLVGMASGLE<br>TLCGQAYGAKQYDKLGVQTYRAIVTLTVVTIPISLLWVFIGKLLTLIGQDPVISHE<br>AGRYIVWLIPGLFAYAVCQPLTKFLQSQSLIFPMLWSSIATLLLHIPLSWLLVFKT<br>SMGFTGAALAISISYWLNTFMLAAYIRFSCSCKVTRSPPTIEAFRGVGLFLRIALPS<br>ALMLCFEWWSFEILVLLSGLLPNPELESSVLSICLTTTSLMYTIPYGLGGAASTRV<br>ANELGAGNPEGARSAVHLVMSIAGTEAVLVTGMLFAAQRILGYAYSSDEEVVT<br>YFTSMVPFVCISVAADSLQGVLSGIARGCGWQHLGAYVNLGSFYLVGIPVALLL<br>GFGFKMEGKGLWLGIACGSVLQFLLLAVIAFFSNWQKMAEKARERIFGETPSEK<br>QHLVLDATNSV
OsMATE21	MAAKGSPEEEALLAGVGGDHQLVESDELAPAAAVVREEVKKQLWLAVPLVA<br>GALLQNVIQMISVMFVGHLGELPLAGASMASSFASVTGLSLLLGMASALDTLC<br>GQAFGSRQYHLLGVYKQRAMLLLTAVSVPLVVVWFYTGDILVAFGQDADIAA<br>EAGAYARWMIPALFAYGPLQCHVRFLQTQNVVLPVMASAGAAALCHLVVCW<br>ALVYAAGMGSKGAALSNAVSYWINVAILAVYVRVSSSCKKTWTGFSMEAFHD<br>PLSFFRLAIPSALMVCLEMWSFELIVLLSGILPNPKLETSVLSISLNTAAFVWMIPF<br>GLGSAISTRVSNELGAGRPRAARLAVRVVVFMAVSEGLVIGLVLVGVRYIWGH

	AYSDEEEVVTYVAKMMLVIAVSNFFDGIQCVLSGVARGCGWQKIGACVNLGA<br>YYIVGIPSAYLIAFVLHVGGMGLWLGIICGLLVQVLLLMAITLCTNWDKEAANA<br>KDRVFSSSLPSDLAT
OsMATE22	MEKASCLEEALLLPESCKEEEITASDEVKRQLRLAGPLIAGSLLQNLIQMISVMF<br>VGHLGELPLAGASMASSFAAVTGFSLLLGLASALDTLCGQAFGARQYHLLGVY<br>KQRAMLLLTAVSVPLAVAWYYTGDILLLFGQDADIAAEAGAYARWMIPALFA<br>YGPLQCHVRFLQTQNMVVPVMAAAGAAALCHLGVCWALVHAAGMGSRGA<br>ALGNAVSYWINVGVLAVYVRVSRSCKKTWTGFSMEAFRDPLSFFRLAIPSALM<br>VCLEWWSFELLVLLSGLLPNPKLETSVLSITLNTANCLFMIPFGLGAAISTRVSNE<br>LGAGRPRAARLAVRVVTLLATLEGLGMALVLACVRYVWGHAYSNEEEVVAY<br>VAKMMLVLAVSNFLDGIQCVLSGVARGCGWQKIGACINLGAFYVVGVPAAYL<br>AAFVLRAGGLGLWMGIICGVAVQTLLFLAITSRTDWQKEAKMAKDRVFSSSLP<br>TDLAT
OsMATE23	MCTTTSAPSVPEVATPADGGGHHVVYVSLPQCTDGGDVEGGHCRPVVHQVKC<br>RGGDDDGGGGGRGGGVVMPAAGETVREAAALCRLACPIALTALMLYSRSALS<br>MLFLGSLGDLPLAAGSLAVAFANITGYSVLSGLSLGMDPLCSQAFGARQPRLLG<br>LTLYRSVLFLLCCSLPLSALWLNMAKILLFLGQDRDITAMAQDYLLFSLPDLFSFS<br>LIHPLRVYLRSQGITQPLAVAAAAAVVFHVPANYVLVGRLRLGAPGVAAAASA<br>SNFVLLAVLLAYVARRDEALREAGGPTAEWLAGWGPLARLAAPSCVSVCLEW<br>WWYEVMILLCGLLPEPRPAVASMGVLMQTTALVYVFPSSLGFGVSTRVGNELG<br>ANRPGRARAAAHVAVAGAAAMGLAAMAFATGMRHAWGRLFTADADILRL<br>TAAALPVVGLCELGNCPQTVGCGVLRGTARPARAAHVNLGAFYLVGMPVAV<br>VLAFGLGVGFVGLWVGLLAAQVCCAGLMLCVVGSTDWEAQARRAQALTSSA<br>AVSGKADAAEGGGRWPEKGEHQEWEKRRHVALISSEEADPETAEVL
OsMATE24	MSGGGGEVEAAAEAAPLLVPHDPQPAVGAEVRRQVGLAAPLVACSLLQYSLQ<br>VVSVMFAGHLGELSLSGASVASSFANVTGFSVLLGMGSALDTFCGQSYGAKQY<br>DMLGTHAQRAIFVLMLMGVPLAFVLAFAGQILIALGQNPEISSEAGLYAVWLIP<br>GLFAYGLLQCLTKFLQTQNIVHPLVVCSGATLVIHILLCWVMVHCFDLGNRGA<br>ALSISLSYWFNVILLAIYVKVSEVGRRSWPGWSREALKLKDVNMYLRLAIPSTFM<br>TCLEYWAFEMVVLLAGFLPNPKLETSILSISLNTMWMVYTIPSGLSSAISIRVSNE<br>LGARNPQAARLSVFVSGIMCLTEGILVAIITVLVRDIWGYLYSNEEEVVKYVAA<br>MMPILALSDFMDGIQCTLSGAARGCGWQKVCSVINLCAYYTIGIPSAVTFAFVL<br>KIDGKGLWLGIICAMTVQILALVVMLLRTSWNEEAEKARARVQGSDGRITLA
OsMATE25	MGGDERAVAAPLLQQQQDGGGGDGERRRRRWWWGWWDGEEAAGQLAFA<br>APMVATSMAYYAIPLVSVMYAGRLGELELAGATLGNSWGTVTGIALMTGLSGS<br>LETLCGQGYGAKMYHMMGVYLQASIITSAFFSVLVSLLWFYSEPVLIFLRQDPEV<br>TRTATLFLRYSIPAQFAYGFIQCTLRFLQTQSVVTPLVVFALLPLVLHFGITHAFV<br>HYLGFGYAGAGMSTSVSLWLSFLMLAAYVCLSERFKHTWEGFSTEAFRHVLPG<br>LKLAIPSAVMVCFEYWAFEVLVLVAGLMPNSHMSTSIIAMCENTEAISYMITYG<br>FAAAISTRVSNELGAGNVAKAKKALAVTLVLSLLLGVAFLLLLGLGHDLWAGL<br>FSKSDAVISEFASMTPLLIGSVVLDSTQGVLSGVSRGCGWQHLAAWTNLVAFYI<br>VGLPLSILFGFKLGLQTKGLWLGQICGLLLQNAVLLFITLRTKWERLELTMNGK<br>EDGFVC
OsMA	MASTDPLLGGKEEEEGGGEVRRARRWWVGRVVDTEEAWAQTRFAVPMVLTN

TE26	MSYYAIPLVSVMFSGHLGDVHLAGATLGNSWATVTGYAFVTGMSGALETLCG<br>QAYGARMYRMLGLYLQSSLLMSAAVSVLVSALWCFTEPLLLLLRQDPAVSAAA<br>SAFVRAQVPGLFAFSFLQCLLRYLQTQSVVAPLVACSLAPFLLHVALAHLLVNA<br>LGLGLAGAGAAVSITFWASCLMLLAYVLRSERFAETWNGFSAEAFRFVVPTIKL<br>ATPSAVMVCLEYWAFELLVLIAGLLPNPTVSTSLIAMCSSTEAIAYMITYGFSAA<br>VSTRVSNEIGAGNVEGAKNAVAVTLKLSVFLAAAFVLLLGFGHGLWAGLFSGS<br>AIIAAEFAAVAPLMMASILLDSAQGVLSGVARGCGWQHLAAVTNLVAFYVIG<br>MPLSIFFAFKLKWYTKGLWMGLICGLTCQTCTLMVITARTKWSKIVDAMQEKK<br>ASYVA
OsMATE27	MGHAVDGRLEALLSGGGGGGEAAAPWARRMAAAAALELRLLAPLAAPAVV<br>VYMLIIVMSSATQIFCGQLGNVQLAASSLGNNGIQVFAYGLMLGMGSAVETLC<br>GQAYGAGRHEMLGVYLQRSAVLLTAAGVPLAALYACSERVLLLLGQSPEISRA<br>AAGFARGLIPQIFAYAANFPIQKFLQAQSIVAPSAAVLAASFALHLPLSWAAVR<br>VLGLGLPGAALALSATWWVLVAGQFAYIVRSPRCAATWTGFTWAAFHDLAA<br>FARLSAASAVMLALEVWYFQVLILLAGMLPDPQIALDALTVCTSIQSWVFMISV<br>GFNAAASVRVGNELGAGNPRSAAFSTWMVTALSAIIAAIAGVVVILLRDKLSYI<br>FTQGEAVSRAVSDLCPLLVGTIVLCGIQPVLSGVAVGCGWQALVAYINIGCYYLI<br>GLPLGVLLGFKFDYGIKGLWGGMIGGTLIQTLILIWITFRTDWNKEVEDARRRL<br>DKWDDTKQPLLVNRQ
OsMATE28	MEDEATSVAAPLLRPRGGVDAEAVKQQLWPAGARVAGEWWVESKKLWRVV<br>GPAIFQRIALYGINVVSQAFIGHMGDLELAAFSIASTVVAGFNFGFLLGMASALE<br>TLCGQAFGAKKYHMLGVYLQRSWLVLLMFAVALTPTYVLMEDLLLLIGQPAD<br>LASLAGKMSVWLLPQHFAMAMLLPLTRFLQSQLKNWVTAVTAGVALALHLVI<br>TYLLVNTLHLGLLGAVAAANVAWWIVVLGQLVYVVGGWCPLSWKGFSMEAF<br>ADFWEFIKLSSASGVMLCLENWYYRVLVLLTGYLNNAEIAVDALSICLTINGWE<br>MMIPFGFLAATGVRVANELGAGSGKGARFAIVVSVTTSVAIGLVFWCLIIAYND<br>KIALLFSSSKVVLDAVSDLSVLLAFTVLLNSVQPVLSGVAIGSGWQALVAYVNV<br>GSYYLVGVPIGAILGWPLHFGVGGIWSGLIGGTAVQTLILAYLTISCDWDEEAK<br>KASTRMEVWASSK
OsMATE29	MCHCSSKVVAVAQCHQPLLPPPEACPALHDRPRSARGGGGAIAEVASIVRLA<br>MPMVGAGLLMYMRSLVSMLFLGRLGRLPLAGGSLALGFANITGYSVLSGLAAG<br>MDPVCGQAFGAGRTSVLAAALRRTVVLLLAASVPIAALWLAMHRVLVAAGQ<br>DPDIAACAYEFILCSLPDLAVQSFLHPLRVYLRAQSITLPLTYAAAAALALHVPV<br>NVLLVHGLGLGIRGVALAAVWTNLNFLLFLVAYAYFSGLIRGDDDDDGGNGK<br>AGEEGATTTTTTMEWGWLVKLSVHSCMSVCLEWWWYEIMVLLCGVLADPKA<br>AVAAMGILIQTTSLLYIFPHSLSCAVSTRVGHELGAGRPERARLAARVGLACGA<br>ALGVVACAFAASLRGVWARMFTADATILRLASSALPILGAAELGNCPQTVGCG<br>VLRGSARPGRAARINVSAFYGVGMPAALALAFWPARLDFRGMWAGMLAAQL<br>VCAALMLLAVRRTDWDEQAARAREITGAVAGVVVGDGDVVVKGDHADAAK<br>VKADSGLLVVTVLS
OsMATE3	MLIDPPCNRIHSARTQLQRCRTTTTGGLSALRYTVRGLSLLYMCTASSCAHTHT<br>HNRTEGGARTGLRSEMAPPAGTEAAGAGHRKNWRGESGNLWRIAGPVILTEI<br>FQFLIGFVTAAFVGHIGKVELAAVSVVNGVVEGLAFGLLLGMGSALETLCGQA<br>VGAGQPRMLGVYLQRSWVICLATSLALLPLYLLASPALRLLRQSAAISSVAGRY

	ARWCAPQLFAYAVNFPMQKFYQAQSRVWAVTAISAAALAAHALLNWLVVA<br>RLGHGVVGAALVGDVSWWLLNAAQFAYLVGGSFPEAWSGFSRKAFTSLGGFV<br>KLSLSSAVMLCLEMWYYTAVLILVGCLKNPEIQVGAISICMNYQLWTLMVAVG<br>FNAAVSVRVANELGANHPKAAKFSVIVAVVTSAAVGLVFTLVALVARKQLPRL<br>FTDDDVLVRETAKLGYLLAATIFLNSIQPVLSGVAIGAGWQSSVAFVNIGCYYL<br>VGLPIAAVFGFRLSLNATGIWVGMLIGTILQTVILLVILYRTKWQKEAMLAEERI<br>KVWGGGVELP
OsMATE30	MSSTSGSAWDHSNNGGGGSPELREALLLGDGGSSPESREIKGIAVKKQDDLEEI<br>RSVGELMRLAAEENRRLWYLAGPAIFTSLAQYSLGAVTQVFAGHLTTLELDAV<br>STENMVIAGLAFGIMYGMGSALETLCGQAFGAKQHHMLGIYLQRSWVILTAM<br>SVILLPIYLFATPILRFFHQDDEIAVLASRFSLYMIPQLFAYALNFPIQKFLQAQSK<br>VMAMAAVSAAVLLFHVALTWLLLVPLRMGLVGLAVALNVSWWLVVLGQLA<br>YIVMGYCPGAWNGFDWLAFTDLLSFARLSLGSAIMICLEFWFYMFLIVIVGNLP<br>NAQVAVAAVSICTNLFGWQIMVFFGFNAAISVRVSNELGAGRPRAARLAIAVV<br>LVSSVAIGVAFFAAVLLLRDVYGAPFTGSPEVVRAVASLGVVFAFSLLLNSVQPV<br>LSGVAVGAGWQWLVAYINLGCYYCVGIPVGYAIAFPLRRGVQGMWGGMLTG<br>VGLQTAILVAITARTNWNKEASEAHARIQHWGGTAKLAVDDPI
OsMATE31	MGSVSPPAPEEDAAAVESAGAAARMFWHETKRLWAIGTPIAIGTITNYAISSVT<br>TMFIGHLGNLPLAAASVGLSVFATFALGFLLGMGSALETLCGQAFGAGQVSML<br>GVYLQRSWIILLGATVLMVPVYVLAEPLLLLVGQDPEVARAAGRFTLYILPGAF<br>AFAVNFPSGKFLQAQSKVGVLAWIGVAGLAFHVGITYLAVSVLGWGLPGAAA<br>AYDVSQWASSLAQVAYIMGWCREGWRGWSMAAFHDLAAFLRLSIESAVMLC<br>LEIWYLGLITVLTGDLDDAQMAVDSLGICMNINGYEGMIFIGLNAAISVRVSNE<br>LGSGRPRAAMHAVVVVVAESLLIGLLCMALVLAFSDKLALVYTSDAHLLRAVS<br>RIAGLLGVTMVLNSVQPVLSGVAVGGGWQGLVAYINLACYYLFGLPVGYLLG<br>YYFNLGVGVPIPIPIPTYLPSLFFFPLI
OsMATE32	MASVPLLAEWPAGKEKEEGRVRRRLPALAREAWEESKKLWEIVGPAVFLRLVL<br>YSFNIISQAFAGHIGDLELAAFSIANNVITGLNFGFLLGMASALETLCGQAYGAK<br>QCSMLGIYLQRSWIILFVFAVLLVPTYVFTAPLLEALGQPAALARKAGMVSVYM<br>LPSHFQYAVLLPLNKFLQSQRKNWVTVVTAAAAFPVHIAVSWLLVSRLRFGVL<br>GAAMSLGVSGWLVTLLQLAYVVGGGCPVTWSGFSPLAFVDLWGFVKLSVSSG<br>VMVCLETWYYKILILLTGHLKNSELAVNALSICMSFQSWEMMIPVGFLAGTGV<br>RVANELGAGNGKGAKFATIVSTTTSFLIGLFFSALALAFHDKIALVFSSSNAVID<br>AVDNISFLLAVTILLNGVQPVLSGVAIGSGWQAAVAYVNIGCYYFIGVPIGVLL<br>GWSFNLGVFGIWAGMIAGTAIQTIILAHMTIQCDWNKEVLQASERVQRWGNP<br>K
OsMATE33	MASRHSDEATQCHQQLLVMPAATASYPKLHDRPRLAGAAAGVLGEVASILCL<br>AGPMVGAGILLYLRSLVSMVFLGRLGQLPLAGGSLALGFANITGYSVLSGLAGG<br>MDPVCGQAFGAGRTDLLRAALRRTVVLLLAASVPISALWVAMHRVLVATGQ<br>DPDIAATAYAYILCSLPDLAVQCFLHPIRIYLRAQSVTLPLTYAAAAALLLHVPI<br>NVVLVDRLGLGIRGVALGAVCTNLNCLLFLAAYVCLSGMYGGRAKACASAAA<br>PAAGEEDDDGGVREWWSLVRLSVHSCMSVCLEWWWYEIMVLLCGVLADPKA<br>AVAAMGVLIQTTSLIYIFPHSLGCAVSTRVGHELGAGRPERARLVARVGVGLGA<br>ALGIVAFGFAVSVRAAWARMFTAEDAILRLAAAALPLLGAAELGNCPQTAGC

	GVLRGSARPERAARINVAAFYGVGMPVALALAFWPAGLDFRGMWGGMLAA<br>QLVCAWLMLRAVLGTDWAEQAERARELTGGGDGYAAVAAVIVDDDKAKQH<br>AEMDKPQQVDNTLLMAIDCV
OsMATE34	MELAGGGTGVVRQRAEPLGAGLLLRGGGRSVGGGGIGCARRATLRGLALSPL<br>ARRAVSAAGGHFLPRRAVRAAAAAGDGGFYGEEDAASDQPFPARASPSDDA<br>NDSTAVRSLGGDHPGEIKKELLNLALPAIVGQAIDPVAQLLETAYIGRLGPVEL<br>ASAAVGVSVFNIISKLFNIPLLSITTSFVAEDVARHDSDQFTSEGNMSSESGGRKR<br>LPSISSAILLAAAIGVIEASALILGSEILLSIMGVSHASTMHSPAKLFLSLRALGAPA<br>VVVSLAIQGIFRGLKDTKTPLLYSGLGNISAVLLLPFLVYSLNLGLNGAALATIAS<br>QYLGMFLLLWSLSKRAVLLPPKIEDLDFVGYIKSGGMLLGRTLSVLITMTLGTA<br>MAARQGTIAMAAHQICLQVWLAVSLLSDALAVSAQALIASSFAKLDYEKVKE<br>VTYYVLKIGLLVGAALALLLFASFGRIAELFSKDPMVLQIVGSGVLFVSASQPIN<br>ALAFIFDGLHFGVSDFSYSASSMITVGAISSLFLLYAPKVFGLPGVWAGLALFMG<br>LRMTAGFLRLGSRAGPWWFLHQKEPTYKLHSSTC
OsMATE35	QKLIRLFSSISLQEACGNQCELLRRCSDLHCHHEGGGDGGAAHRRQQWRRGG<br>EERRRGGGVGGDRDQEAAVPRRAARRRHVAAERRPDDFRHVRRPPRRARPLQ<br>RLHGHLLRRRHRLQLAGWHGEQPGHAVRPGVRREAAPHARRVQAAGDAGA<br>RAGERPDRGGLGLHRRDPPRRRPGPGDRGGRRELHPVDDPDAVRLRAAAVPR<br>PVPADAERRRAGDALRRRDGGEPRARLLAARAQARAGRQGRRARQRRLLPH<br>QPLRAGHLRLATRVVMLLAFLVGTSEGLVMVIVRNLWGYAYSNEEEVADYIAK<br>MMPILAVSILFDAIQCVLSGVVRGCGRQQIGAFINLGAYYLAGIPVAFFFAFVCH<br>LGGMGLWFGILCGLVVQMLLLLTITLCTNWDKEALKAKDRVCSSSLPKDLAT
OsMATE36	MEGQGLVSRSWQESKLLWRVAFPAVLVELLQFSIGFVTASFVGHLGVVELAAV<br>TAVESILEGFAYGVLFGMGCALDTLCGQAVGAGRLDVLGLYVQQSWIVCGAT<br>AVALTPTYAFAEPILRSLLRQPADVAAVAGPYALWSLPRLFAHAANFPLQKFF<br>QTQSRVWALAAISAAVLAVHAALTYAAVVRLRYGLRGAAVAGNLSYWLIDA<br>AQFVYLVSGRFPDAWKGFTMTAFSNLAAFVKLSLVSAIMICLEFWYYAALLILV<br>GLLKNARLQLDIMSICINYQFWTMMVAMGFSEAISVRVSNELGARRPKEAKFS<br>VAVASLTSAMIGAIFMSIFFIWRTSLPSLFSDDKEVVDGAARLGYLLAVTVFFGNI<br>GPVLSGVAVGAGWQILVAFVNIGCYYLVGIPFGVLFGFKLKLGALGIWMGMLT<br>GTLLQMAILFFIIMRTKWEAQAILAEKRISELGETTAND
OsMATE37	MNGESLLDRSSSADAAMNDAVPGHRHHHPLSVFLRDARLAFRWDELGREIM<br>GIAVPGALALMADPVASLVDTAFIGHIGPVELAAVGVSIAVFNQVSRIAIFPLVS<br>VTTSFVAEEDATSSDREKYEINGENEFNVSDSEMEELVSHEEASAAPSKSSFETDS<br>SDVKIEHKRKNIPSVSTALLLGGVLGLLQALLLVICAKPLLGYMGVKQGSAML<br>MPALKYLVVRSLGAPAVLLSLAMQGVFRGLKDTKTPLYATVAGDATNIVLDPI<br>FMFVFQYGVSGAAIAHVISQYFIASILLWRLRLHVDLLPPSFKHMQFSRFLKNGF<br>LLLARVIAATCCVTLSASMAARLGSVPMAAFQICLQIWLASSLLADGLAFAGQ<br>AILASAFARQDHSKAAATASRILQLGLVLGLLLSIFLGIGLRLGSRLFTDDQDVL<br>HHIYLGIPFVSLTQPINALAFVFDGINYGASDFGYAAYSMILVAIVSIIFIVTLASY<br>NGFVGIWIALTVYMSLRMLAGFLRIGTARGPWTFYAAQRMHSHEVVGLC
OsMATE38	MNTLTYMLIPISWTTIQRIHHFQPNKRLTVGDSIDMDESTVEEPLLAVRRGGDG<br>EDGAMASTAAEVKRLLRLAGPLMAGFVLRNSVQMVSVMFVGHLGELQLAGSS<br>LAASLASVTGFSLLSGMSSALDTLCGQAYGAGQHRLLGVYAQRAMLVLAAAA

	VPIALVWASAGEILLLFGQDPAIAAEAGAYARWMIPSLAAYVPLACALRFLQA<br>QGIVVPVMASSGVAAVAHVAVCWALVHKAGMGSKGAALSGAVTYWTNFAV<br>LAFYARLSGACKTTWTGFSMNAFRELRRFTELAVPSAMMVCLEWSSFEILVLLS<br>GILPNPQLETAVLSISLSTASLLIMVPRGIGSSLSTRVSNELGGGHPRAARMAVRV<br>ATAMTVLVCLVLVIAMILLRNVWGYAYSNEEEVVAYIASMLPILAVSFFVDGIN<br>GALSGVLTGCGKQKIGAHVNLGAFYLVGIPTAVLLAFVLHLNGEGLWLGIVCG<br>SISKVGMLLFITLHTDWGKEVQKASPCKHFGLYHVFSGTSV
OsMATE39	MEKPAASVEEPLLVGAGEKKGESAAAAELKRLLRLAGPLVASGVLRNVVQMV<br>SVMFVGHLGELPLAGASLATSLANVTGFSLLFGMASALDTLCGQAYGARQHH<br>LLGVYKQRAMLVLAVAAVPIALVWASAGEILLLFGQDPAIAAEAGAYARWLIP<br>SLVPFVPLVCHIRFLQAQSAVLPVMASCGVTAASHVAVCWALVRKAGMGSRG<br>AALANAVSYGVNLTIMSLYVRLSRSCEKTWTGFSMEAFRELRQYAELAIPAAM<br>MVCLEWWSFEFLVMLSGLLPNPKLETSVLSICLNTGALLVMVPIGLSTAISTRVW<br>NELGAGNPQAAKLATRVVICMAMTEGSVVAFTMILLRNSWGHMYSDEAEVV<br>TYIARMIPVLAISFFIDGMHSALSGVLTGCGKQKIGARVNLGAFYLAGIPMAVFL<br>AFVLHLNGMGLWLGIVCGSLSKLILLFWITMSINWEKESTRAKELVFSSSLPVA
OsMATE4	MARSSSAPESMEADHQLTATVTAAASGDMPVTEQQQQKQMVAVAAPPATEN<br>ADAAGGGGGDNGDHLPPTATSLLGGARRTGLHLFVLNARSALRLDELGAEVL<br>RIAVPASLALTADPLASLIDTAFIGRIGSVEIAAVGVAIAVFNQVMKVCIYPLVSV<br>TTSFVAEEDAILSKGAAGADDDNDDGHDAKGHGASAAAVADPEKQQVVGV<br>DSAETNGAEVSTAAVRTTDDKKAAAAGVGVGKCRRRFVPSVTSALIVGAFLGL<br>LQAVFLVAAGKPLLRIMGVKPGSPMMIPALRYLVVRSLGAPAVLLSLAMQGVF<br>RGFKDTKTPLYATVTGDLANIALDPILIFTCRFGVVGAAIAHVISQYLITLIMLCK<br>LVRKVDVIPSSLKSLKFRRFLGCGFLLLARVVAVTFCVTLAASLAARHGATAMA<br>AFQICAQVWLASSLLADGLAVAGQALLASAFAKKDHYKVAVTTARVLQLAVV<br>LGVGLTAFLAAGMWFGAGVFTSDAAVISTIHRGVPFVAGTQTINTLAFVFDGV<br>NFGASDYAFAAYSMVGVAAVTIPCLVLLSSHGGFVGIWIALAIYMSVRAFAST<br>WRMGAARGPWKFLRK
OsMATE40	EPLLAAAPPTPGKAADGDGPEEGRRLASAEAKRLLRLAGPIVASCILQCVVNM<br>VSVMFVGHLGELPLAGASLATSLANVTGYSLLTGMATAMDTLCGQAYGARQY<br>HLLGVYKQRAMVVLAAACVPIALVWASAGRILLLLGQDAGIAAEAGAYARW<br>MLPSLAAYVPLQCHIRFLQTQTVVLPVTASSAATALLHPLVCWLLVFRAGMGS<br>KGAALANAISYAVNLAILAVYVRASNTCKGRWSGFSGEAFKELRQFAALAMPS<br>AMMICLEWWSFEILVLLSGLLPNPQLETSVLSICLNTGALLYMVPLGLCSSISTRV<br>SNEIGAGQPQAAKRATRVVMYMALSEGLVISFTMFLLRNVWGYMYSNEQEVV<br>TYIARMLPILDISFFIDGLHSSLSGVLTGCGKQKIGAAVNLGAFYLVGIPVAVLLA<br>FYLHLNGMGLWLGIVCGSIIKLLVLIIVSCCIDWEKEAILAKDRVFSSSLPVA
OsMATE41	MERTTEDDERPTVPLLEPKPASNEEEEEVGSVRRRVVEENKKLWVVAGPSICAR<br>FSSFGVTVISQAFIGHIGATELAAYALVSTVLMRFSNGILLGMASALETLCGQSY<br>GAKQYHMLGVYLQRSWLVLFCCAVILLPVYIFTTPLLIALGQDPEISAVAGTISL<br>WYIPVMFSYIWAFMLQMYLQAQSKNMIVTYLAFLNLGIHLFLSWLLTVKFQLG<br>LAGVMGSMVISFWIPVFGQLAFVFFGGCPLTWTGFSSSAFTDLGAIMKLSLSSGV<br>MLCLELWYNTILVLLTGYMKNAEVALDALSICLNINGWEMMISIGFLSAIGVRV<br>ANELGAGSARRAKFAIFNVVTTSFLIGFVLFVLFLFFRGSLAYIFTESKAVADEVA

	DLAPLLAFSILLNSVQPVLSGVAIGSGWQSVVAYVNVTSYYLIGIPLGAILGYVLGFQAKGIWIGMLLGTLVQTLVLLFITLRTNWKKQVEITRERLNRWYMDENGRSQNSIGNA
OsMATE42	EVGSVRRRVVEENKKLWVVAGPSICARFSSFGVTVISQAFIGHIGATELAAYALVSTVLMRFSNGILLGMASALETLCGQSYGAKQYHMLGIYLQRSWLVLFCCAVILLPVYIFTTPLLIALGQDPEISAVAGTISLWYIPVMFSYIWAFTLQMYLQAQSKNMIVTYLAFLNLGIHLFLSWLLTVKFQLGLAGVMGSMVISFWIPVFGQLAFVFFGGCPLTWTGFSSSAFTDLGAIMKLSLSSGVMLCLELWYNTILVLLTGYMKNAEVALDALSICLNINGWEMMISIGFLSAIGVRVANELGAGSARRAKFAIFNVVTTSFLIGFVLFVLFLFFRGSLAYIFTESKAVADEVADLAPLLAFSILLNSVQPVLSGVAIGSGWQSVVAYVNVTSYYFIGIPLGAILGYVLGFQAKGIWIGMLLGTLVQTLVLLFITLRTDWKKQVEITRERLNRWYMDENGRSQNSIGNA
OsMATE43	MTATSPPMRSVAAAALVLTPTPTLNRLSFPFAHRHCPSTAAPRWRPARCRGKPAVEDVVHDDEEETWRREANPERKDGGEEMLGRGWFMVDEIGMEILTIALPAVLALAADPITALIDTAFVGHVGSTELAAVGVSISIFNLVSKLLNVPLLNVTTSFVAEQQAVDADYNSSVENSHIGEEISISQEKAGEQRKFLPAVSTSLALAAGIGLMETVALILGSGTLLDIVGVPVDSPMRIPAEQFLTLRAYGAPPVIVALAAQGAFRGFMDTKTPLFAVVAGNLVNALLDAIFIFPLGLGVSGAALATVTSEYLTAFILLWKLNSKIVLFSWNIVSGDIIRYLKSGALLIARTIAVVLTFTVSTSLAAREGSVPMAGYEICLQVWLTISLLNDALALAGQALLASEYAKGNYKKARIVLYRVLQIGGVTGAALSTTLLLGFGYLSMLFTDDAAVLDVAQTGVWFVTVSQPINAVAFVMDGLYYGVSDFAFVAYSTLFAGAISSAVLLVAAPKFGLGGVWAGLTLFMSLRAIAGFWRLGSKGGPWKIIWSETE
OsMATE44	MERPGDEHDDCRTAPLLEPKHAHGEGSNNDKQEEDEEEVGSLGPRVLVESKKLWVVAGPSICARFSTFGVTVISQAFIGHIGATELAGYALVSTVLMRFSGGILLGMASALETLCGQSYGAKQYHMLGIYLQRSWIVLLCCAVLLLPIYLFTTPLLIFLGQDPKIAAMAGTISLWYIPVMISNVGNFTLQMYLQAQSKNMIVTYLAMLNLGLHLFLSWLLTVQFHLGLAGVMGSMVIAYWIPVFGQLAFVFFGGCPLTWTGFSSAAFTELGAIVKLSLSSGVMLCVELWYNTILVLLTGYMKNAEIALDALSICLNINGWEMMISIGFLSATGVRVANELGAGSARRAKFAIFNVVTTSFSIGFMLFVLFLIFRGRLAYIFTESKVVADAVAELSPLLAFSILLNSIQPVLSGVAVGSGWQSVVAYVNVTSYYLFGIPIGVILGYVLGFQVKGIWIGMLLGTLVQTIVLLFITLRTDWEKQVEIARQRLNRWSMDENGRQQNPGENP
OsMATE45	MEELKLMRRLCLPISALNLLHYVKSMVTVLCLGRLGRAELAGGALAVGLTNVTGYSVLSGLALGLEPLAGQAFGSGTGRTRSRPRRALRRAVLLLLAASFPVAALWACAGPAARAARQDAAVARAAGSYCRYAIPDLAAASVLLPARVYLRSKGETRRLASCAALAVALVHAPATAYLGARLRVPGVAMAACMTSFATLAFLWISLTWAPAQNEPDEPADLEEWAGVGQWAEWADLLRLSLPSCLSVCLEWWWYELMTIAAGYLRDPHTALATAAIVIQTTSLLYTIPVTLSSAVSTRVANELGAGRPRSAQVSFVVAMGIAMMGSCVGLTWTTFGRGLWVQVFTTDPTIQSLTTSVLPVIGLCELANCPQTTGCGVLRGSARPAVGAAINLYSFYLVGAPVALVLAFGLDMGFLGLCLGLLSAQVVCLISVGFATFQTDWEAEALKAFHLVGGGDEKCGDDLPCLAHKENV
OsMATE46	MAETSSARSPLLDVDESSGASEELLRREPVPRSVLSRLAAWEAGNLWRISWASILITLLSFTLSLVTQMFVGHLGELELAGASITNIGIQGLAYGIMIGMASAVQTVCGQ

	AYGARKFRAMGIVCQRALVLQFATAIVIAFLYWYAGPFLRLIGQAADVAAAG<br>QLYARGLVPQLLAFALFCPMQRFLQAQNIVNPVAYITMAVLIFHILISWLTVFVL<br>GFGLLGAALTLSFSWWVLVALTWGLMVWTPACKETWTGLSVLAFRGLWGYA<br>KLAFASAVMLALEIWYVQGFVLLTGFLPDPEIALDSLSICINYWNWDFQIMLGL<br>SYAASIRVGNELGAGHPNVARFSVFVVITASVAFSILATILVLVLRYPLSTLYTSST<br>TVIEAVIKLTPLLSISIFLNGIQPILSGVAVGSGWQVVVAYVNVGAYYLIGLPIGC<br>VLGYKTSLGAAGIWWGLIIGVSVQTVALIIITARTNWDNEVMKAIQRLRQTAVD<br>DGTVPIVDDIE
OsMATE5	MSAHLRLLSAAPLPALLPTRRLPAVPTPALAARAARLVLSRPLTEPQPPRLPRG<br>AAARCRGVAVAGADGDEPAAAAAGTAGLWEQVRDIVVFAGPALGLWICGPL<br>MSLIDTMVIGQTSSLQLAALGPGTVFCDYLCYIFMFLSIATSNMVATSLAKKDEE<br>LAQHQVSMLLFVALTCGLGMFLFTKLFGTQVLTVFTGSGNYDIISAANTYAQIR<br>GFAWPAVLVGLVAQSASLGMKDSWGPLKALAAASVINGVGDLLLCSVCGYGI<br>AGAAWATMVSQIVAAFMMMQNLNKRGFRAFSFTIPSSSELLQIFEIAAPVFITM<br>TSKVAFYALLTYSATSMGAITLAAHQVMVNVLCMCTVWGEPLSQTAQSFMPE<br>LIYGAKCNLMKARMLLKSLVMIGAITGTTVGAVGTLVPWLFPSLFTNDFMVVQ<br>QMHKVLIPYFCALLVTPSVHSLEGTLLAGRDLRFLSQSMGACFGIGTFLLMV
OsMATE6	MSSPRRDGRGAVDDLTASLLHKGDGGEAVFVVVVVPPVAEEEEPPPVLTCKPP<br>GRFARAVKEAWSVPFPMMPSMSAGAAGAEARSILGLALPMILTGLLLYLRSMIS<br>MLFLGRLGGLALAGGSLAIGFANITGYSVLSGLAMGMEPICGQAFGAGHYDLL<br>GVTMQRTVLLLVAASVPIAGLWVHMRPLLLLCGQDAAIAAVAETYILASLPDL<br>LLQAFLHPVRIYLRTQSINLPLTVCAALAIALHLPINYVAVSVLGLGIKGVALAS<br>VLANLNLVLFLFGYIWFKGVHKRTGGFALSADCLRGWGELVSLALPSCISVCLE<br>WWWYEIMILLCGLLANPQATVASMGILIQTTSLIYIFPSSLGFGVSTRVSNELGA<br>NRPERACRAATVGLMLGFAFGGVASAFACHVRGAWATMFTADPAIVALTAS<br>VLPILGACELGNCPQTTGCGVLRGSARPKDAASINLRSFYLVGTPVALILAFWY<br>HYDFRGLWLGLLAAQATCVVRMLLVIGETDWTAEAKRAQQLTGAADIKDCG<br>GKGDHVAVIEQPDEQC
OsMATE7	MAYTEPLLLSARHTTQTSPRHHLLRSRHAAAAAADGRMVVAVQDDETGALV<br>AAVGKGDEDDDDDDAVAGEEDEDEDDAPVVRTARGAWEVFAAESRRLWAI<br>GAPIAFNVICLYGTNSTTQIFVGHIGNRELSAVAIGLSVVSNFSFGFLLGMGSALE<br>TLCGQAFGAGQVAMLGIYMQRSWIILAASAALLSPLYVFAGPILRLLGQEESIA<br>AAAGEFTVRIIPQMFALAINFPTQKFLQAQSKVTVLAWIGFAALLAHVGLLALF<br>VSALGWGIAGAAAAYDVSSWLTALAQVAYVVGWCRDGWTGLSRKAFNELW<br>AFVKLSLASAVMLCLEIWYMMVLVVLTGHLDDAEIAVDSISICMNINGWEGML<br>FIGLNAAISVRVSNELGSGRPRATMHAVVVVLVQSLAFGLLAMVLILATRNHF<br>AVIFTGDRHLQKAVANIAYMLAVTMVLNSIQPVWRSAADGREWWRTLIWRVT<br>TASASLSASYLATSSDGV
OsMATE8	MGDGRGDEEECRVALLNGGGAAKEGWQVVSGGDGKLRRRVWEESRKLWVIV<br>APAIFSRVVTYSMNVITQAFAGHLGDLELAAISIANTVVVGFNFGLMLGMASAL<br>ETLCGQAFGAKKYHMMGVYMQRSWIVLLACAVLLLPMYIYAEDVLLLTGQPP<br>ELSAMAGRVSVWFIPLHLSFAFLFPLQRFLQCQMKNFASAAASGVALCVHVAIS<br>WLLVSRFRFGLVGIALTLNFSWWATAAMLFAYVACGGCPETWNGLSLEAFAG<br>CHAMLLTEFVCLICSLENWYYRILILLTGNLKNAAIAVDALSICMTINAWELMIP

OsMATE9	LAFFAGTGVRVANELGAGNGKGARFATIVSSVTSLVIGLFFWVLIVGLHDKFAL IFTSSDVVLDAVDNLSVLLAFTILLNSIQPVLSGVAVGSGWQSMVAYVNIGTYYL IGIPMGILLGWLFKLGVLGIWAGMIGGTAVQTLILAIITIRCDWDKEAMIASTRM DKWSQVR MAGLKKMEEVTAAAAAVAASSTAEKRAAAVVVPDAALTMNGAAGAEEKTA AAAAAPEDLPAPAALSGWPRRVGLYLFVMNIRSVFKLDELGSEVLRIAVPASLA LAADPLASLVDTAFIGRLGSVEIAAVGVSIAIFNQVSKVCIYPLVSVTTSFVAEED AIISKCIEENSSQDLEKASPVDSETNNLPVSGPDKVECVNSCIPTECTNPSDQGCK RKYIPSVTSAVIVGSFLGLLQAVFLVFSAKFVLNIMGVKNDSPMLRPAVRYLTIRS LGAPAVLLSLAMQGVFRGFKDTKTPLYATVVGDAANIILDPILMFVCHMGVTG AAVAHVISQYLITMILLCRLIRQVDVIPPSLKSLKFGRFLGCGFLLLARVVAVTFC VTLASSLAARHGPTIMAAFQICCQLWLATSLLADGLAVAGQAVLASAFAKND KGKVVVATSRVLQLSIVLGMGLTVVLGVGMKFGAGIFTKDIDVIDVIHKGIPFV AGTQTINSLAFVFDGINFGASDYTYSAYSMVGVAAISIPCLVYLSAHNGFIGIWI ALTIYMSLRTIASTWRMGAARGPWVFLRK

Table S4. The primers used in this study

Name	Primer sequence
Primers for Gene cloning and Construction of expression vector	
GmMATE13-F	5'-ATGATGCCCCTTTTGATGTTAT-3'
GmMATE13-R	5'-CTAAAGCCCAACATTGTTTACC-3'
GmMATE75-F	5'-ATGGACGAGAATAGAAGTTCCAAC-3'
GmMATE75-R	5'-TCACTAAGGCAGTGAGCGACCTC-3'
Primers for qRT–PCR in tamba black soybean	
q18S rRNA-F	5'-ATGATAACTCGACGGATCGC-3'
q18S rRNA-R	5'-CTTGGATGTGGTAGCCGTTT-3'
qGmMATE13-F	5'-CTCCCTACTACTCTGGCTTTGGC-3'
qGmMATE13-F	5'-TTGTTCATCAGCAGCATCTTCC-3'
qGmMATE75-F	CCAACGAACCCAACAAGTGG
qGmMATE75-R	CCGGTCCTAAACGGCCTATG
Primers for subcellular localization	
GmMATE13-F-XbaI	5'-GCTCTAGAATGATGCCCCTTTTGATGTTAT-3'
GmMATE13-R-SmaI	5'-TCCCCCGGGAAGCCCAACATTGTTTACC-3'
GmMATE75-F-XbaI	5'-GCTCTAGAATGGACGAGAATAGAAGTTCCAAC-3'
GmMATE75-R-SmaI	5'-TCCCCCGGGCTAAGGCAGTGAGCGACCTC-3'
